# Phenomic and Physiological Analysis of Salinity Effects on Lettuce

**DOI:** 10.3390/s19214814

**Published:** 2019-11-05

**Authors:** Neil D. Adhikari, Ivan Simko, Beiquan Mou

**Affiliations:** Crop Improvement and Protection Research Unit, United States Department of Agriculture, Agricultural Research Service, Salinas, CA 93905, USA; ivan.simko@usda.gov

**Keywords:** phenomics, high-throughput, lettuce, salinity, physiology, phenotyping

## Abstract

Salinity is a rising concern in many lettuce-growing regions. Lettuce (*Lactuca sativa* L.) is sensitive to salinity, which reduces plant biomass, and causes leaf burn and early senescence. We sought to identify physiological traits important in salt tolerance that allows lettuce adaptation to high salinity while maintaining its productivity. Based on previous salinity tolerance studies, one sensitive and one tolerant genotype each was selected from crisphead, butterhead, and romaine, as well as leaf types of cultivated lettuce and its wild relative, *L. serriola* L. Physiological parameters were measured four weeks after transplanting two-day old seedlings into 350 mL volume pots filled with sand, hydrated with Hoagland nutrient solution and grown in a growth chamber. Salinity treatment consisted of gradually increasing concentrations of NaCl and CaCl_2_ from 0 mM/0 mM at the time of transplanting, to 30 mM/15 mM at the beginning of week three, and maintaining it until harvest. Across the 10 genotypes, leaf area and fresh weight decreased 0–64% and 16–67%, respectively, under salinity compared to the control. Salinity stress increased the chlorophyll index by 4–26% in the cultivated genotypes, while decreasing it by 5–14% in the two wild accessions. Tolerant lines less affected by elevated salinity were characterized by high values of the chlorophyll fluorescence parameters Fv/Fm and instantaneous photosystem II quantum yield (QY), and lower leaf transpiration.

## 1. Introduction

Global agriculture is under increasing threat from rising levels of salinity in soil and irrigation water. Salinity affects soil at nearly 10–20% of all land surface but is particularly problematic at irrigated land where almost 50% of all area is affected [1,2]. Rising salinity in soils may be exacerbated by increasing temperatures, rising seawater levels, intrusion of seawater, erosion of minerals, and human activities such as over-fertilization and over-watering [3,4]. Economic analyses suggest that the global annual cost of salt-induced land degradation in irrigated areas due to lost crop production could be US $27.3 billion in 2013 [5].

Lettuce is an economically important vegetable crop cultivated in many countries around the world, with the highest production value in the U.S., Europe, and China [6]. It is an important source of vitamins, carotenoids, antioxidants, and other phytonutrients [7,8]. In the U.S., lettuce is the most consumed salad vegetable and economically the most important leafy green vegetable [6]. Lettuce, like most crop plants, is sensitive to salinity [9], with growth stunting starting at soil electrical conductivity (EC) around 2000 µS cm^−1^ [10]. Salinity at this and higher levels reduces plant and root growth, seed germination, and leaf water content, while increasing the sodium and chloride ion concentration and lipid peroxidation [11,12,13,14,15,16,17,18]. To better understand plant responses to salinity, studies of plant adaptation at physiological, molecular, and biochemical levels continue both on model plants and cultivated crops [19,20,21,22,23,24,25,26,27].

Measurement of chlorophyll *a* fluorescence kinetics has become an important tool in plant science, which allows non-invasive determination of plant health [28,29,30]. Photosystem I (PSI), photosystem II (PSII) and the light harvesting complexes associated with them exist as protein-pigment complexes with chlorophyll [31]. Chlorophyll is highly efficient at absorbing light energy, which is then either used in photosynthesis, re-emitted as heat (non-photochemical quenching (NPQ)) or fluorescence. Photosynthesis competes with heat and fluorescence for light energy, and this competition allows determining the efficiency of PSII. In a normal, healthy photosynthetic system, most of the absorbed light energy is used in photosynthesis, and only a fraction is lost to heat dissipation and fluorescence. When photosynthesis is performing at its optimal efficiency, fluorescence is minimum, and vice versa. Measuring chlorophyll fluorescence allows the evaluation of photosynthetic efficiency and heat dissipation. Transfer of a normal photosynthesizing system from light to dark for a period of time, usually lasting 30 min, is known as dark adaptation. The Kautsky effect [32,33] is the rapid rise in chlorophyll *a* fluorescence of a dark-adapted photosynthetic system upon illumination with an actinic light source, followed by a gradual decline, ultimately reaching a steady state. Measurement of these transitions from a dark-adapted state to light-adapted state and back, representing the complex dynamics between chlorophyll fluorescence and photochemical yield, allows studying the photosynthetic reactions in great detail. After dark adaptation, the photosynthesis reaction centers are considered open and available for photosynthesis at their full capacity. In this state, the chlorophyll fluorescence is minimal, also called F_0_, and photosynthetic yield is essentially zero [34]. If a strong pulse of light is applied in this state, it transiently overloads and clogs the photosynthetic electron transport pathway, resulting in closing of the reaction centers. Thus, photosynthesis being at zero, all the light energy is now diverted into fluorescence. This is called maximum fluorescence (Fm). A few seconds of dark relaxation reopens the reaction centers, following which, application of actinic light drives photosynthesis. In the first few seconds following application of actinic light, the photoprotective mechanisms and photosynthetic machinery both are inactive, and the fluorescence measured in this state is called peak fluorescence (Fp). Fluorescence steadily decreases after a few minutes of adaptation in this condition, as the electron flow in PSII increases and more and more light energy is diverted to photosynthesis than fluorescence. The fluorescence measured just before actinic light is switched off, is called steady-state fluorescence in the terminal light-adapted state (Ft_Lss). These are the basic measurements used for the calculation of many other parameters related to photosynthesis (Supplemental Data 1), [29,31,32,33,35,36,37]. The difference between F_0_ and Fm is the variable fluorescence, Fv. The ratio of Fv/Fm has been shown to be a robust indicator of the maximum quantum yield of PSII chemistry [38,39]. Fv/Fm values for healthy, unstressed leaves are highly consistent, at ~0.83 [40]. Damage to PSII, also referred to as photoinhibition, results in the lowering of Fv/Fm, and is widely used as an indicator of measuring stress in leaves. NPQ involves many different mechanisms, but essentially is responsible for safely dissipating excess chlorophyll excitation to prevent the formation of reactive oxygen species [41]. Coefficient of non-photochemical quenching (qN) is another parameter used to calculate non-photochemical quenching. Coefficients of photochemical quenching, qP and qL, measured using two different methods, are used to estimate the proportion of open PSII reaction centers. Each of the two methods has certain advantages and disadvantages, which need to be taken into consideration while interpreting these estimations [42,43]. Instantaneous PSII quantum yield of PSII photochemistry (QY) parameter reports the PSII quantum yield during dark relaxation, light induction, or steady-state light [44]. The ratio of fluorescence decline (Rfd) is used to calculate the photosynthetic capacity and CO_2_ fixation rate, and is also referred to as plant vitality [45,46,47].

Recent advances in sensing technology have led to the development of precision phenotyping instruments with highly accurate, fast operating sensors and streamlined, automated, high-throughput workflows [48,49]. The new phenotyping systems have been able to address many of the challenges [49] related to phenotyping of crop plants, such as size, location, weight, quantity, and health status [50]. In lettuce research, optical sensors were previously used to evaluate post-harvest quality [51,52] and reaction of plants to suboptimal and supraoptimal temperatures [18], elevated salinity [18], and ultraviolet radiation [53]. The present study builds on previous lettuce studies and adds more detailed phenomic observations and analyses of changes occurring in plants grown under elevated salinity.

Cultivated lettuce is classified into several horticultural types with distinct phenotypes. Prickly lettuce (*L. serriola*) is a wild lettuce sexually compatible with cultivated lettuce. This species is used in breeding programs as a donor of certain desirable genes into cultivated lettuce [6]. Previously, approximately 3800 lettuce cultivars and accessions from the USDA germplasm collection were screened for sensitivity or tolerance to salinity [54]. A total of 178 cultivars and accessions were selected from this initial screen and retested under greenhouse conditions for the effect of salinity on fresh weight (FW), dry weight (DW), chlorophyll index, and chlorophyll fluorescence. Lettuce cultivars were classified into sensitive or tolerant categories, based on their reduction in FW under salinity compared to control conditions [54]. Using this information, we selected a contrasting set of the most sensitive or most tolerant genotypes from each lettuce type for a more detailed study. Because environmental factors can significantly affect gene expression, protein levels, photosynthesis, and chlorophyll activity in plants [55,56,57,58,59], lettuce was grown in controlled conditions of growth chambers. Plants were evaluated using a combination of non-invasive methods, including high-throughput phenomics and invasive lab analyses, to examine their health, photosynthetic capacity, and overall performance. The main goal of this study was finding unique physiological characteristics within each lettuce type which may be important in salinity tolerance, as well as any common characteristics shared by a majority of tolerant genotypes across the five lettuce types, which may distinguish them from the sensitive ones. A secondary goal was to establish the baseline physiological parameters of selected tolerant and sensitive lettuce genotypes under controlled conditions for use in future research aimed at studying responses to modified environmental conditions at the cellular and molecular level.

## 2. Materials and Methods

### 2.1. Plant Materials

Lettuce cultivars and germplasm accessions used in this study were: “Laura” (crisphead type, tolerant to salinity, abbreviated in tables and figures as LAU), “Early Bird” (crisphead, sensitive, EAB), “Morgana” (butterhead, tolerant, MOR), “Mayfair” (butterhead, sensitive, MAY), “Eruption” (Latin, tolerant, ERU), “Parris Island Cos” (romaine, sensitive, PIC), PI 171676a (leaf, tolerant, P17), “Shining Star” (leaf, sensitive, SHI), PI 253468 (*L. serriola*, tolerant, P25), and PI 491154 (*L. serriola*, sensitive, P49). “Eruption” is a Latin type cultivar, but due to similarity of Latin and romaine types they can be directly compared. Cultivars and accessions from the same/similar horticultural type (or wild species) were compared to each other in order to minimize the effect of horticultural types on observed differences in their performances. Classification into salinity tolerant and sensitive groups were based on a previous study [54].

### 2.2. Growth Conditions

Seeds were germinated in distilled water in sterile Petri dishes for two days to ensure synchronized and uniform germination. Three uniform, 2-day-old seedlings from each cultivar were then transplanted into pots. Humidity domes were used for one week to help survival of seedlings after transplantation. After the first week, seedlings were thinned to keep only one per pot. All plants were grown in a Conviron CMP6050 growth chamber (Conviron, Winnipeg, MB, Canada) at 20 °C, 200 µmol m^−2^ s^−1^ continuous white light, and relative humidity between 50–70%. Trays were rotated every two days to ensure that all plants experienced the same micro-environment variations during their growth. Sterilized sand in ~10 cm pots (350 mL volume) lined with coffee filters, was used as the growth substrate. Pots were placed in “1020 trays” (53.34 cm × 27.3 cm × 6.35 cm) to accommodate 18 pots per tray. Control plants were watered with Hoagland nutrient solution without NaCl (EC 2093 µS cm^−1^), while treatment plants were watered with NaCl/ CaCl_2_ -containing Hoagland solution. Salt-stress was gradually introduced so as to prevent salt-shock. To plants in the treatment trays, Hoagland solution containing 10 mM/ 5 mM NaCl/ CaCl_2_ (EC 5690 µS cm^−1^) was applied right after transplanting, followed by 20 mM/ 10 mM NaCl/ CaCl_2_ (EC 6320 µS cm^−1^) in the second week and 30 mM/ 15 mM NaCl/ CaCl_2_ (EC 8620 µS cm^−1^) in weeks 3 and 4. Trays were watered by reverse irrigation to avoid leaf injury due to contact with high-salt solutions. During watering, 3 L of the respective solution was applied to each tray and allowed to soak for 30 min. Excess liquid was then discarded from the trays.

Before the beginning of the experiment, salinity in sand, tap water, distilled water, and Hoagland solution [60] was measured with an EC meter (Orion 3-Star Benchtop Conductivity meter, Thermo Scientific, Waltham, MA, USA). Sand from two sources had EC of 500 µS cm^−1^ (measured using the Soil Test Direct Conductivity Tester, Hanna Instruments, Smithfield, RI, USA), which is a low salinity for lettuce. The EC of tap water was 800 µS cm^−1^, while that of distilled water was 1 µS cm^−1^. Therefore, for accuracy and consistency of salt concentrations, all solutions were prepared using distilled water. Sand moisture during experiments was tracked using ECH2O EC-5 soil moisture probes (Meter group, Pullman, WA, USA). Pots were always kept moist to avoid sudden and drastic increases in salinity due to drying, and were not allowed to fall below 0.1 m^3^m^−3^ water/soil volumetric water content (VWC).

Experiments were repeated up to five times, with each experiment consisting of at least four biological replicates per cultivar/accession per condition in a completely randomized design.

### 2.3. Measurements

All physiological measurements were conducted four weeks after transplanting, just before the first plants reached their bolting stage. The leaf chlorophyll index was measured with a SPAD-502m (Konica Minolta Sensing Inc., Tokyo, Japan) hand-held meter. Measurements from five intermediate-aged leaves of similar age per plant were averaged. Photosynthetic CO_2_ assimilation was measured using the Licor LI-6400 XT Photosynthesis System (Licor, Lincoln, NE, USA). Photosynthesis measurements were performed on three leaves of intermediate age per plant and averaged. Light intensity was set to 200 µmol m^−2^ s^−1^, and 50–70% relative humidity, similar to that experienced by plants in the growth chamber. Flow rate was set at 400 µmol m^−2^ s^−1^ and 400 ppm CO_2_, at near-atmospheric levels, was supplied for photosynthesis. Light intensities were measured using the LiCor LI-250A Light Meter (Licor, Lincoln, NE, USA). Plant FW was determined by cutting off the entire plant at the base (root junction) and immediately weighing. After determining FW, plants were dried at 40 °C for one week and weighed to determine their DW.

### 2.4. Chlorophyll Fluorescence Imaging: Data Acquisition and Analysis

Chlorophyll *a* fluorescence was measured using the PlantScreen^TM^ Transect XZ system, a custom setup based on the PlantScreen^TM^ Robotic XYZ system (Photon Systems Instruments, Drasov, Czech Republic; http://plantphenotyping.com/products/plantscreen-robotic-xyz-system/#details) consisting of an imaging station mounted on a robotic arm with an LED light panel and charged coupled device (CCD) camera positioned in the middle of the light panel (Supplemental Figure S1A). The robotic arm, which moves along the X-axis over 2 m, covered 12 trays, each with 20, 10-cm pots, for a total of 240 samples (Supplemental Figure S1B). Calibrations for converting pixels to area at the 240 mm height setting, and setting the robotic arm travel distance for each tray for automated data acquisition were performed using instructions provided by the manufacturer. Acquisition and analysis of fluorescence data were performed using FluorCam7 Software (Photon Systems Instruments, Drasov, Czech Republic). Quenching protocol, consisting of a modulated light of known wavelength, was used for all chlorophyll fluorescence measurements, to measure the Kautsky effect in the pulse-amplitude modulated (PAM) mode (Supplemental Figure S1C). Light sources consisted of three types, (1) PAM short duration measuring light (red-orange, 620 nm, with 33 µs flashes), (2) actinic light (red-orange, 620 nm) with maximum light intensity of approximately 500 µmol m^−2^ s^−1^, and (3) saturating cool-white light with maximum intensity of approximately 5000 µmol m^−2^ s^−1^. Actinic light intensity was set to 50%, corresponding to approximately 250 µmol m^−2^ s^−1^, similar to that experienced by plants in the growth chamber. Far-red light and saturating light intensities were set at 30% and 20%, respectively. Total intensity of the saturating actinic light pulse at these settings corresponded to approximately 1200 µmol m^−2^ s^−1^. All measurements were performed after 30 min of dark adaptation. Data acquisition was staggered, including a 5 min dark adaptation after every measurement, in order to prevent light overflow to adjacent trays during measurement, potentially affecting dark adaptation. Electronic shutter was set at 20 µs and sensitivity at 10%, to maintain measurements within a dynamic range and prevent pixel overflow. Minimum fluorescence in the dark-adapted state (F_0_) was measured using a 5 s light flash. This was followed by a saturation pulse of 1000 µmol m^−2^ s^−1^ for 800 ms to determine maximum fluorescence in the dark-adapted state (Fm). Following a 17 s dark relaxation step, actinic light was turned on for 70 s to drive photosynthesis and measure the rise in peak fluorescence (Fp) during the initial phase of the Kautsky effect. During this period, saturation pulses were applied at 8, 18, 28, 48 and 68 s intervals as indicated in Supplemental Figure S1C, corresponding to L1, L2, L3, L4 and Lss states, respectively. A far-red pulse was applied immediately after the actinic light is switched off, resulting in rapid re-oxidation of the plastoquinone pool and the quinone acceptor (Q_A_)and allowing us to measure minimum fluorescence in the light-adapted state (F_0__Lss). Actinic light period was followed by dark relaxation, maintained for 100 s. Dark relaxation responses were measured during this time by applying saturating light pulses at 30, 60, and 90 s, corresponding to D1, D2, and D3 states, respectively (Supplemental Figure S1C). Each saturating pulse was followed by a short far-red light pulse to measure the instantaneous F_0_ level. The level of chlorophyll fluorescence just before application of the saturation pulse was recorded as steady-state fluorescence in the light-adapted state (Ft). Automation of plant masks for data analysis was difficult because of the large variation of shapes and sizes of the different lettuce genotypes (Supplemental Figure S1B) and due to the salinity treatment. Thus, leaf masks were drawn manually, using the lasso tool in Fluorcam7 for accurate data analysis (Supplemental Figure S1D). Background subtraction and parameter calculation was performed automatically by Fluorcam7 based on the F_0_, Fm, Fp, and Ft measurements, which were estimated by integrating values from each pixel across the entire leaf area (Supplemental Figure S1E). Chlorophyll fluorescence parameters described in this study, formulae, names, and descriptions are provided in Supplemental Data 1.

### 2.5. Statistical Analysis

All statistical analyses were conducted using R [61]. Pairwise comparisons between plants grown in control and treatment conditions were performed by the Student’s *t*-test, packages ggplot2 [62] and ggpubr [63]. Multiple contrast tests (MCTs) were conducted using Analysis of Means (ANOM) and Analysis of Variance (ANOVA). Data was fit to a linear model using the R package multcomp [64], and subjected to the generalized linear hypothesis test (GLHT) and multiple comparisons test. The R package ANOM [65] was used to identify accessions significantly (*p* ≤ 0.05) different from the overall mean. In addition, group means were also analyzed by Analysis of Variance (ANOVA) for Type II sum of squares, using the R package Companion to Applied Regression (car) [66] and applying Tukey post-hoc comparison of least-square means. Multivariate analysis was conducted by Principal Component Analysis (PCA) to identify factors explaining observed variance, and correlation analysis to identify association between multiple variables. R packages ggbiplot [67] and corrplot [68] were used to visualize PCA and correlation matrices, respectively.

#### Percent Change and Leaf Thickness Calculations

All percent change calculations were performed as percent reduction in “salt” relative to “control”, using the following formula: ((control–salt)/control) * 100. Thus, negative numbers indicate an increase in “salt” values relative to the “control”, while positive numbers indicate a decrease in “salt” values relative to the “control”. 

Calculated leaf thickness was determined using the FW/LFA (fresh weight/total leaf area) formula [69], which has a strong correlation with measured (actual) thickness of laminar leaves.

## 3. Results

### 3.1. Tolerant Lettuce Cultivars and Accessions Exhibit Less Reduction in Biomass under Salinity Compared to Control Conditions

In control conditions, FWs ranged from 2.4 g (PI 253468) to 14.8 g (“Shining Star”) (Table 1) with FW of “Mayfair”, “Eruption”, PI 253468, and PI 491154 being significantly lower than the overall mean, and FW of “Early Bird” and “Shining Star” being significantly higher than the overall mean (Supplemental Figure S2A). FW under salinity ranged from 2.0 g (PI 491154) to 9.2 g (PI 171676a) (Table 1), with FW of “Laura”, “Eruption”, PI 253468, and PI 491154 significantly lower than the overall mean and FW of PI 171676a and “Shining Star” significantly higher than the overall mean (Supplemental Figure S2B). “Laura”, “Early Bird”, PI 171676a, and “Shining Star” FW’s were significantly higher than PI 253468 and PI 491154 under control conditions (Supplemental Figures S2C and S1F–I). Under salinity, PI 171676a and “Shining Star” FW’s were significantly higher than most genotypes, except “Early Bird” (Supplemental Figure S2D). FW was significantly lower under salinity compared to control in most genotypes except PI 171676a and PI 253468. “Early Bird”, “Mayfair”, “Parris Island Cos”, PI 171676a, and PI 253468 (16% loss) showed proportionally the smallest weight loss (were the most tolerant), whereas “Laura” (67% loss), “Morgana”, “Eruption”, “Shining Star”, and PI 491154 showed proportionally the largest weight loss and were considered to be most sensitive to salinity.

DWs ranged from 0.76 g (PI 253468) to 2.49 g (PI 171676a) in control conditions, and from 0.59 g (PI 491154) to 1.91 g (PI 171676a) under salinity (Table 1, Supplemental Figure S3). PI 253468 had no significant change in DW under salinity compared to control, whereas the greatest significant decrease was 50% in PI 491154. Decreases in DW under salinity compared to the control were statistically significant only in “Laura”, “Mayfair”, “Parris Island Cos”, and PI 491154, and ranged from 22% to 50% (Table 1). Based on relative decrease in FW or DW under salinity compared to control, PI 253468 was the most tolerant of all genotypes, having the least decrease in biomass, followed by “Shining Star”, “Mayfair”, PI 171676a, “Early Bird”, “Morgana”, “Eruption”, “Parris Island Cos”, “Laura”, and PI 491154 (the most sensitive).

### 3.2. Reduction in Total Leaf Area in Tolerant Lettuce Cultivars Is Less Severe under Salinity Compared to the Control

**Figure 1:**
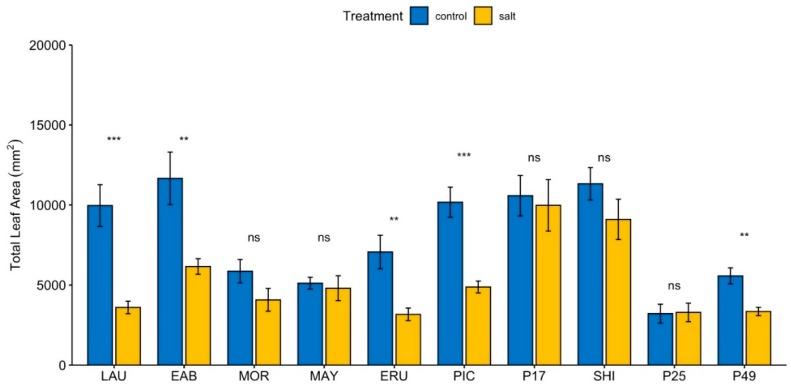
Effect of salinity on lettuce leaf area. Total leaf area was calculated using the PlantScreen phenotyping system. Values are means of two independent experiments, each with at least four biological replicates. Bars represent standard errors; asterisks indicate statistical significance as calculated by the *t*-test. * (*p* ≤ 0.05), ** (*p* ≤ 0.01), *** (*p* ≤ 0.001), **** (*p* ≤ 0.0001), ns = not significant (*p* > 0.05).

LFA (also referred to as “area”) was calculated using the PlantScreen system. The smallest total leaf area in control was observed in PI 253468 (Figure 1), whereas “Laura”, “Early Bird”, “Parris Island Cos”, PI 171676a, and “Shining Star” areas were similar, and greater than the overall mean, though not significantly so (Supplemental Figure S4A). Under salinity, PI 171676a and “Shining Star” areas (Supplemental Figures S1H and S1I) were significantly greater than the overall mean, whereas “Laura”, “Eruption”, PI 253468, and PI 491154 were significantly lower than the overall mean (Supplemental Figure S4B). “Early Bird” area was the most significantly different compared to PI 253468 under control conditions (Supplemental Figures S4C, Supplemental Data 3). Under salinity, PI 171676a area was the most different compared to all genotypes except “Shining Star” (Supplemental Figure S4D, Supplemental Data 4). Relative reduction in area was the smallest in PI 253468, with no significant change, whereas the greatest, significant reduction was in “Laura” (64%). “Early Bird”, “Mayfair” and PI 253468 have the least decrease in total leaf area than sensitive counterparts from the same types of lettuce “Laura”, “Morgana” and PI 491154. “Eruption” and “Parris Island Cos” both had similar decreases in total leaf area, although the decrease in only “Parris Island Cos” was statistically significant. Differences in PI 171676a and “Shining Star” area between control and salinity treatments were not significantly different.

### 3.3. Salinity Leads to an Increase in Chlorophyll Index in Cultivated Lettuce Cultivars

**Figure 2:**
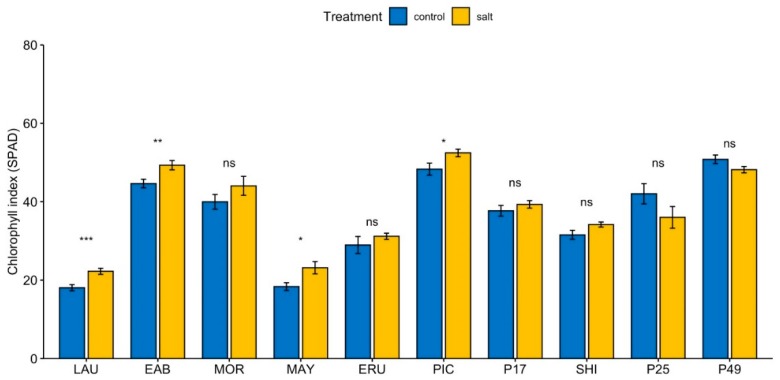
Effect of salinity on chlorophyll index in lettuce leaves. The chlorophyll index (SPAD) was measured from the same relative area from five leaves of similar age from each plant and averaged for one reading. Values are means of five independent experiments, each with at least four biological replicates per cultivar/accession per condition. Bars represent standard errors; asterisks indicate statistical significance as calculated by the *t*-test. * (*p* ≤ 0.05), ** (*p* ≤ 0.01), *** (*p* ≤ 0.001), **** (*p* ≤ 0.0001), ns = not significant (*p* > 0.05).

The chlorophyll index (SPAD) under control conditions was significantly higher than the overall mean in “Early Bird”, “Parris Island Cos”, and PI 491154, and significantly lower than the overall mean in “Laura”, “Mayfair”, “Eruption”, and “Shining Star” (Supplemental Figure S5A). Under salinity, SPAD in “Early Bird”, “Parris Island Cos”, and PI 491154 was significantly greater, and in “Laura”, “Mayfair”, “Eruption” and “Shining Star”, significantly lower than the overall mean (Supplemental Figure S5B). Application of moderate salinity stress resulted in a 4% to 26% increase in SPAD at the end of the 4-week growth period in lettuce cultivars (Figure 2), but a 5% to 14% decrease in wild *L. serriola* accessions PI 491154 and PI 253468.

### 3.4. Photosynthetic CO_2_ Assimilation and Vapor Pressure Deficit Based on Leaf Temperature

**Figure 3:**
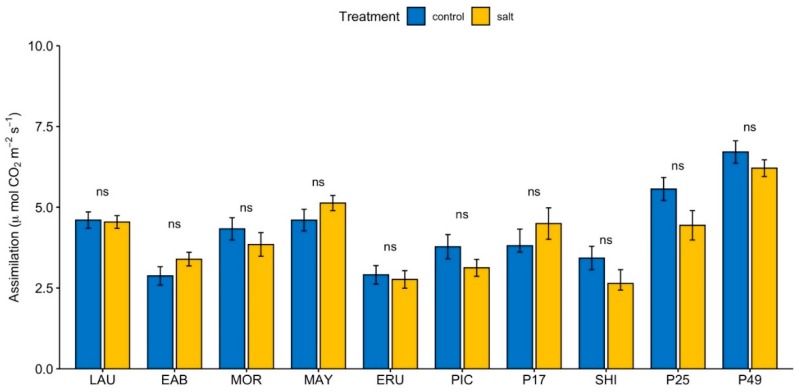
Effect of salinity on photosynthetic CO_2_ assimilation. Measurements from three intermediate-aged leaves from each plant were averaged for one reading. Values are means of four independent experiments, each with at least four biological replicates per cultivar/accession per condition. Bars represent standard errors; asterisks indicate statistical significance as calculated by the *t*-test. * (*p* ≤ 0.05), ** (*p* ≤ 0.01), *** (*p* ≤ 0.001), **** (*p* ≤ 0.0001), ns = not significant (*p* > 0.05).

In control conditions, CO_2_ assimilation in “Early Bird” and “Eruption” was significantly lower, whereas the CO_2_ assimilation in PI 253468 and PI 491154 was significantly greater than the overall mean (Supplemental Figure S6A). Under salinity, CO_2_ assimilation in “Early Bird”, “Eruption”, “Parris Island Cos”, and “Shining Star” was significantly lower, whereas the CO_2_ assimilation in “Mayfair” and PI 491154 was significantly higher than the overall mean (Supplemental Figure S6B). Photosynthetic rate in PI 491154 was significantly greater than most genotypes except PI 253468 under control conditions (Supplemental Figure S6C). Under salinity, PI 491154 had the greatest photosynthetic rate compared to all genotypes except “Mayfair” (Supplemental Figure S6D). We observed only minimal change in the photosynthetic CO_2_ assimilation between control and salinity treatments in all genotypes (Figure 3), indicating that changes in SPAD observed under salinity compared to control are independent from photosynthesis.

Vapor pressure deficit based on leaf temperature (VpdL) was significantly lower than the overall mean in “Mayfair”, and significantly higher in “Laura”, PI 171676a, and “Shining Star” under control conditions, suggesting more open stomates in “Mayfair” than “Laura”, PI 171676a, and “Shining Star”. Under salinity treatment, VpdL was significantly lower in “Mayfair” and “Eruption”, and significantly higher in “Shining Star” than in the overall mean. VpdL showed a small (16–20%) but statistically significant increase in “Early Bird”, “Morgana”, “Mayfair”, and “Parris Island Cos” under salinity treatment (Table 2), suggesting more closed stomates at the elevated salinity level. No significant difference between control and treatment was observed in “Laura”, “Eruption”, PI 171676a, “Shining Star”, PI 253468, and PI 491154, suggesting no significant effect of salinity on stomata opening.

No significant difference could be observed between salinity and control treatments for intercellular CO_2_ concentration (CI) for any cultivar (Supplemental Table S1). Stomatal conductance to H_2_O (Cond) decreased a significant 61% in “Morgana” in salinity compared to control and decreased a significant 77% in “Mayfair” under control compared to salinity. Cond decreased 43% and 44% in PI 253468 and PI491154, respectively, under salinity compared to control (Supplemental Table S2). Transpiration rate (Trmmol) similarly was reduced by a significant 72% in “Mayfair” under salinity compared to control (Supplemental Table S3).

### 3.5. Chlorophyll a Fluorescence

**Figure 4:**
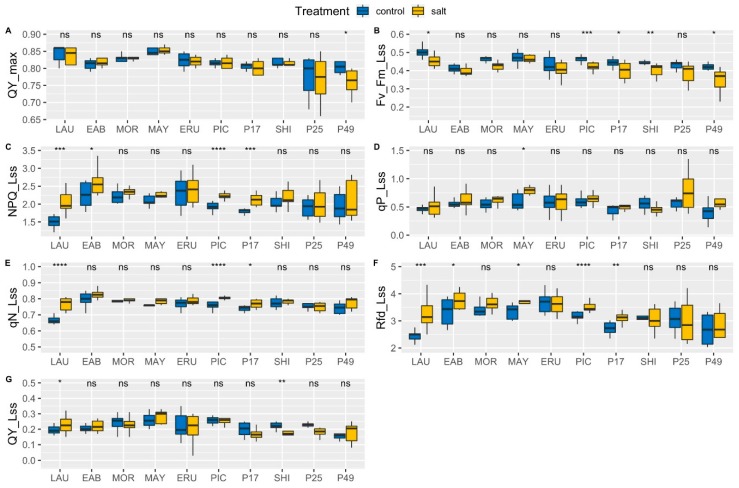
Analysis of chlorophyll fluorescence in salinity treated lettuce leaves. Chlorophyll fluorescence was measured using the PlantScreen phenotyping system and the FluorCam accessory. Values are means of two independent experiments, each with at least four biological replicates per cultivar/accession per condition. Fv/Fm or maximum quantum yield (QY_max) (**A**); Fv/Fm in steady state light (Fv_Fm_Lss) (**B**); non-photochemical quenching of maximum fluorescence in steady-state light (NPQ_Lss) (**C**); photochemical quenching in steady-state light (qP_Lss) (**D**); non-photochemical quenching of variable fluorescence in steady-state light (qN_Lss) (**E**); relative fluorescence decline in steady-state light (Rfd_Lss) (**F**); instantaneous photosystem II quantum yield in steady-state light (QY_Lss) (**G**). In above box plots, the boundary of the box closest to the x-axis indicates the 25th percentile, a black line within the box marks the median, and the boundary of the box farthest from the x-axis indicates the 75th percentile. Whiskers above and below the box indicate the 10th and 90th percentiles. Asterisks indicate statistical significance as calculated by the *t*-test. * (*p* ≤ 0.05), ** (*p* ≤ 0.01), *** (*p* ≤ 0.001), **** (*p* ≤ 0.0001), ns = not significant (*p* > 0.05).

Chlorophyll fluorescence was measured to determine plant performance under control or salinity conditions. The Kautsky effect [32] was measured in Pulse-Amplitude-Modulated (PAM) mode to make detailed measurements of the transitions from the dark-adapted state to the light-adapted state and back. This allowed measuring and calculating a range of photosynthetic parameters related to chlorophyll fluorescence, in addition to Fv/Fm (QY_max). There was no significant reduction in Fv/Fm_Lss in “Early Bird” in the same measurement, suggesting a more efficient photoprotective mechanism compared to “Laura”. Non-photochemical quenching of maximum fluorescence (NPQ) steadily increased from 0.22 (± 0.02) after the first light pulse (NPQ_L1), to 1.5 (± 0.05) in the light-adapted steady state (NPQ_Lss) in “Laura” under control conditions (Supplemental Data 2 and 5). In the condition of elevated salinity, NPQ_L1 increased from 0.30 (± 0.03) to 2.09 (± 0.11) (Supplemental Data 2 and 5). There was a significant, 39% increase in NPQ_Lss under salinity compared to control. In “Early Bird”, NPQ increased from 0.33 (± 0.03) at NPQ_L1, to 2.26 (± 0.10) at NPQ_Lss under control (Supplemental Data 2 and 5). Under salinity, “Early Bird” NPQ_L1 was 0.41 (± 0.05), increasing to 2.59 (± 0.10) (NPQ_Lss) (Supplemental Data 2 and 5). The increase in NPQ_Lss in “Early Bird” under salinity compared to control was 15%. Similar to NPQ, non-photochemical quenching of variable fluorescence (qN), another measure of non-photochemical quenching, also increased significantly in “Laura” in control compared to salinity conditions. Thus, it appears that more light energy is dissipated in the form of NPQ in “Laura” than “Early Bird”, suggesting more efficient photosynthesis in “Early Bird” than “Laura”, and increased stress experienced by “Laura” under salinity compared to “Early Bird”. Level of photochemical quenching of Photosystem II (qP) indicates the proportion of open PSII reaction centers. In “Laura”, qP_Lss was 0.45 (± 0.03) in the control and 0.51 (± 0.05) under salinity, while in “Early Bird”, it was 0.53 (± 0.03) under the control and 0.68 (± 0.08) under salinity (Figure 4D).

There was a 13% and 28% increase in qP_Lss in “Laura” and “Early Bird”, respectively, indicating a higher number of open PSII reaction centers in “Early Bird” compared to “Laura” under both control and salinity conditions, and a higher number of open PSII reaction centers under salinity in both “Laura” and “Early Bird”. The ratio of fluorescence decline (Rfd) indicates photosynthetic rates, with higher ratios corresponding to higher quantum photochemical conversion [46]. Rfd gradually increased in “Laura” and “Early Bird” over the duration of the Kautsky curve after each saturating light flash, in both control and salinity conditions (Supplemental Data 2). Rfd in steady-state light (Rfd_Lss) was 2.42 (±0.07) in “Laura” under the control, compared to 3.27 (±0.16) under salinity (Figure 4F). In “Early Bird”, Rfd_Lss was 3.35 (±0.14) and 3.76 (±0.10) under control and salinity conditions respectively. Thus, Rfd significantly increased 35% and 12%, respectively, in “Laura” and “Early Bird”, in salinity compared to the control, also suggesting increased photosynthesis in both cultivars under salt treatment, particularly in “Laura”. However, along with photochemical processes, Rfd is also influenced by non-photochemical processes, so in this case, the increase in Rfd in “Laura” and “Early Bird” may likely be because of increase in NPQ contributing to Rfd than increase in photochemical conversion alone. This is supported by measurements of actual photosynthetic CO_2_ assimilation using the LiCor 6400 XT Portable Photosynthesis System, which suggest no significant change under salinity compared to control (Figure 3).

No significant difference was observed in any parameter between salinity and control in “Morgana” and “Mayfair”, except Rfd_Lss. Rfd_Lss in “Mayfair” indicated a significant, 14% increase under salinity compared to the control (Figure 4F). No significant increase could be observed in “Morgana”. Since NPQ was similar under salinity and control in “Mayfair”, it can be assumed that NPQ had minimal to no contribution to Rfd_Lss. Photosynthetic CO_2_ assimilation was observed to increase 10% under salinity compared to control in “Morgana” (Figure 3). Taken together, the data suggests an increase in photosynthesis in “Mayfair” compared to “Morgana” under salinity compared to control respectively.

When comparing “Eruption” with “Parris Island Cos”, 10% and 8% reduction in Fv/Fm_Lss was observed, respectively, in salinity compared to control. This suggests the plants may have been experiencing partial photoinhibition. Reduction in Fv/Fm_Lss in “Parris Island Cos” but not “Eruption”, was statistically significant, suggesting a more robust photoprotective mechanism in “Eruption” than in “Parris Island Cos”. Similarly, NPQ levels were significantly higher in “Parris Island Cos” under salinity compared to control, but not in “Eruption”. NPQ_Lss increased 17% in “Parris Island Cos” under salinity compared to the control, from 1.92 (±0.04) to 2.26 (±0.04). qN also similarly increased significantly in “Parris Island Cos”, from 0.76 (±0.01) to 0.80 (±0.004) (Figure 4E). Together, these data suggest a higher amount of energy dissipated as heat energy in “Parris Island Cos” than in “Eruption”, supporting the observation that photosynthesis is more efficient in “Eruption” than in “Parris Island Cos” under salinity conditions. Rfd_Lss in “Parris Island Cos” in control was 3.17 (±0.05) compared to 3.50 (±0.05) under salinity, leading to a 10% increase. However, considerable contribution to Rfd_Lss from NPQ_Lss can be assumed. Higher Rfd suggests an increase in the rate of photosynthesis in “Parris Island Cos” under salinity compared to control.

An approximately 10% reduction in Fv/Fm_Lss, from 0.44 (±0.01) to 0.40 (±0.02) in PI 171676a and from 0.45 (±0.003) to 0.41 (±0.01) in “Shining Star” was observed. NPQ_Lss levels were significantly higher in PI 171676a under salinity compared to control, rising from 1.80 (±0.03) to 2.12 (±0.06) while remaining largely unchanged in “Shining Star”. Similarly, qN_Lss levels had a small but significant increase, from 0.74 (±0.01) to 0.77 (±0.01), while remaining largely unchanged in “Shining Star”. Rfd_Lss levels increased significantly, 15% in PI171676a under salinity compared to the control, from 2.72 (±0.09) to 3.12 (±0.07), suggesting a reduction in photosynthesis. “Shining Star” did not show any significant change in Rfd_Lss between treatment, indicating no effect of elevated salinity on photosynthesis.

In PI 253468, all the major chlorophyll fluorescence parameters remained unchanged in salinity as compared to the control (Figure 4). In PI 491154, Fv/Fm showed a small but significant, 5% reduction under salinity, from 0.80 (±0.01) to 0.76 (±0.01). However, this lower Fv/Fm was still in the range normally observed in healthy plants. After repeat applications of actinic light flashes according to the Kautsky method, Fv/Fm steadily decreased, to a final 16% reduction in steady-state light (Fv/Fm_Lss) under salinity, from 0.41 (±0.01) to 0.35 (±0.02). In PI 491154, NPQ showed a significant increase at the beginning of the Kautsky curve, at the time of the initial light flashes, but reduced towards the end of the curve, showing no significant difference under salinity (Supplemental Data 2). qN followed a similar pattern, in which it increased significantly at the beginning of the Kautsky curve but gradually reduced towards the end. In PI 253468, qN showed no significant difference under salinity. Rfd gradually spiked with each flash of actinic light under the Kautsky method, suggesting an increased photosynthesis rate in salinity compared to the control (Supplemental Data 2). However, Rfd leveled off in steady-state light (Rfd_Lss) to indicate a slight reduction in photosynthesis under salinity. This is consistent with reduction in Photosynthetic CO_2_ assimilation under salinity in PI 253468 (Figure 3). These results suggest that, compared to PI 491154, PI 253468 had a more robust photosynthetic machinery under both tested conditions.

### 3.6. Principal Component Analysis

**Figure 5:**
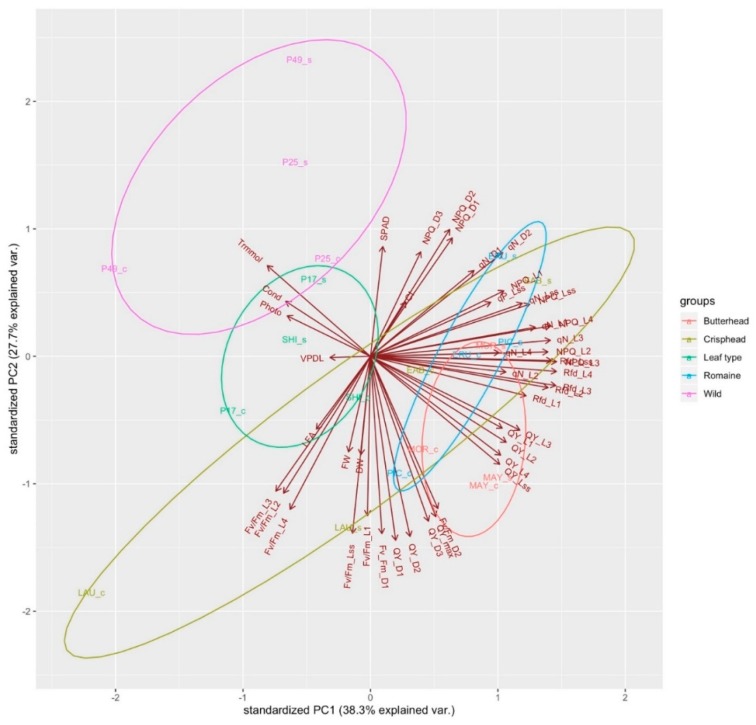
Principal component loading plot and scores of PCA on morphological, physiological, and chlorophyll fluorescence parameters of butterhead, crisphead, leaf-type, romaine, and wild lettuce grown in control and salinity conditions and grouped by colored ellipses around each lettuce type.

PCA was conducted on all the physiological, morphological, and chlorophyll fluorescence variables together with all the treatments and genotypes (Figure 5). PC1 and PC2 account for 66% of the variance. Wild, leaf type, butterhead, and romaine lettuce form their own distinct clusters, whereas crisphead is more centrally distributed. Most of the variation in wild lettuce can be explained by high values of photosynthesis, stomatal conductance, and leaf transpiration, while most of the variation in leaf type can be explained by high values of photosynthesis, stomatal conductance, leaf transpiration, and low values of VpdL, Fv/Fm_L2, L3, L4, and total leaf area (Figure 5).

Most of the variation in Butterhead can be explained by lower values of QY_L1, QY_L2, QY_L3, QY_L4, QY_Lss, qN_L2, qN_L4, Rfd_L1, Rfd_L2, Rfd_L3, and Rfd_L4. 

**Figure 6:**
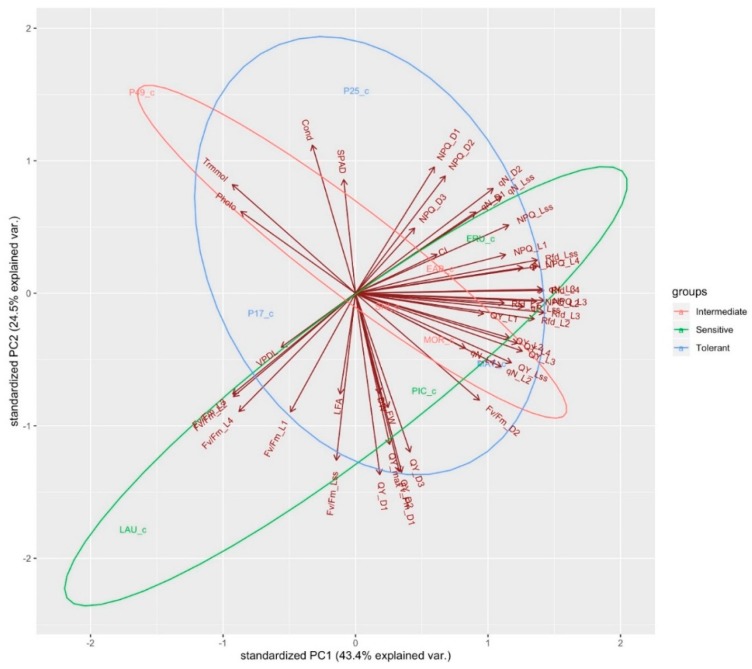
Principal component loading plot and scores of PCA on morphological, physiological, and chlorophyll fluorescence parameters of sensitive, intermediate, and tolerant genotypes grown under control conditions, grouped by colored ellipses.

**Figure 7:**
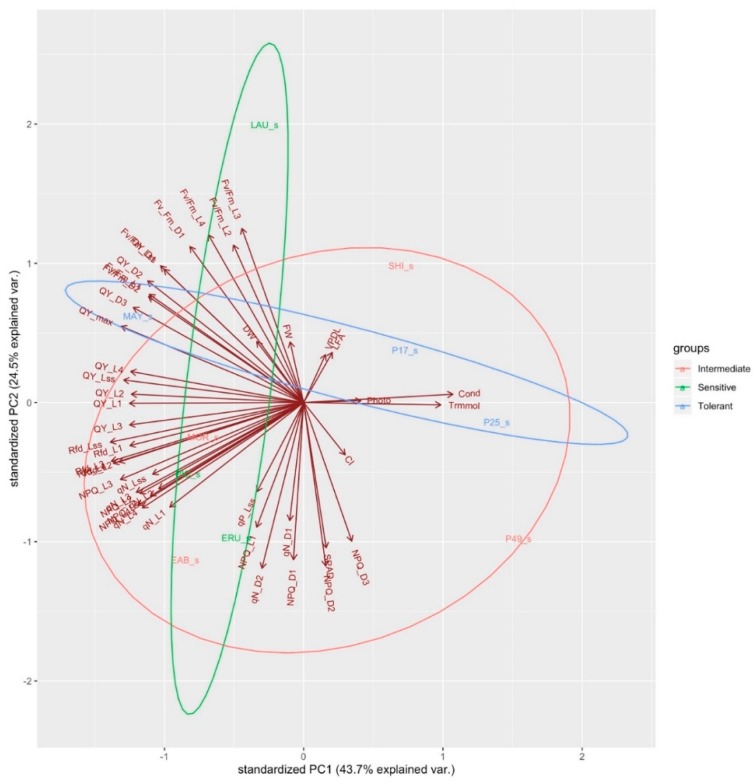
Principal component loading plot and scores of PCA on morphological, physiological and chlorophyll fluorescence parameters of sensitive, intermediate and tolerant genotypes grown under salinity conditions, grouped by colored ellipses.

Wild lettuce cultivars (PI 253468, PI 491154) cluster together, characterized by high values for SPAD, stomatal conductance, leaf transpiration, and photosynthesis. PI 171676a and “Laura” cluster together, characterized by low values of VpdL, leaf area, Fv/Fm_L1, Fv/Fm_L2, Fv/Fm_L3, Fv/Fm_L4 and, Fv/Fm_Lss. “Eruption” and “Early Bird” cluster together, characterized by high values of CI, NPQ_D1, NPQ_D2, NPQ_D3, NPQ_L1, NPQ_Lss, Rfd_Lss, qN_D1, qN_D2, and qN_Lss. “Shining Star”, “Morgana”, “Mayfair” and “Parris Island Cos” cluster together, characterized by high values of QY_L1, QY_L2, QY_L3, QY_L4, QY_Lss, Rfd_L1, Rfd_L2, qN_L2, Fv/Fm_L2, FW, DW, QY_D1, D2, D3, QY_max, and Fv/Fm_D1 (Figure 5). When a PCA was conducted on all the variables in control conditions, PC1, PC2, and PC3 together account for 80% of all variance (Figure 6). A PCA conducted under salinity conditions indicates PC1, PC2, and PC3 account for 80% of the variance (Figure 7). Sensitive and tolerant genotypes form tight distinct clusters while intermediate genotypes are more broadly distributed (Figure 7). Sensitive genotypes are characterized by low values of NPQ_L1, NPQ_L2, NPQ_D1, qN_D1, qN_D2, qP_Lss, qN_L1, qN_L4, QY_L3, Rfd_Lss, etc. Tolerant genotypes are characterized by high values of photosynthesis, leaf transpiration, stomatal conductance, VpdL, total leaf area, FW, DW, QY_max, QY_D1, QY_D2, QY_D3, Fv/Fm_D1, Fv/Fm_ D2, Fv/Fm_L2, Fv/Fm_L3, and Fv/Fm_L4 (Figure 7).

### 3.7. Correlation Analysis

**Figure 8:**
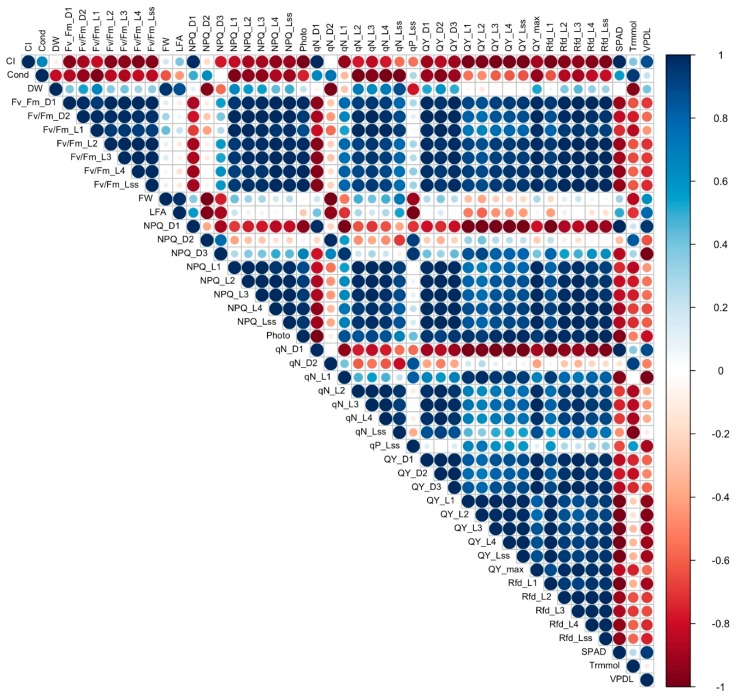
Graphical representation of a correlation matrix of morphological, physiological, and chlorophyll fluorescence parameters between tolerant genotypes PI 253468, PI 171676a, and “Mayfair”, highlighting the most correlated variables. Blue color represents positive correlation whereas red represents negative correlation. Color intensity and size of the circle are proportional to the correlation coefficients which are depicted in the legend to the right.

**Figure 9:**
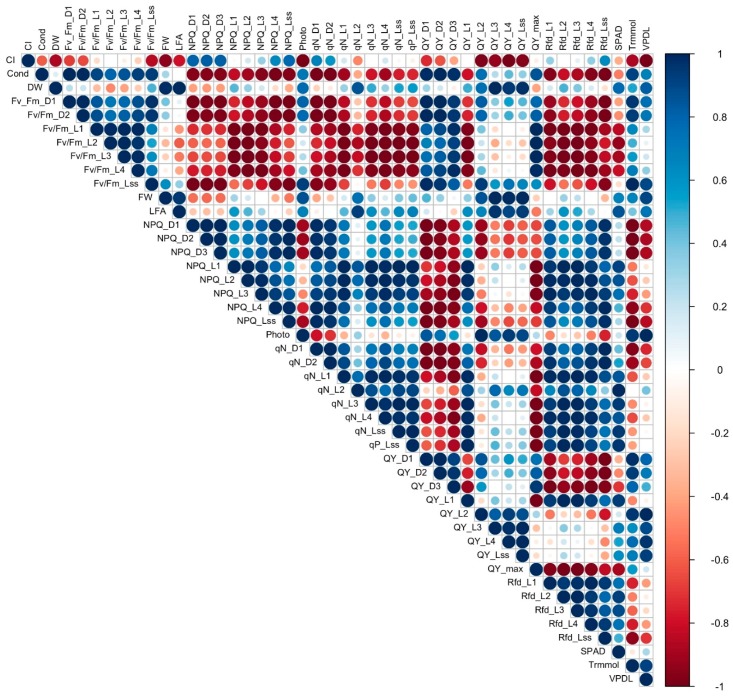
Graphical representation of a correlation matrix of morphological, physiological, and chlorophyll fluorescence parameters between sensitive cultivars “Laura”, “Eruption”, and “Parris Island Cos”, highlighting the most correlated variables. Blue color represents positive correlation whereas red represents negative correlation. Color intensity and size of the circle are proportional to the correlation coefficients which are depicted in the legend to the right.

To establish a starting point for understanding the complex relationship between the different measured variables, correlation analysis was conducted to determine the association and strength of association between all the variables in cultivars with a 16% to 31% reduction in FW under salinity (tolerant, Figure 8) and cultivars with a 60% to 67% reduction of FW under salinity (sensitive, Figure 9). Contrasting differences were observed in correlation of the variables between sensitive and tolerant cultivars. In tolerant cultivars, Fv/Fm values strongly positively correlated with most other chlorophyll fluorescence values, including NPQ, qN, QY and Rfd, while in sensitive cultivars they showed strong negative correlation with NPQ, qN and Rfd. Stomatal conductance in tolerant cultivars strongly negatively correlated with Fv/Fm values, DW, FW, LFA, and photosynthesis but showed strong positive correlation in sensitive cultivars. Photosynthesis showed strong positive correlation with qN, QY, and Rfd values, and a negative correlation with SPAD, leaf transpiration, and VpdL in tolerant cultivars, while in sensitive cultivars it did not have a strong correlation with qN, showed a weak negative correlation with Rfd, and a strong positive correlation with leaf transpiration and VpdL. QY_max showed a strong positive correlation with Rfd values, a strong negative correlation with SPAD, leaf transpiration, and VpdL in tolerant cultivars. On the other hand, QY_max showed a strong negative correlation with Rfd in sensitive cultivars, and a weak positive correlation with leaf transpiration. FW, DW, and LFA showed a strong negative correlation with leaf transpiration in tolerant cultivars but strongly positively correlated with leaf transpiration in sensitive cultivars. SPAD mostly showed a strong negative correlation with NPQ, qN, QY, and Rfd in tolerant cultivars but showed a strong positive correlation with the same values in sensitive cultivars.

## 4. Discussion

Effects of salinity on plants are well studied [19,20,21,22,23,24,25,26,70]. Salinity causes reduced turgor pressure and water uptake, leading to closure of stomata, reduced photosynthesis and reduced mineral uptake. Enzymes are sensitive to high Na^+^ concentrations, thus high accumulation of Na^+^ ions in the cytoplasm creates a toxic environment, and affects plant metabolism, increasing stress. Plants have evolved mechanisms to counter high salinity in order to survive. There are three main types of responses or tolerance to salinity observed in plants. (1) Tolerance to osmotic stress due to rapid shutdown of growth. In this rapid response to salinity, osmotic stress causes immediate reduction of cell expansion in root tips and young leaves, and stomatal closure. Tolerant plants have a more subdued response to osmotic stress, and are thus able to maintain growth and increased leaf area compared to sensitive ones, provided water supply is not an issue [71]. (2) Exclusion of Na^+^ ions from leaf blades. This process is slower, and takes place over many days or weeks [19,25]. Roots of tolerant plants are able to exclude Na^+^ from the surrounding medium, thus limiting the accumulation of Na^+^ in the leaves. Sensitive plants, which are unable to do so, exhibit toxic effects of Na^+^ accumulation. Depending on the species, it can take anywhere from days to weeks to observe this effect. (3) Tissue tolerance, the mechanism in which tolerant plants can sequester Na^+^ and Cl^−^ away from the cytoplasm. The ions are most commonly repossessed into cell wall or vacuoles, thus delaying and/or reducing their toxicity effect [25,72]. Older leaves tend to accumulate higher levels of Na^+^ than younger ones and are retained longer on the tolerant plant compared to sensitive one. Salt-tolerant plants or halophytes, have evolved efficient and effective tolerance mechanisms, and can survive in the presence of extreme salt concentrations, from 300–500 mM [73,74]. Euhalophytes, a sub-classification of halophytes, can tolerate even greater concentrations of salt, and do so by various mechanisms, including salt exclusion, salt elimination, salt succulence, and salt redistribution. These mechanisms work by preventing entry of salt into the vascular system, actively secreting salt via specialized glands or hair, increasing the storage volume of cells to accommodate more water along with increased salt, or redistributing Na^+^ and Cl^−^ ions away from actively transpiring leaves (where these concentrations can be higher), to other parts of the plant, by translocating via the phloem. The end result of all these mechanisms is that salt concentration is kept reasonably low and constant for extended periods of time. Another mechanism by which euhalophytes tolerate extremely high salt concentrations is by accumulation of soluble carbohydrates in their cell sap, which reduces the osmotic potential relative to the soil solution [19]. Most crop plants, including lettuce, are glycophytes, which cannot survive under salt concentrations greater than 100–200 mM, and lack the specialized mechanisms and structures present in euhalophytes for surviving in extreme salt concentrations. Regardless of inability or ability of glycophytes and halophytes to survive under high salt conditions, neither can tolerate high salt concentrations in their cytoplasm. Based on our observations at the end of the 4-week growth period, it appears that lettuce mainly exhibits osmotic stress response, resulting in reduced growth and leaf area. Tolerant lettuce cultivars appear to have a less severe osmotic stress response, because of which they exhibit a relatively smaller reduction in size and leaf area compared to control. Under salinity (or control), none of the lettuce plants showed increased senescence or abscission of older leaves, thus it seems that lettuce does not show the “exclusion from leaves” response to salinity during the four weeks of salt stress. It is possible that lettuce exhibits this response but can only be observed after a longer duration of salt stress and/or older stage of lettuce.

Photosynthetic performance is a key aspect of how plants adapt to their growth conditions. Most stresses, including salinity stress, affect photosynthetic activity, and chlorophyll fluorescence can be used as a reliable, rapid and non-invasive indicator to analyze photosynthetic function [19,75]. Salinity affects photosynthetic activity, both as a result of toxicity from Na^+^ ion accumulation in the leaves over time, and also as a response mechanism by the plant to deal with high salt stress. The plant needs to optimally regulate the light energy absorbed, so as to limit the photooxidative damage resulting from reduced photosynthesis, and maintain plant growth. Excess light energy that the plants absorb, if unquenched by photoprotective mechanisms or photochemistry, can lead to formation of the excited state of chlorophyll (triplet chlorophyll) in the photosystems, which can react with molecular oxygen to produce highly damaging reactive oxygen species (ROS) singlet oxygen (^1^O_2_) or superoxide (O_2_^•−^) [76,77]. If ^1^O_2_ or O_2_^•−^ come into contact with water, they can react to produce the equally damaging ROS hydrogen peroxide (H_2_O_2_). ROS can cause damage to other cellular components, including photosystem I and photosystem II. Scavenging ROS molecules requires a lot of energy, and is counter-productive to the already stressed plant trying to maintain growth. In addition, ROS can activate genetic pathways leading to cell death [78,79], leading to premature senescence and death. Thus, to maintain photosynthesis and growth and avoid cellular damage, plants use short-term and long-term acclimation to changes in their environment. This includes minimizing the light absorbed and diverting the energy from photosynthesis to photoprotective mechanisms. Chlorophyll fluorescence can then be used to quantify the effectiveness of photoprotective mechanisms. For example, Fv/Fm can be measured to quantify photoinhibition, and NPQ can be determined to quantify thermal dissipation. Under non-stressful conditions, light energy is captured by the photosynthetic pigments such as chlorophyll, in association with photosystem I and II and converted to chemical energy. Upon absorption of excess light, energy is dissipated as chlorophyll fluorescence or emitted as heat.

Automated and semi-automated phenotyping allows for fast and non-destructive measurements of leaf area and a wide range of chlorophyll fluorescence parameters, including Fv/Fm, NPQ, qN, and qP, that report the condition of the photosystems [18,35,75,80,81,82]. In addition, use of the phenotyping platform enabled us to measure chlorophyll fluorescence parameters for the entire plant (Supplemental Figures S1D and S1E, and Supplemental Data 5), thus yielding more accurate results than can be obtained by hand-held meters that analyze only a small area of a leaf. Measuring only Fv/Fm can provide only part of the whole picture, suggesting normalcy of the photosynthetic apparatus, when in fact the plant can be experiencing photoinhibition and photooxidative stress. Repeated application of saturating actinic light pulses under the Kautsky method leads to re-oxidation of the plastoquinone pool by Photosystem I due to transfer of electrons to the Calvin–Benson cycle. Fluorescence gradually declines to a much lower level, Fm_Lss in this light-adapted steady-state, and problems or inefficiencies related to the photosynthetic apparatus can become clear from these measurements. For example, in “Laura”, Fv/Fm was 0.84 under both control as well as salt-stress conditions, indicating healthy photosynthetic apparatus (Figure 4A). However, because of the Kautsky method, it was possible to observe a small but significant, 9% reduction in Fv/Fm_Lss in “Laura” under salinity compared to control.

As has been reported before, FW was more sensitive to salinity than DW [16,17,54]. Salinity caused significant reduction in water content in all genotypes except “Laura” and PI 491154 (Table 1), as indicated by DW/FW ratio, and an increased dry biomass compared to control (Table 1). Calculated leaf thickness was observed to either not change significantly (“Laura”, “Shining Star”, PI 253468, and PI 491154), or display a minor but significant 19–28% decrease in “Early Bird”, “Morgana”, “Mayfair”, “Eruption”, “Parris Island Cos”, and PI 171676a under salinity compared to control. To the best of our knowledge, only one other study [83] reported the effect of salinity on lettuce “leaf thickness” (specific leaf weight; SLW), which was calculated using a different formula (SLW = DW/LFA) than the one used in our study (FW/LFA). However, similar to our results, these authors observed no significant change in lettuce SLW under 600 ppm NaCl concentration in irrigation water when supplemented with nitrogen (NO_3_NH_4_ at 285 ppm). No significant change in SLW was also reported in arugula (*Eruca sativa* L.) [84] under 40 mM NaCl and 50% soil +25% sand +25% peat moss condition compared to control without NaCl (though SLW increased when using other two substrates). Thus, lettuce appears to exhibit morphological changes in leaves in response to salinity different from those observed in other species (*Phaseolus vulgaris*, *Gossypium hirsutum*, *Atriplex patula*, *Carrizo citrange*, *Cleopatra mandarin*, *Arbutus unedo*, *Myrtus communis*, *Eugenia myrtifolia*, *Viburnum tinus*, zucchini squash, tobacco, and tomato [19,85,86,87,88]), at least under the specific conditions used in our study. In many plant species, thickening of leaves under salinity is associated with tolerance, while no change in thickness or reduction in thickness of leaves is associated with sensitivity [19,85,88,89]. Because lettuce is highly sensitive to salinity [9], tolerance to salinity may not be associated with leaf thickening, at least at the growth stage and the conditions tested in this study. Studies reporting actual measurements of lettuce leaf thickness in salinity conditions are currently lacking in the literature. Future studies should focus on independently validating lettuce leaf thickness calculated using the FW/LFA formula [69] to the actual (measured) leaf thickness under a range of different conditions, growth stages and lettuce types to confirm strong correlation between calculated and measured leaf thickness observed in several other plant species [69].

Reduction in FW correlated with reduction in leaf area (Table 1 and Figure 1), suggesting that measurement of leaf area using automated or semi-automated devices may be a reliable way to non-destructively estimate trends in fresh weight, at least for lettuce grown under the conditions reported here.

Chlorophyll index values increased under elevated salinity in all eight accessions of cultivated lettuce while its values decreased in both accessions of *L. serriola* (Figure 2). Cultivated lettuce thus may have changes in pathways that upregulate chlorophyll production or downregulate its degradation under elevated salinity. Photosynthetic CO_2_ assimilation per leaf area was unchanged between salinity and control conditions in all the genotypes (Figure 3), similar to observations in other species [90]. Changes in cell anatomy as an adaptation response to salinity, resulting in smaller leaves and higher chloroplast density per unit leaf area [91,92,93,94], may be responsible for this phenomenon [25]. Increasing chlorophyll content is a mechanism used by some tolerant species in response to salinity to protect the photosynthetic process [19]. Since SPAD and photosynthesis were measured four weeks after application of salinity, the plants at this point were most likely acclimated to the effects of salinity such as reduction in leaf area, and regulated chlorophyll concentration to a higher level than control, in order to maintain photosynthetic rates similar to those in control conditions, as required by the plant.

Maintenance of similar photosynthetic rates in control and salinity conditions does not explain the reduction in FW and leaf area observed under elevated salinity. One explanation for this is that although photosynthetic rates are similar in both control and salinity, the smaller leaf area under salinity results in less net photochemical energy conversion per plant. Another hypothesis is that lettuce plants grown under elevated salinity spend considerable energy quenching photooxidative stress and limiting photooxidative damage, and thus, do not have enough energy to channel into new growth. Yet another possible explanation for reduced plant and leaf size is associated with the amount of absorbed CO_2_. Significantly higher VpdL under elevated salinity in “Early Bird”, “Morgana”, “Mayfair”, and “Parris Island Cos” suggests more closed stomata and potentially less absorbed CO_2_ for assimilation in these genotypes (Table 2). Consistently high VpdL can cause stunting or very slow growth [95], and may be one of the factors responsible for reduced growth in response to salinity. It is also possible that reduction in size may be due to the loss of turgor pressure due to salinity, which results in reduced cell elongation, cell division, slower leaf appearance, and smaller final size [23,92,93,96,97].

Based on a previous study [54], the most sensitive and the most tolerant genotypes were selected from several lettuce horticultural types. We hypothesized that the contrasting sensitivity and tolerance in the genotypes would allow studying the physiological changes and adaptations taking place in plants. Plants were grown in controlled conditions under continuous light to avoid daily and seasonal fluctuations in environmental conditions that can interfere with results of experiments. Constant cultivation conditions allow for more consistent measurements of photosynthetic rates and chlorophyll levels [98,99] regardless of time of the day, thus avoiding complications resulting from the effect of photoperiod [99]. Compared to a previous study [54], control plants in the present experiment reached approximately 4–5 times higher FWs, allowing for a better distinction between sensitive and tolerant accessions. Considerably large size of plants was likely caused by a combination of several factors, including cultivation under continuous light, optimal constant temperature, and protection from biotic and abiotic stresses.

Similar to our results, this previous study [54] reported no change in chlorophyll fluorescence parameters Fv/Fm. Their study was conducted using a handheld PAM fluorometer, and was only able to record limited data from one small area of the leaf. Using the PlantScreen system enabled us to record not only chlorophyll fluorescence from the entire plant, but also conduct in-depth analyses using the Kautsky method. This allowed recording more detailed and informative measurements of the state of the photosynthetic apparatus and detecting stress-related problems in the photosynthetic machinery.

Similar to a previous study [54], we also observed significant increase in chlorophyll index (SPAD) in the lettuce cultivars when grown under salinity compared to control conditions. However, this effect was confined in our experiment only to cultivated lettuce and not *L. serriola* accessions, which showed decrease in SPAD values. Because a decrease in the chlorophyll index in *L. serriola* was not reported in the previous study [54], it is possible that the response observed in our experiments may be caused by continuous light growing conditions, as some *L. serriola* accessions show substantial photoperiodism (Simko, unpublished results).

The PCA indicates the wild lettuce genotypes segregating in a cluster clearly separated from the other lettuce types. Wild lettuce, and to an extent leaf-type lettuce, are characterized more by physiological properties of leaf transpiration, stomatal conductance, photosynthesis, and SPAD than the chlorophyll fluorescence values, which tend to distinguish butterhead, romaine, and crisphead in separate clusters (Figure 5).

## 5. Conclusions

Based on fresh weight that is the main determinant of lettuce yield [6], our classification of genotypes differs from a previous study [54]. We show that “Laura”, “Eruption”, and “Parris Island Cos” are the most sensitive genotypes from this set, with a 60–67% reduction in FW under elevated salinity. The least sensitive (most tolerant) genotypes are “Mayfair”, PI 171676a, and PI 253468, whose FW under elevated salinity was reduced by 16–31%. The remaining four genotypes (“Early Bird”, “Morgana”, PI 491154, and “Shining Star”) showed an intermediate decrease (39–54%) in their FW. Phenomics facilitated conducting fast, non-destructive analyses of a large number of plants in a consistent and reproducible manner. Leaf transpiration showed a strong negative correlation with QY_max under salinity in tolerant cultivars, thus lower leaf transpiration may be important for maintaining vigor under salinity conditions. High values of stomatal conductance, leaf transpiration, VpdL, LFA, DW, FW, QY_max, Fv/Fm_L1, Fv/Fm_L2, Fv/Fm_L3, Fv/Fm_L4, Fv/Fm_D1, Fv/Fm_D2, QY_D1, QY_D2, and QY_D3 characterized the tolerant cultivars, whereas sensitive cultivars were characterized by low QY, qN, NPQ, and Rfd. Correlation analysis also indicated strong contrasting differences between these variables in sensitive and tolerant cultivars. Thus, for future selection of lines with higher tolerance to salinity conditions and minimal loss in yield, these traits can potentially be explored. Specific variables can also be used in future phenomics studies for rapidly screening breeding populations to identify lines with potentially higher tolerance to salinity. For example, higher total leaf area combined with higher Fv/Fm and QY can be used as a first screen to select tolerant lines, before moving on to invasive analyses. This study establishes the baseline physiological responses of selected tolerant and sensitive lettuce genotypes from each lettuce type under control and salinity conditions in highly controlled conditions of the growth chamber. The information can be used as a reference for future research aimed at studying differences at the cellular and molecular level.

## Figures and Tables

**Table 1:** Effect of salinity on fresh and dry weight of lettuce plants.

Cultivar	FW (g)			DW (g)			DW/FW	
	Control	Salt	% Reduction	Control	Salt	% Reduction	Control	Salt
LAU	9.9 ± 0.51 a	3.3 ± 0.39 b	67	1.47 ± 0.16 a	0.91 ± 0.21 b	39	0.078 ± 0.004 a	0.078 ± 0.009 a
EAB	13.2 ± 1.27 a	6.1 ± 0.64 b	54	1.93 ± 0.14 a	1.44 ± 0.20 a	25	0.079 ± 0.004 a	0.096 ± 0.003 b
MOR	11.4 ± 0.99 a	5.4 ± 0.65 b	52	1.72 ± 0.14 a	1.26 ± 0.18 a	27	0.079 ± 0.004 a	0.101 ± 0.007 b
MAY	7.2 ± 0.54 a	4.9 ± 0.86 b	31	1.94 ± 0.11 a	1.51 ± 0.10 b	22	0.080 ± 0.003 a	0.101 ± 0.003 b
ERU	6.4 ± 0.89 a	2.3 ± 0.38 b	65	1.01 ± 0.08 a	0.70 ± 0.13 a	31	0.078 ± 0.002 a	0.092 ± 0.002 b
PIC	10.7 ± 0.84 a	4.3 ± 0.40 b	60	1.93 ± 0.16 a	1.24 ± 0.19 b	36	0.095 ± 0.006 a	0.122 ± 0.005 b
P17	12.7 ± 1.87 a	9.2 ± 1.54 a	27	2.49 ± 0.28 a	1.91 ± 0.20 a	23	0.102 ± 0.004 a	0.116 ± 0.003 b
SHI	14.8 ± 1.57 a	9.0 ± 1.44 b	39	2.11 ± 0.22 a	1.72 ± 0.19 a	19	0.074 ± 0.004 a	0.103 ± 0.004 b
P25	2.4 ± 0.43 a	2.0 ± 0.38 a	16	0.76 ± 0.15 a	0.81 ± 0.20 a	−7	0.112 ± 0.006 a	0.145 ± 0.009 b
P49	3.8 ± 0.34 a	2.0 ± 0.20 b	47	1.19 ± 0.20 a	0.59 ± 0.12 b	50	0.139 ± 0.009 a	0.135 ± 0.007 a

Values are means of two independent experiments, each with at least four biological replicates. Error is represented by standard error. Means followed by different letters in each genotype and parameter indicate significant difference, with *p* ≤ 0.05, as calculated by the *t*-test.

**Table 2:** Vapor pressure deficit based on leaf (VpdL) in control and salinity treatments.

Cultivar	Treatment	VpdL (kPa)	% Reduction	Significance
LAU	Control	0.72 ± 0.03	0	ns
LAU	Salt	0.72 ± 0.02		
EAB	Control	0.67 ± 0.03	−17	*
EAB	Salt	0.79 ± 0.04		
MOR	control	0.58 ± 0.03	−18	***
MOR	Salt	0.69 ± 0.01		
MAY	control	0.53 ± 0.02	−16	**
MAY	Salt	0.62 ± 0.02		
ERU	control	0.61 ± 0.03	8	*
ERU	Salt	0.56 ± 0.01		
PIC	control	0.61 ± 0.03	−20	*
PIC	Salt	0.73 ± 0.04		
P17	control	0.82 ± 0.04	9	ns
P17	Salt	0.75 ± 0.03		
SHI	control	0.76 ± 0.03	−3	ns
SHI	Salt	0.78 ± 0.03		
P25	control	0.60 ± 0.04	−13	ns
P25	Salt	0.68 ± 0.02		
P49	control	0.59 ± 0.03	−12	ns
P49	Salt	0.66 ± 0.03		

Values are means of four independent experiments, each with at least four biological replicates per genotype per condition. Error is represented by standard error. Asterisks indicate statistical significance as calculated by the *t*-test. * (*p* ≤ 0.05), ** (*p* ≤ 0.01), *** (*p* ≤ 0.001), **** (*p* ≤ 0.0001), ns = not significant (*p* > 0.05).

## Supplementary Materials

The following are available online at https://www.mdpi.com/1424-8220/19/21/4814/s1, Supplemental Data 1: Chlorophyll fluorescence parameters symbols, formulae and description. Supplemental Data 2: *t*-test analysis, comparison of chlorophyll fluorescence parameters measured for lettuce plants grown in salinity (“salt”) or control conditions. Supplemental Data 3: ANOVA statistics of chlorophyll fluorescence parameters for lettuce plants grown in control conditions. Supplemental Data 4: ANOVA statistics of chlorophyll fluorescence parameters for lettuce plants grown in salinity conditions. Supplemental Data 5: Animations showing progression of NPQ, Rfd and Fv/Fm parameters in two pairs of lettuce cultivars with contrasting tolerance to salinity. Supplemental Figure S1: PlantScreen^TM^ Transect XZ system, data acquisition and analysis workflow, and sample images of plant morphology. Supplemental Figure S2: ANOM analysis of FW of lettuce plants grown in control (S2A) or salinity (S2B) conditions; ANOVA of FW of lettuce plants grown in control (S2C) or salinity (S2D) conditions. Supplemental Figure S3: ANOM analysis of DW of lettuce plants grown in control (S3A) or salinity (S3B) conditions; ANOVA of DW of lettuce plants grown in control (S3C) or salinity (S3D) conditions. Supplemental Figure S4: ANOM analysis of total leaf area of lettuce plants grown in control (S4A) or salinity (S4B) conditions; ANOVA of total leaf area of lettuce plants grown in control (S4C) or salinity (S4D) conditions. Supplemental Figure S5: ANOM analysis of SPAD in lettuce plants grown in control (S5A) or salinity (S5B) conditions; ANOVA of chlorophyll index of lettuce plants grown in control (S5C) or salinity (S5D) conditions. Supplemental Figure S6: ANOM analysis of Photosynthetic CO_2_ Assimilation in lettuce plants grown in control (S6A) or salinity (S6B) conditions; ANOVA of Photosynthetic CO_2_ Assimilation of lettuce plants grown in control (S6C) or salinity (S6D) conditions. Supplemental Table S1: Intercellular CO_2_ concentration (CI) in control and salinity treatments. Supplemental Table S2: Stomatal conductance to H_2_O (Cond) in control and salinity treatments. Supplemental Table S3: Transpiration rate (Trmmol) in control and salinity treatments. Supplemental Table S4: Calculated leaf thickness of aerial tissue in control and salinity treatments.
