# Supplementary material for: Phenomic and Physiological Analysis of Salinity Effects on Lettuce
Source: Sensors (Basel). 2019 Nov 5;19(21):4814. doi: 10.3390/s19214814 (PMC6864466; doi:10.3390/s19214814)
Supplement: Supplementary file 1 [file sensors-19-04814-s001.zip › Supplemental Figures and Tables.docx]

**Supplemental Figure S1A**


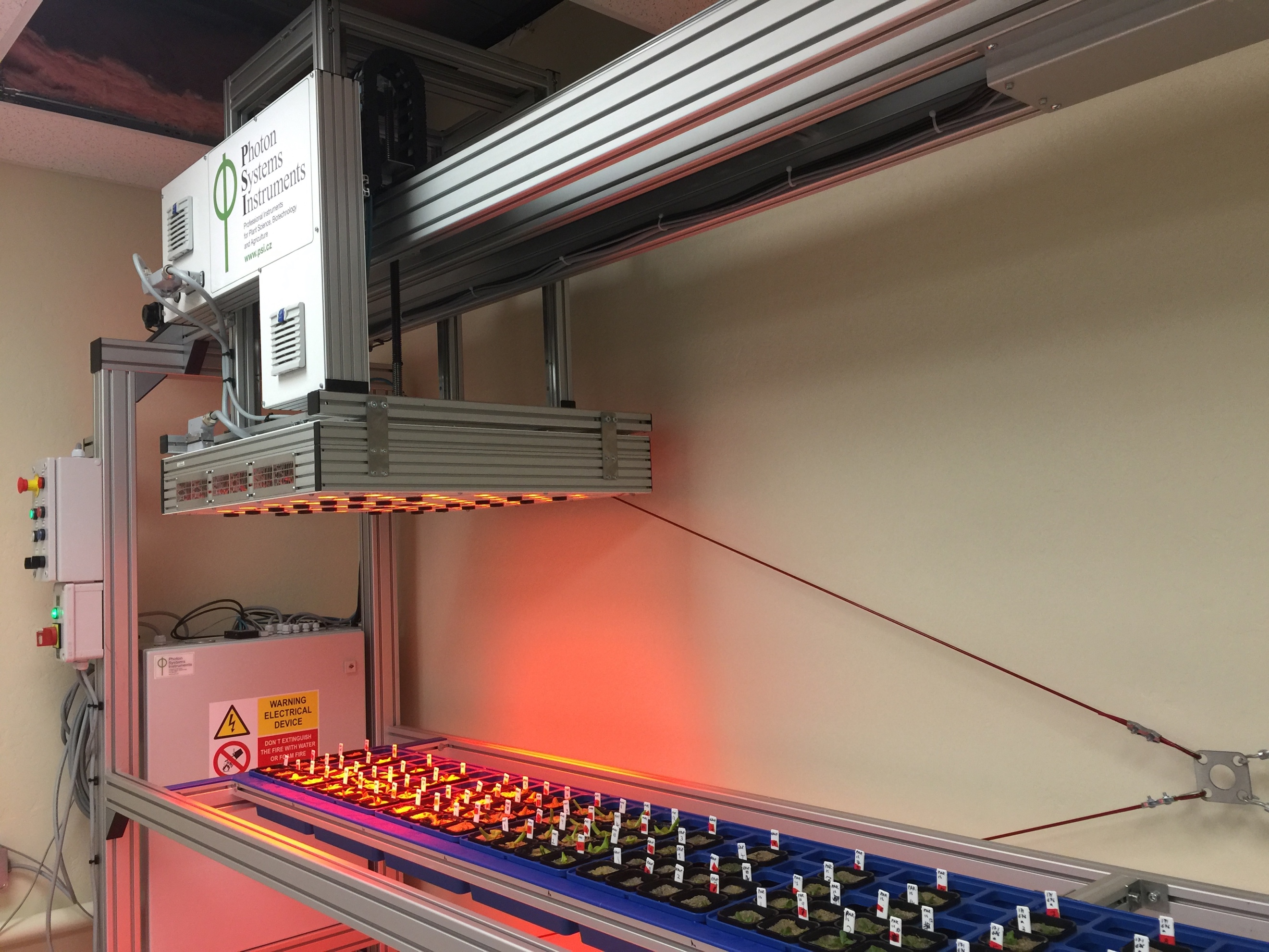


**Supplemental Figure S1B**

**
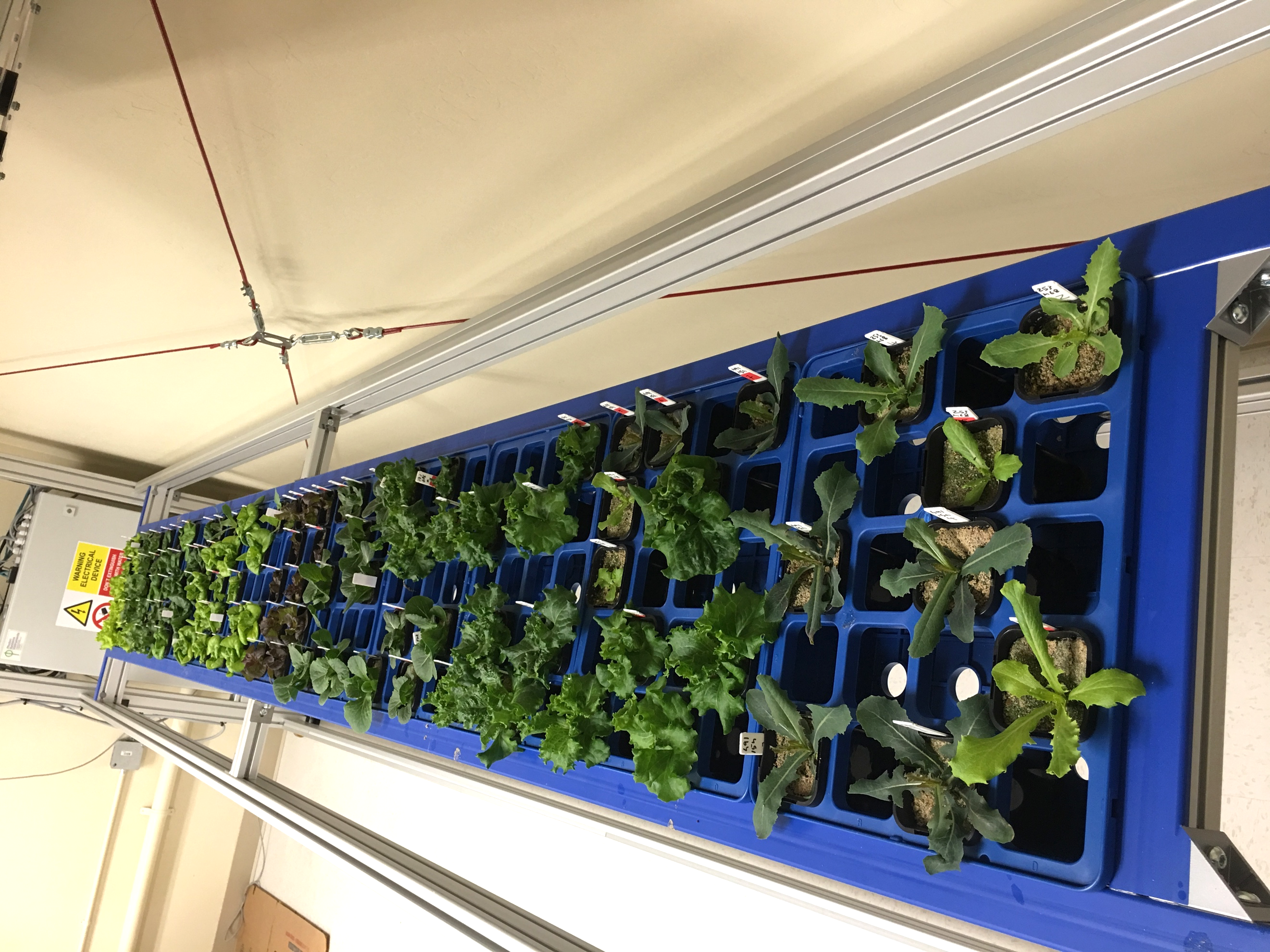
**
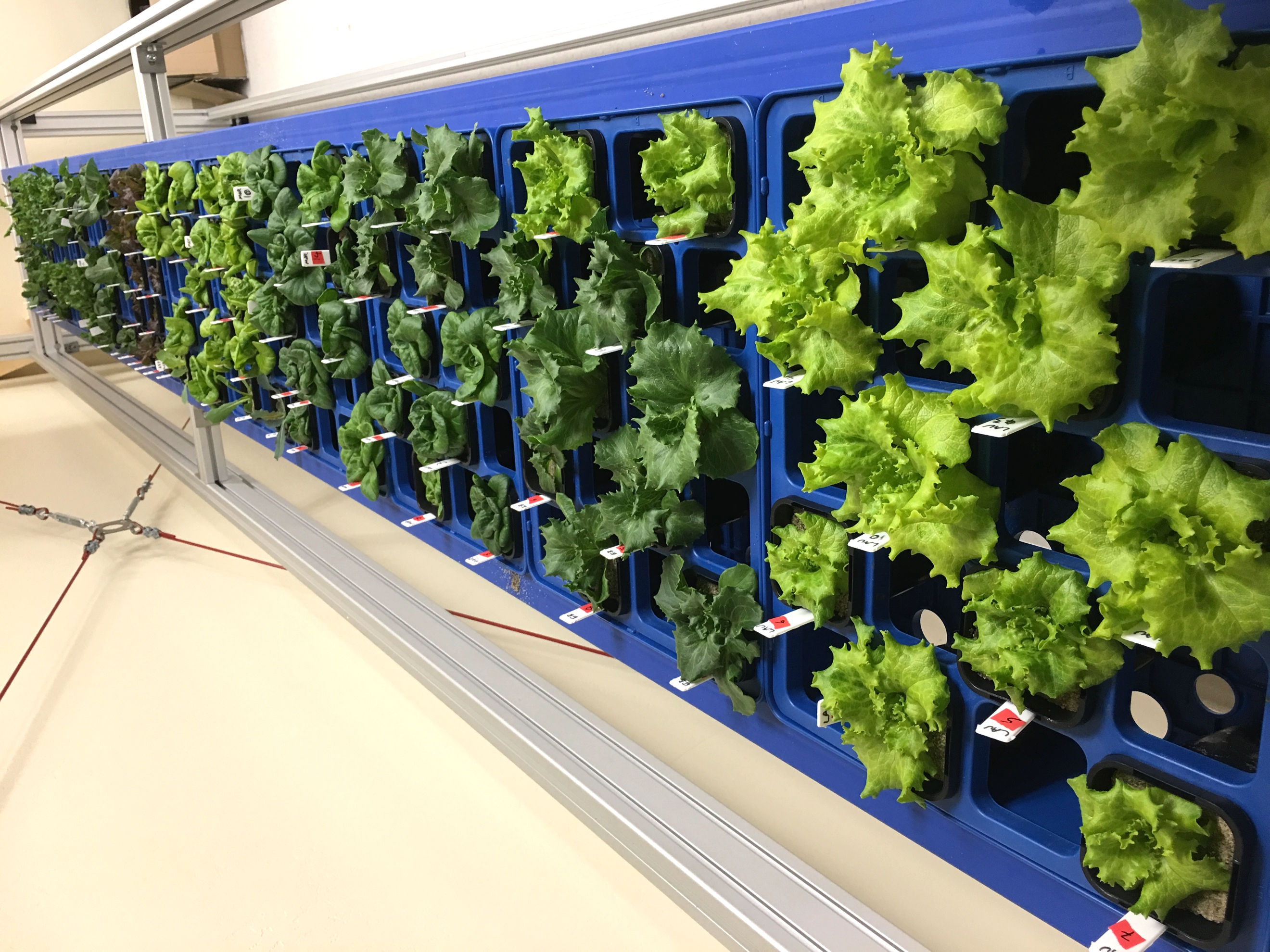


**Supplemental Figure S1C**


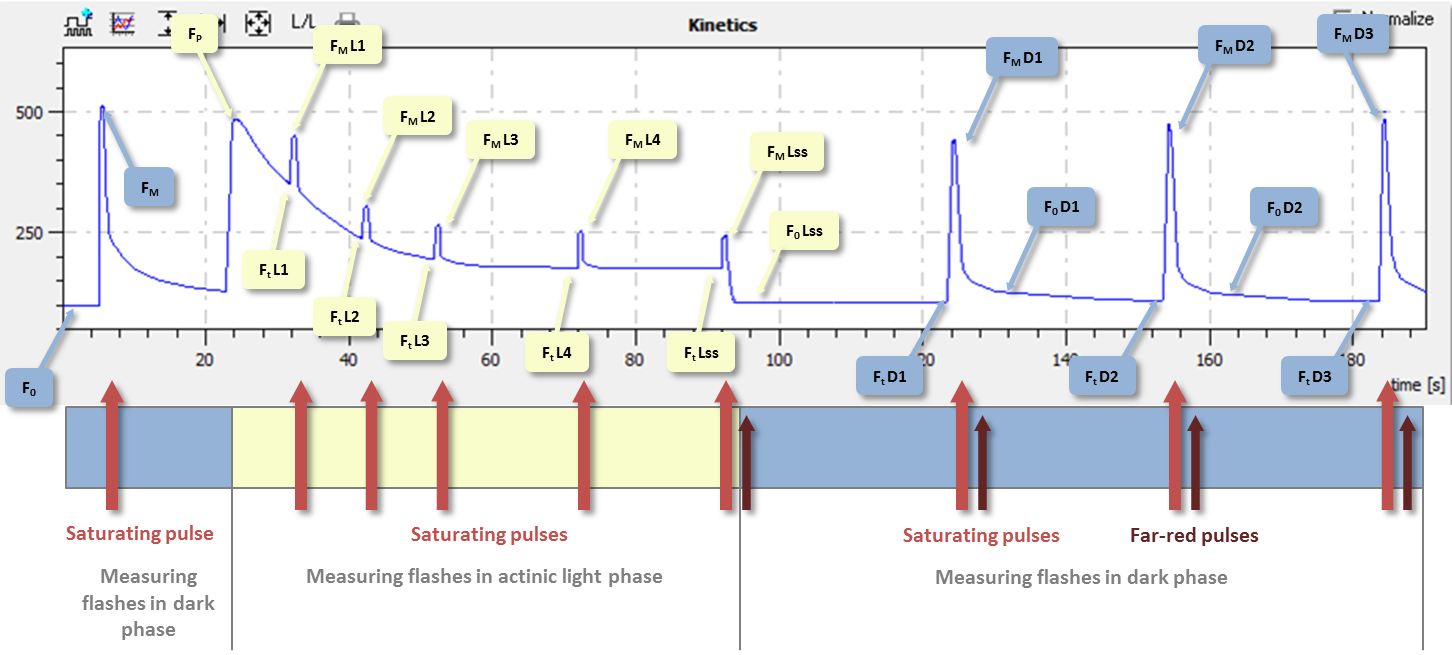


**Supplemental Figure S1D**


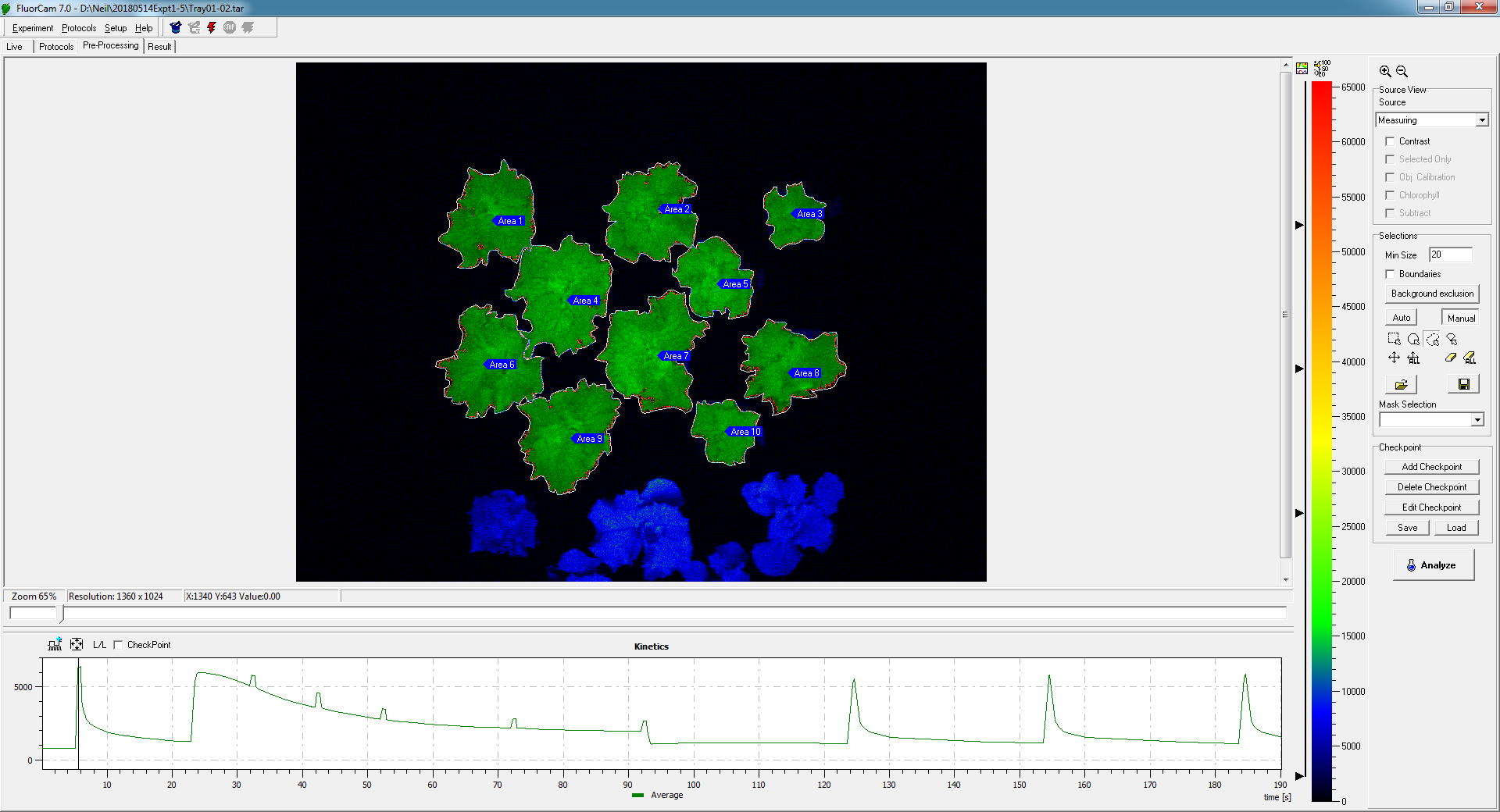


**Supplemental Figure S1E**


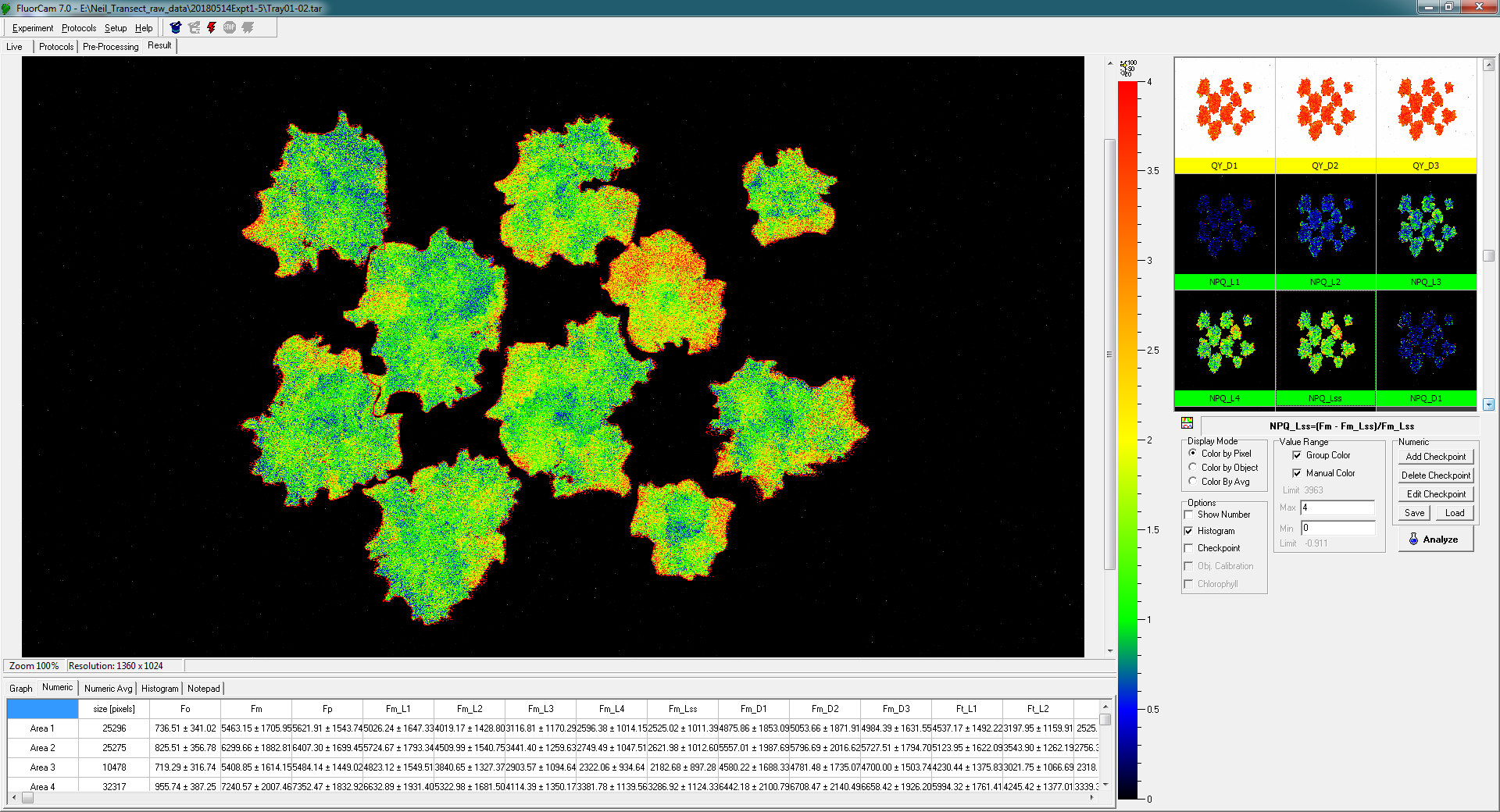


**Supplemental Figure S1F Supplemental Figure S1G**


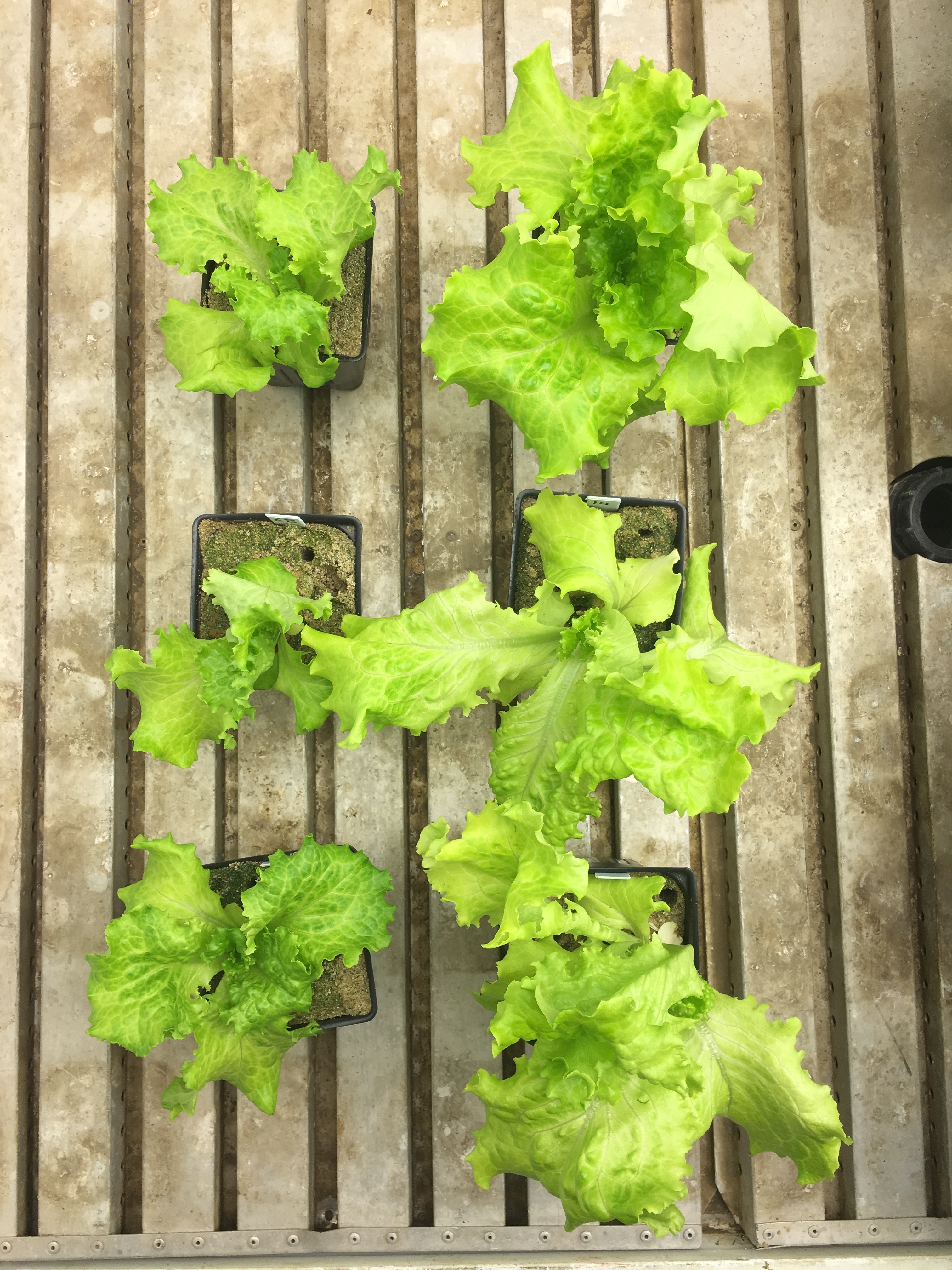

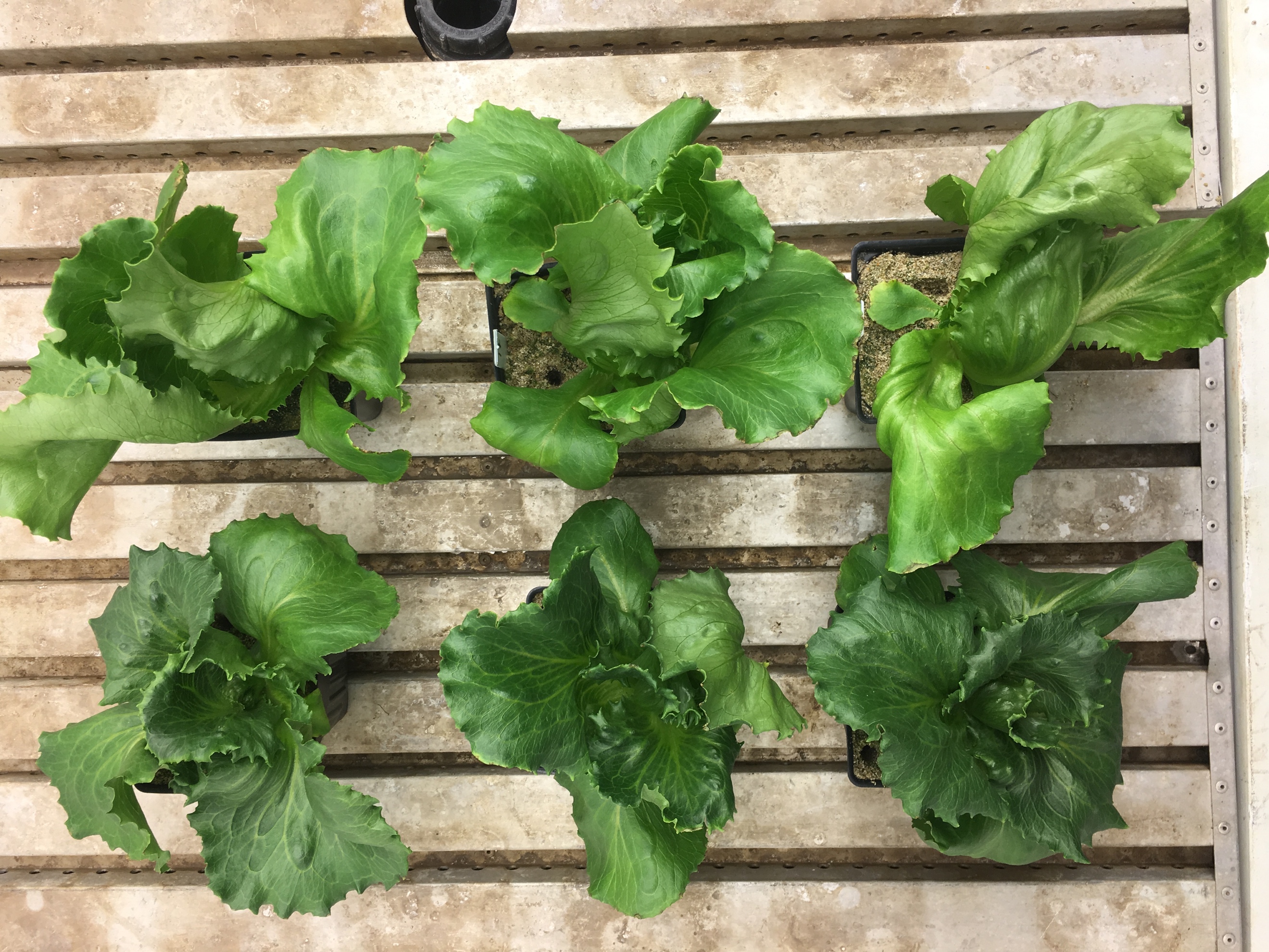


Laura (Crisphead type, sensitive). Early Bird (Crisphead type, tolerant).

Left, control, right, salt. Left, control, right, salt.

**Supplemental Figure S1H**


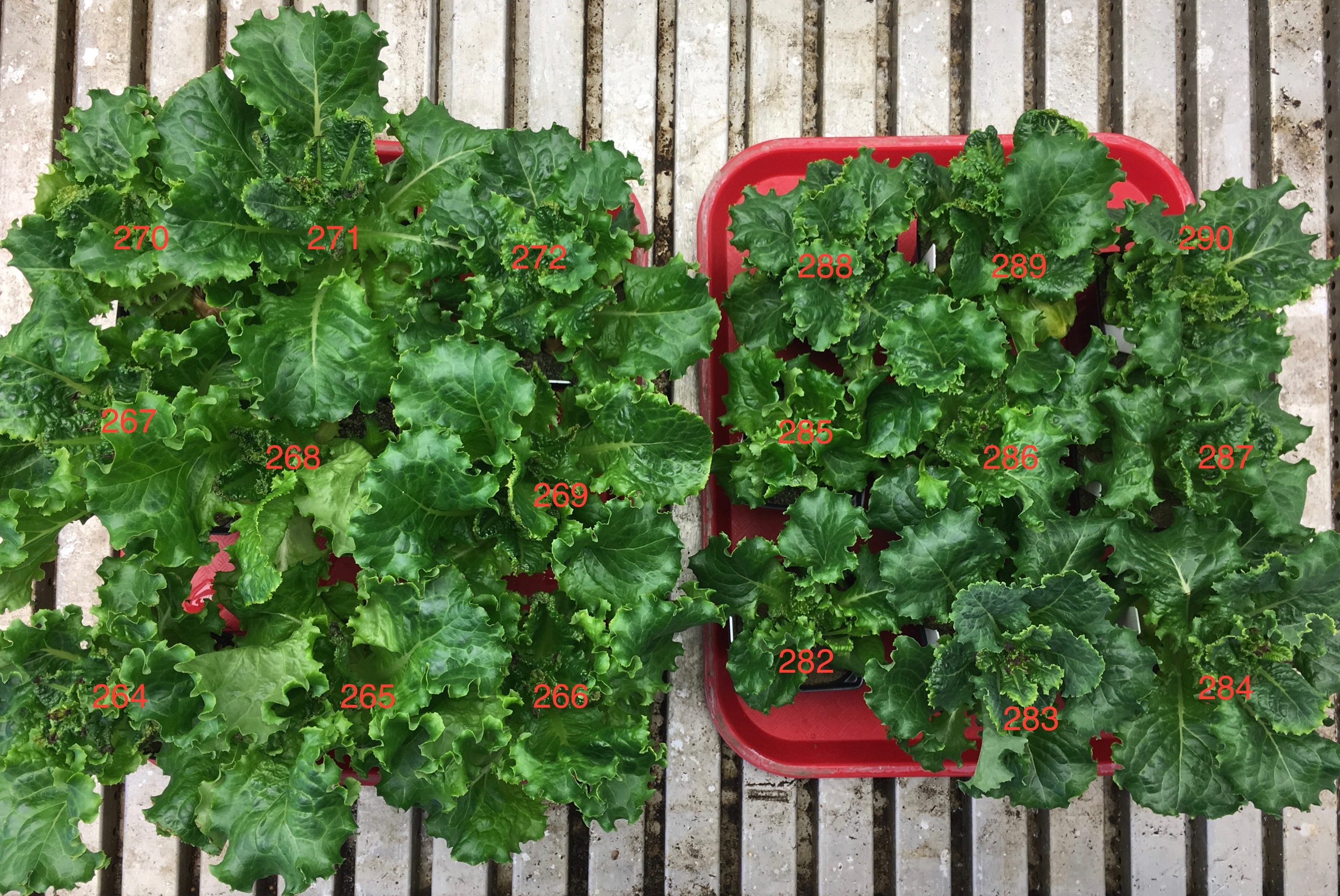


PI171676a (leaf-type, tolerant); control (left), salt (right).

**Supplemental Figure S1I**


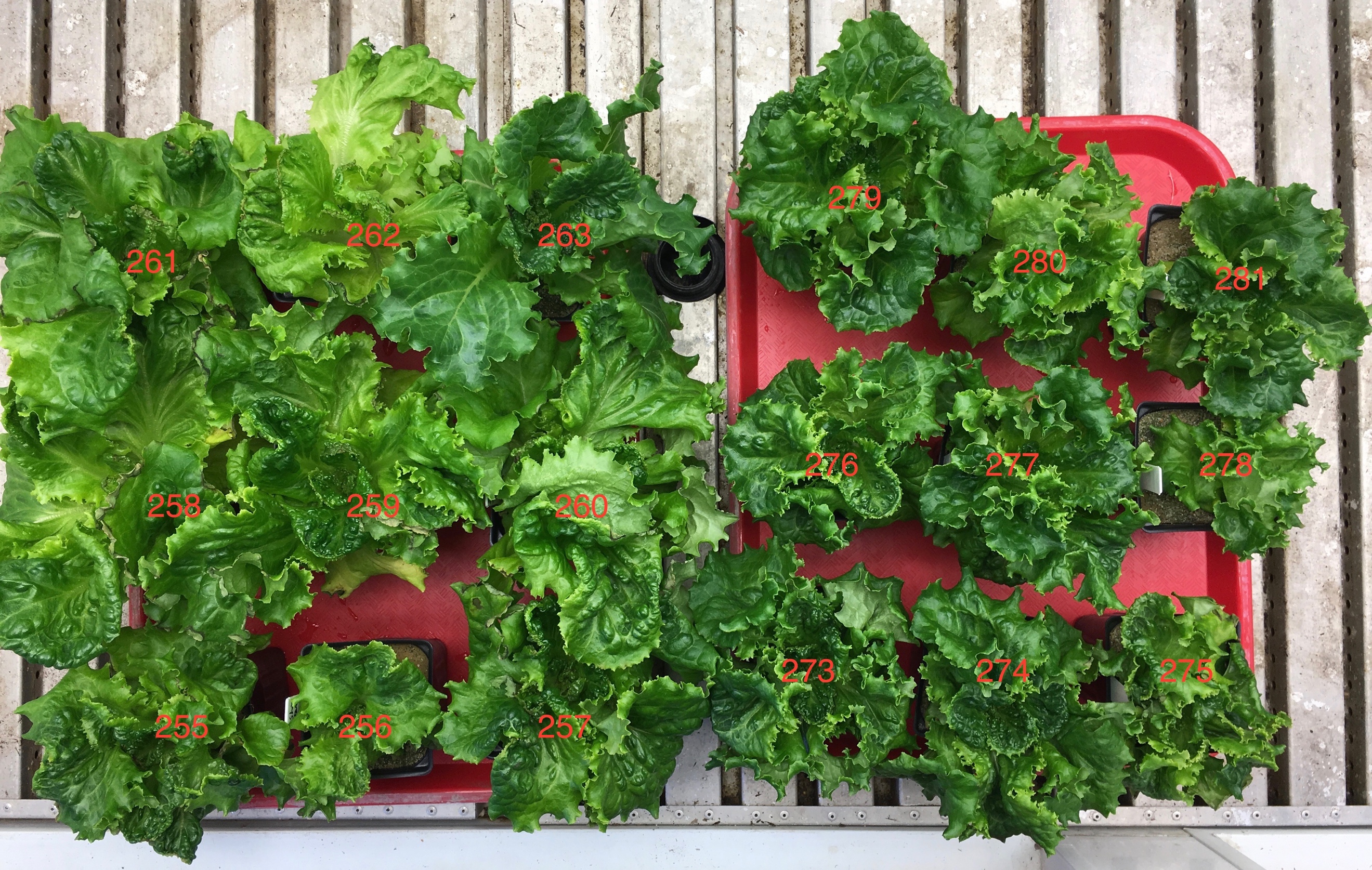


Shining Star (leaf-type, sensitive); control (left), salt (right).

**Supplemental Figure S1**. PlantScreen^TM^ Transect XZ system, data acquisition and analysis workflow, and sample images of plant morphology.

Robotic arm moves along the X-axis for up to 2 m. Height is adjustable up to 240 mm. Module at the end of the arm contains a LED light array consisting of actinic red-orange, cool-white, far-red and ultraviolet lamps, RGB and CCD cameras in the center (S1A). Up to 240, 10 cm pots in 12 trays, each with 20 slots, can be accommodated in this configuration. For bigger plants, the pots are spaced farther apart to prevent leaf overlap, or trays with bigger slots are used (S1B). Layout of the samples was programmed into the PlantScreen Scheduler software for automated acquisition of data. Appropriate height, light intensity and sensor sensitivity adjustments were made in order to prevent pixel overflow. “Quenching with far-red Actinic1” protocol within Fluorcam7 software contained the set of instructions to control operation of the camera and light sources to produce the light flashes for set duration at set intervals (S1C) and was used to measure the Kautsky effect in pulse amplitude modulated (PAM) mode.

Due to the different shapes and sizes of the various lettuce genotypes and additional size variation due to salinity treatment used in this study (S1F, S1G, S1H, S1I), it was difficult to automate application of plant masks. Thus, plant masks (green) were drawn manually in Fluorcam7 using the built-in lasso tool for accurate analysis of data, to ensure the entire plant was included in the measurements. Each plant mask got assigned a unique identifier (S1D). Background exclusion was applied and images analyzed automatically by Fluorcam7 based on the F_0_, Fm, Fp and Ft measurements, which were estimated by integrating values from each pixel across the entire leaf area. Background is represented by blue, and indicates the area not selected in the plant mask (S1E). After analysis, chlorophyll fluorescence data per plant was available as false color images (S1E) and quantitative numbers. False color images represent pixel-by-pixel values of the respective chlorophyll fluorescence parameter across the entire leaf area, thus allowing one to evaluate variations across the leaf surface between treatments and also between genotypes for each parameter. Processed numerical data was exported from Fluorcam7 as simple text files. Statistical analyses and visualization were performed in R.

Note: Plants seen in S1A are for illustration purposes only, and are not representative of the plant growth stage reported in this manuscript. S2C has been reproduced with permission from the FluorCam Instruction Manual V.2 (Photon Systems Instruments).

**Supplemental Figure S2A.**

(control)


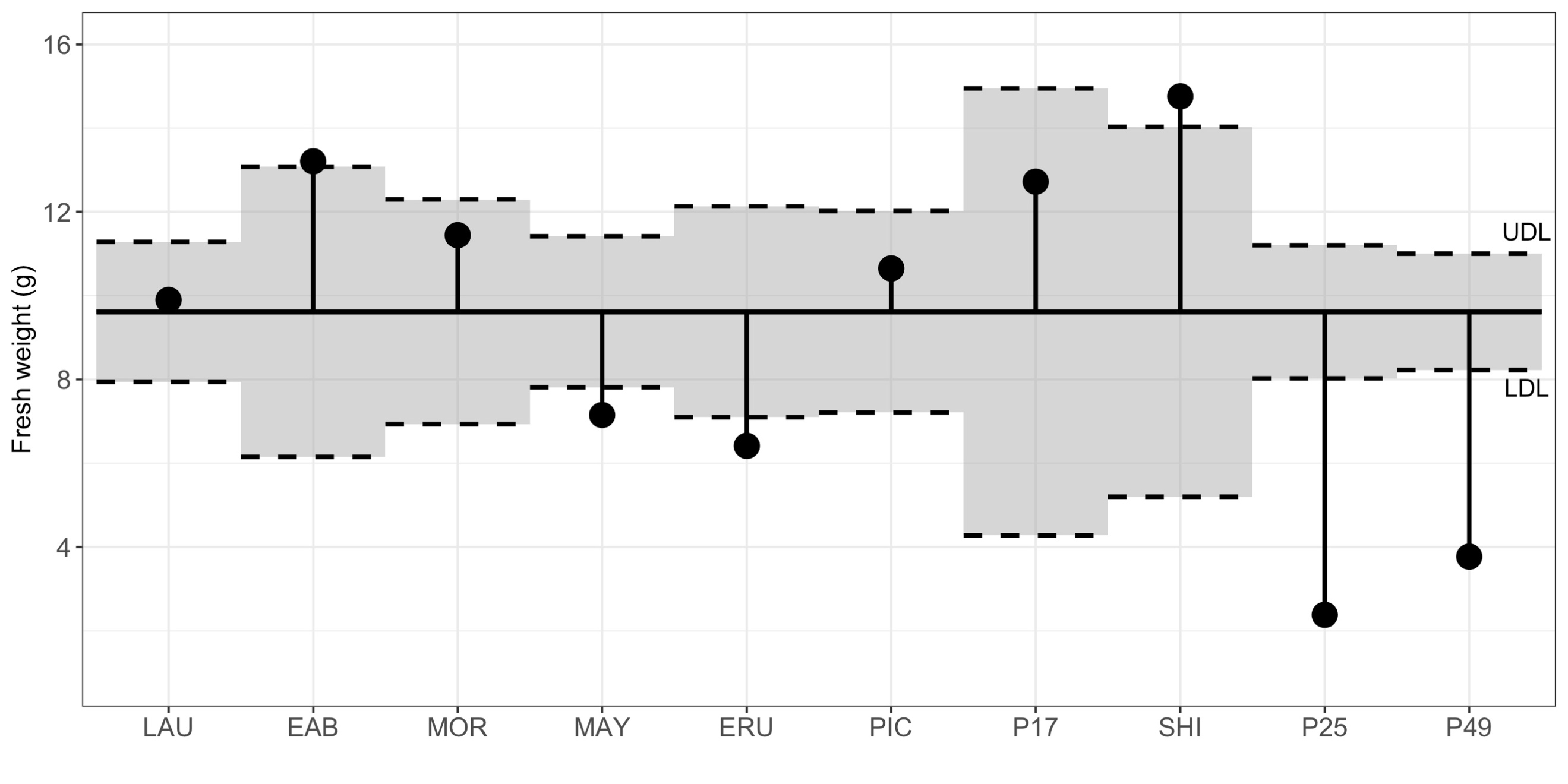


**Supplemental Figure S2B.**

(salt)

**
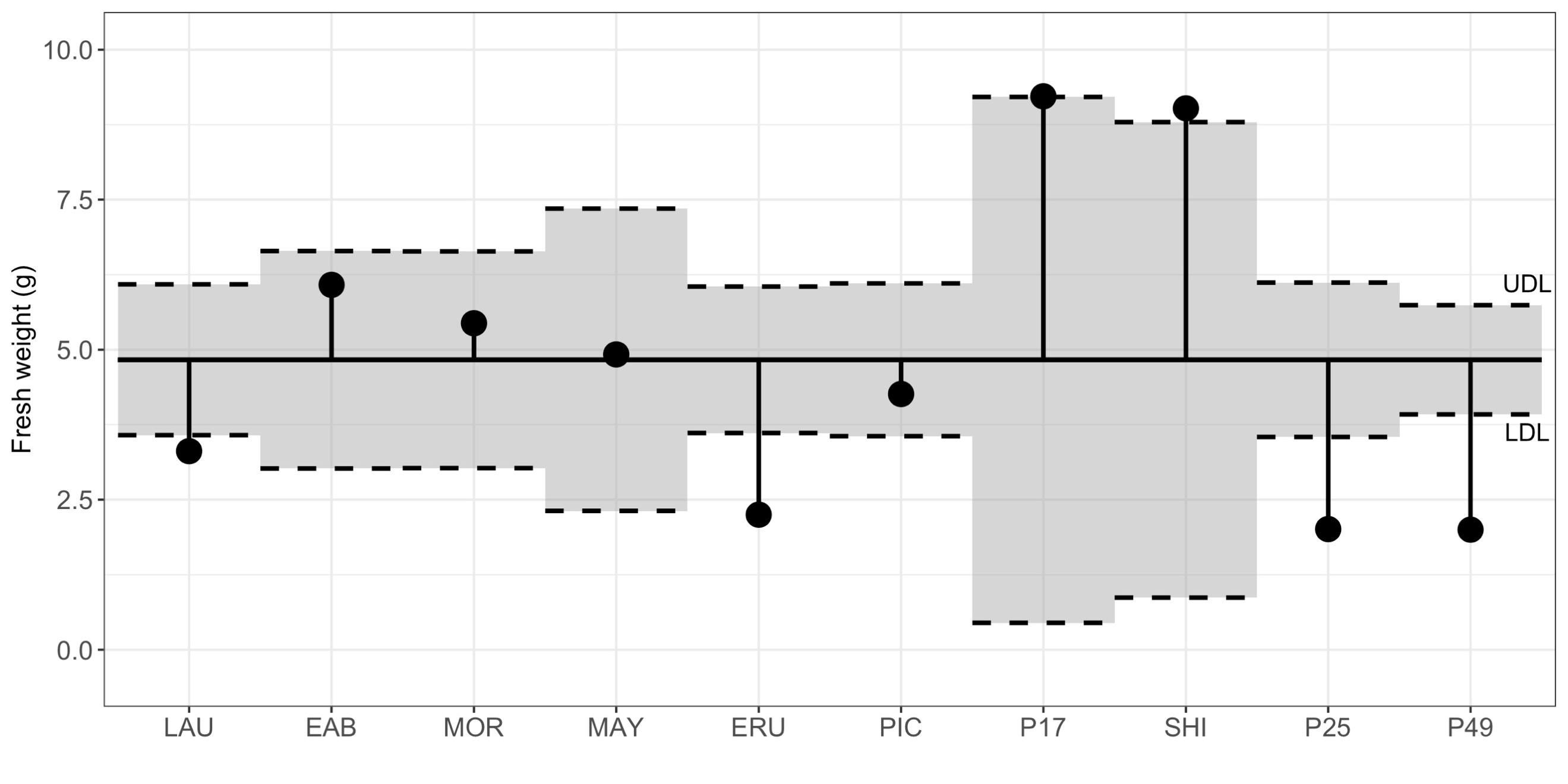
**

**Supplemental Figure S2C.**

(control)

**
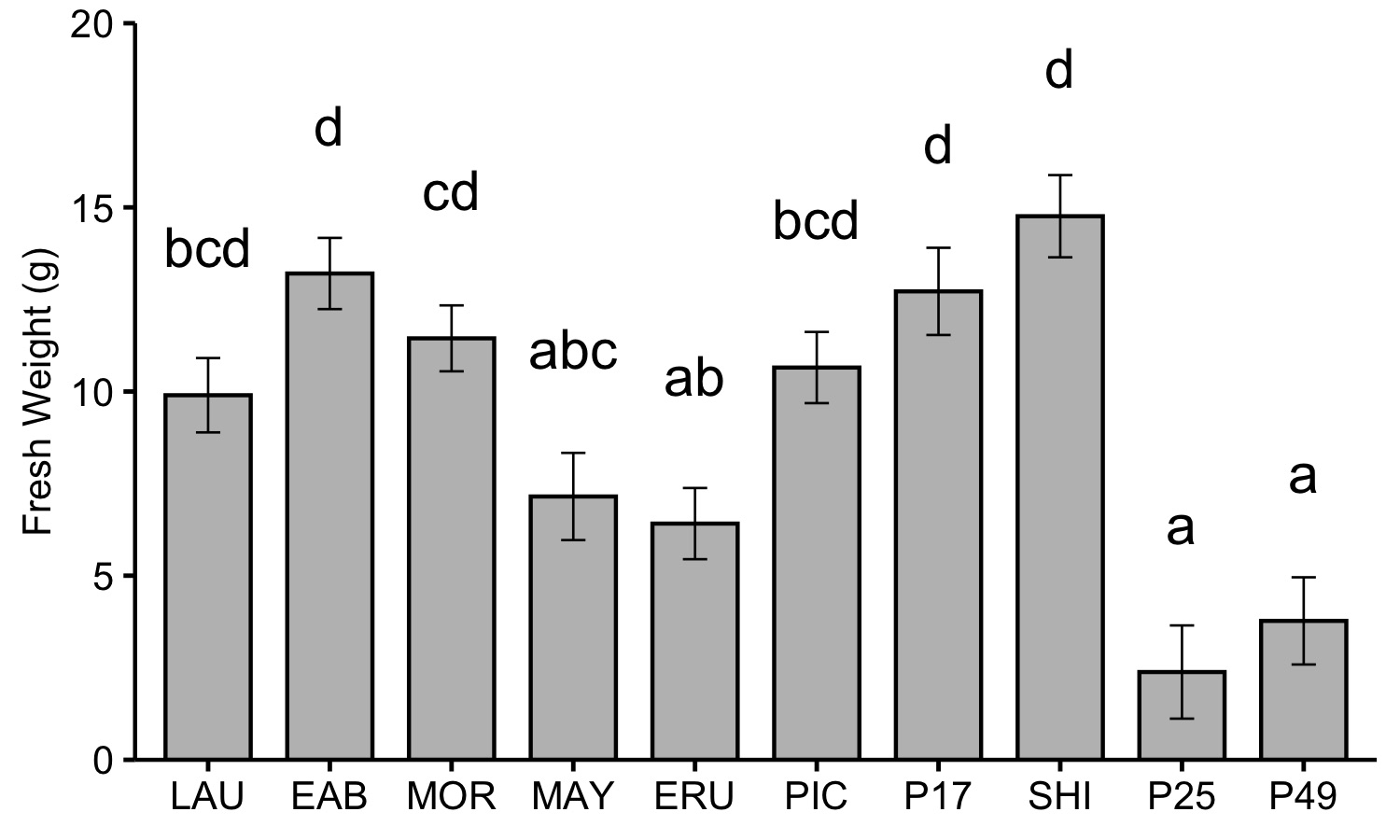
**

**Supplemental Figure S2D.**

(salt)


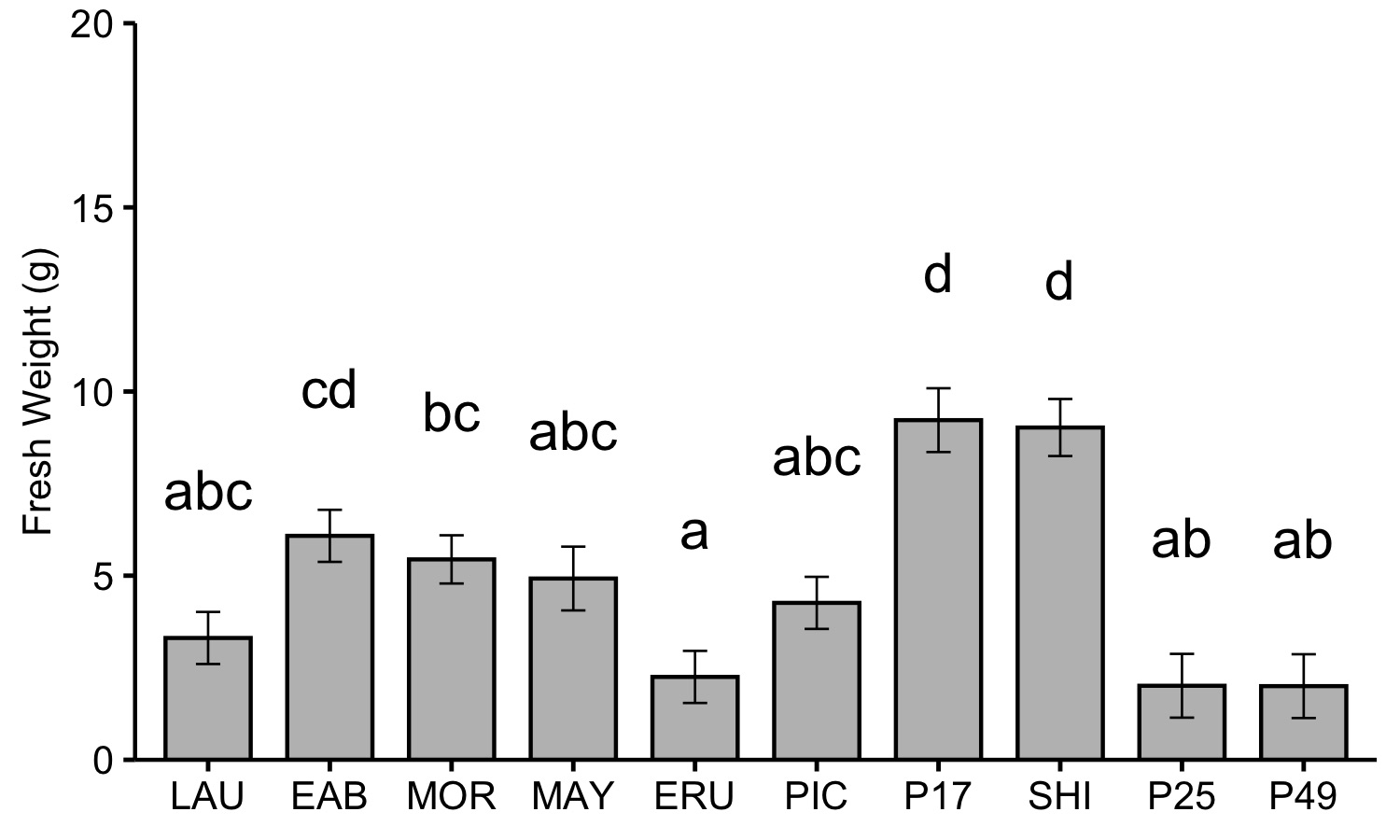


**Supplemental Figure S2**. Analysis of FW means of lettuce plants grown in control (S2A, S2C) or salinity (S2B, S2D) conditions.

ANOM (S2A, S2B): Center solid line indicates the overall mean or ‘generalized mean’. Knob lines represent means of the individual cultivars/accessions. Dashed lines and the gray area within indicate the “decision limit” for the individual means to be significantly higher or lower than the overall mean. UDL, Upper Decision Limit; LDL, Lower Decision Limit. Values are means of two independent experiments, each with at least 4 biological replicates per cultivar/accession per condition.

One-way ANOVA (S2C, S2D): Columns not sharing a letter are significantly different. Values are means of two independent experiments, each with at least 4 biological replicates per cultivar/accession per condition.

**Supplemental Figure S3A.**

(control)

**
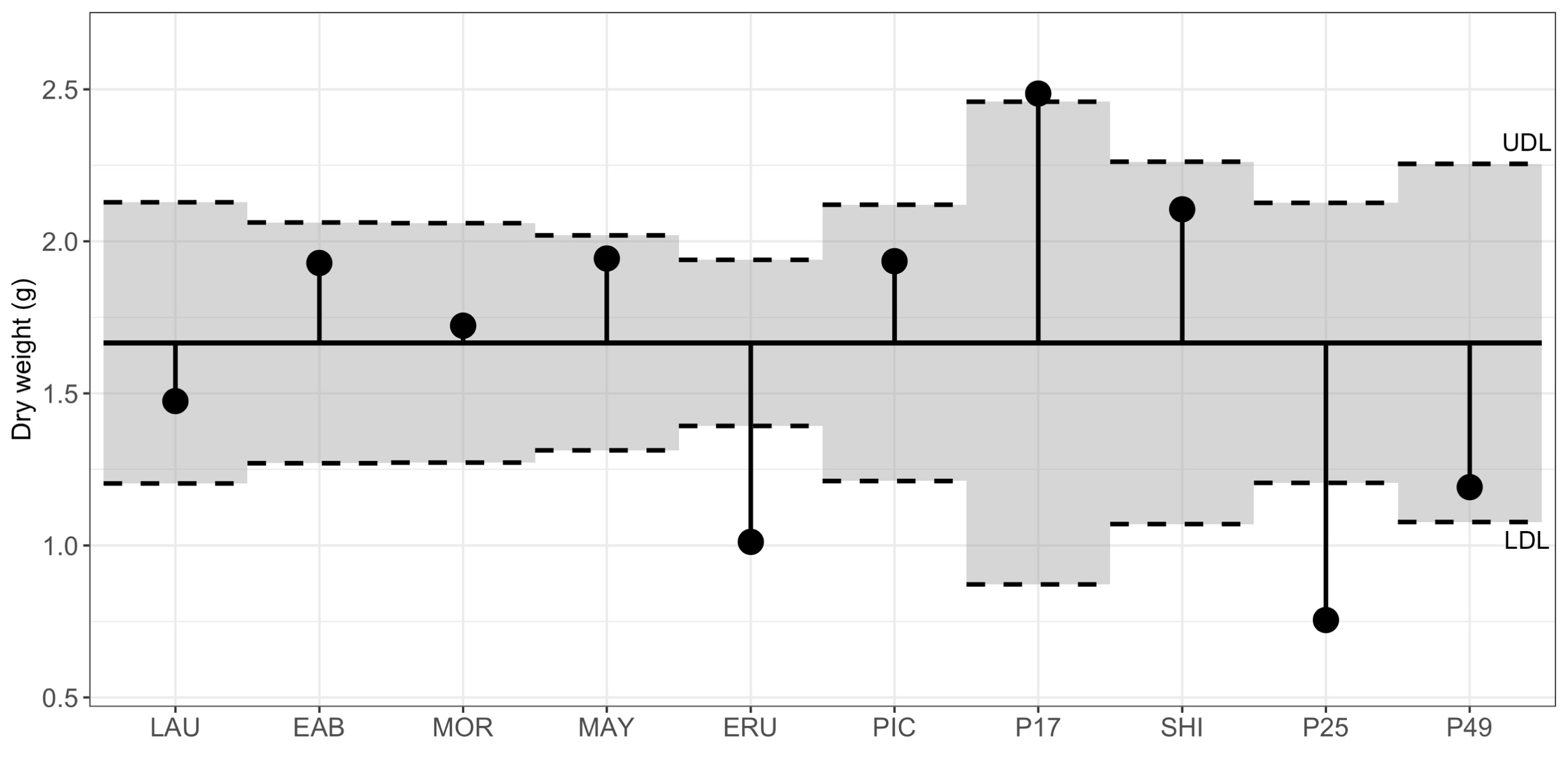
**

**Supplemental Figure S3B.**

(salt)

**
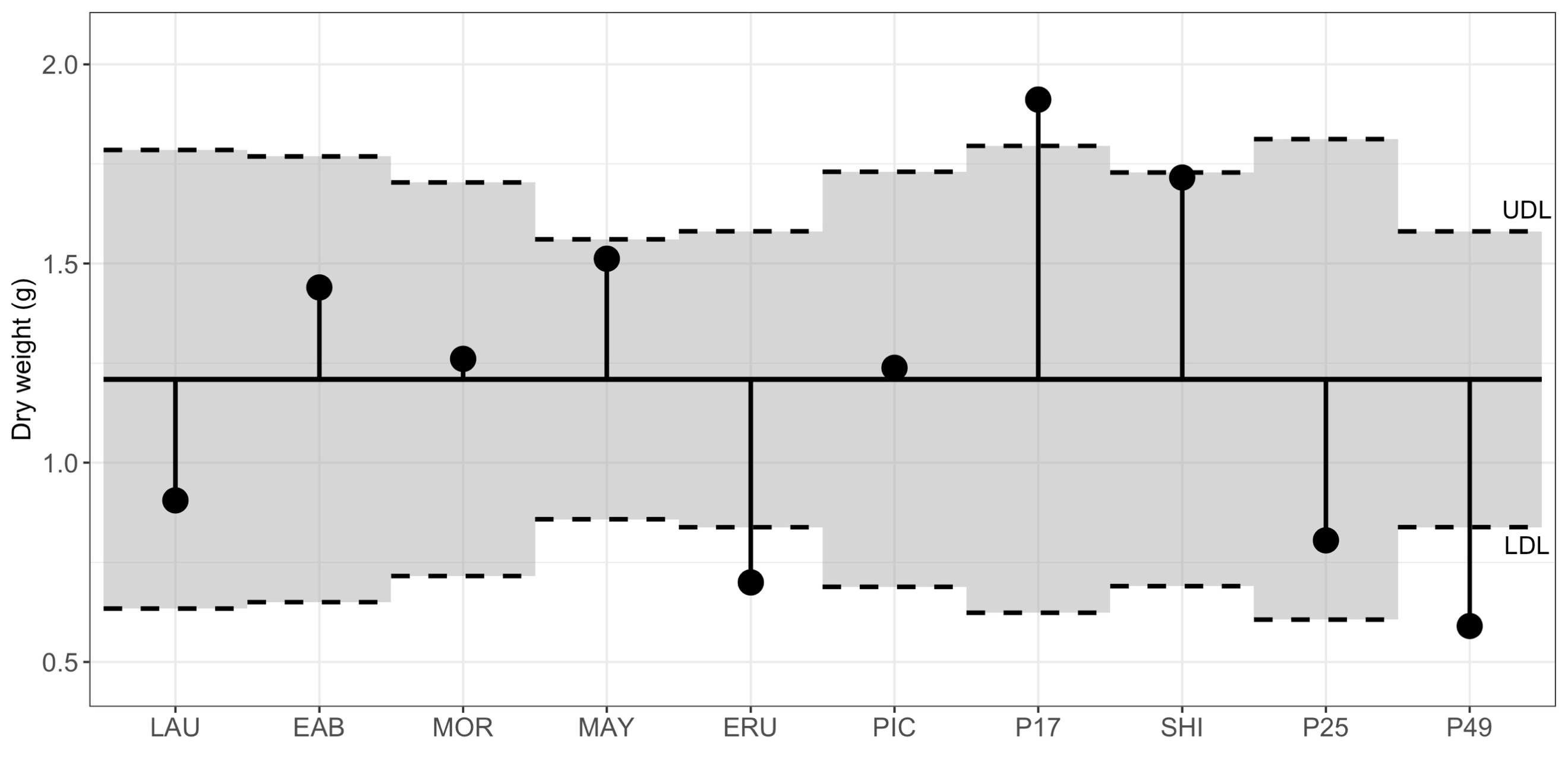
**

**Supplemental Figure S3C.**

(control)


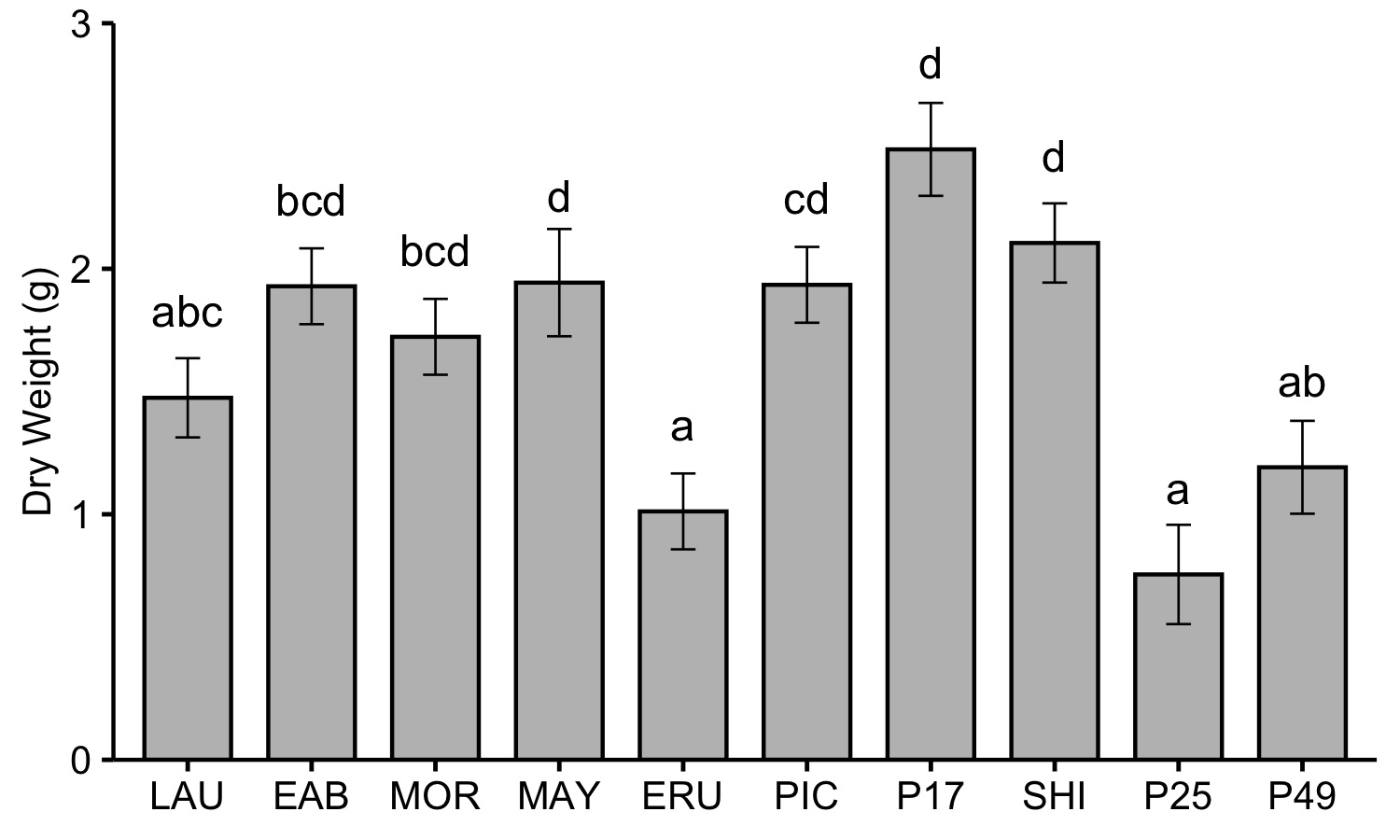


**Supplemental Figure S3D.**

(salt)


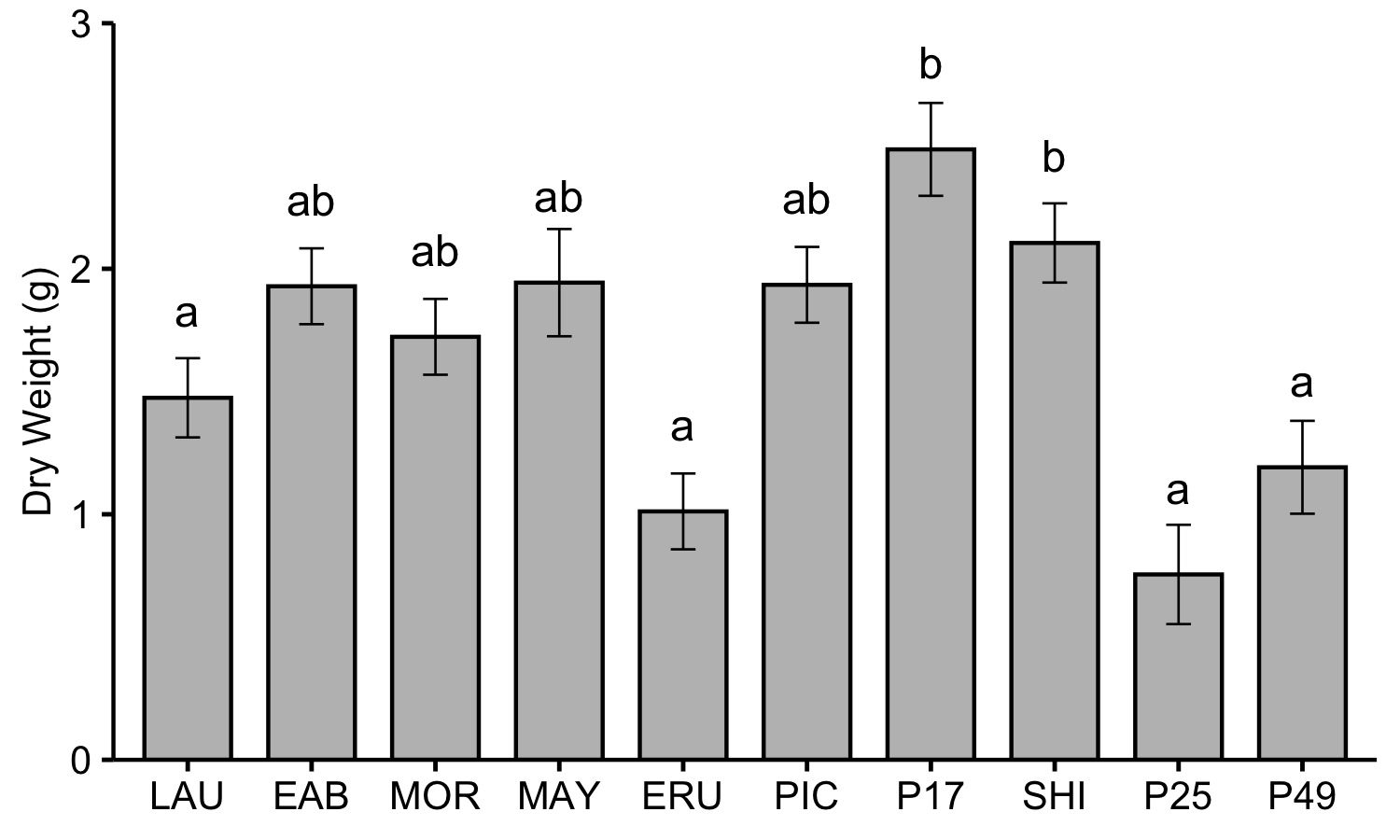


**Supplemental Figure S3**. Analysis of DW means of lettuce plants grown in control (S3A, S3C) or salinity (S3B, S3D) conditions.

ANOM (S3A, S3B): Center solid line indicates the overall mean or ‘generalized mean’. Knob lines represent means of the individual cultivars/accessions. Dashed lines and the gray area within indicate the “decision limit” for the individual means to be significantly higher or lower than the overall mean. UDL, Upper Decision Limit; LDL, Lower Decision Limit. Values are means of two independent experiments, each with at least 4 biological replicates per cultivar/accession per condition.

ANOVA (S3C, S3D): Columns not sharing a letter are significantly different. Values are means of two independent experiments, each with at least 4 biological replicates per cultivar/accession per condition.

**Supplemental Figure S4A.**

(control)


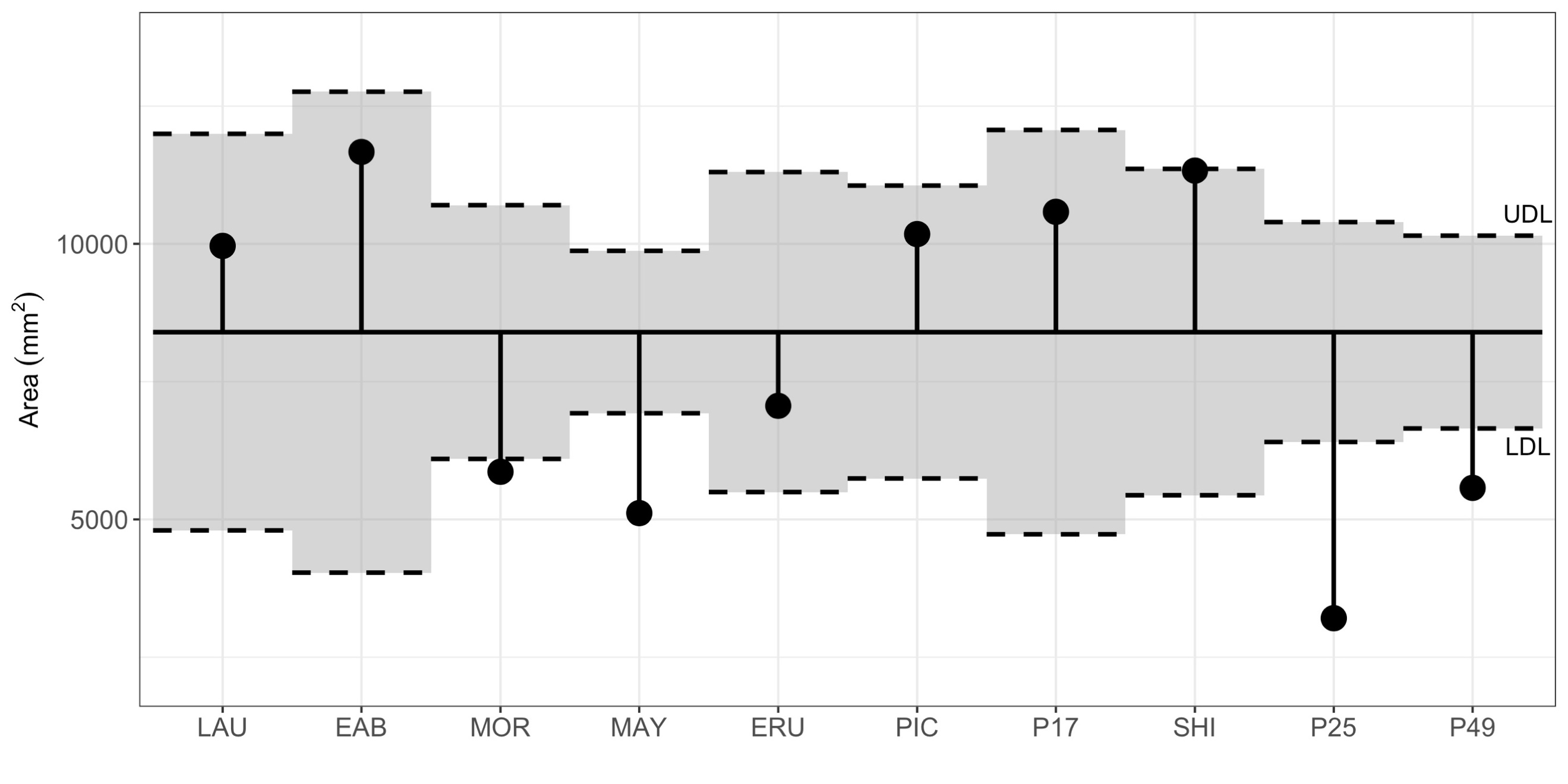


**Supplemental Figure S4B.**

(salt)


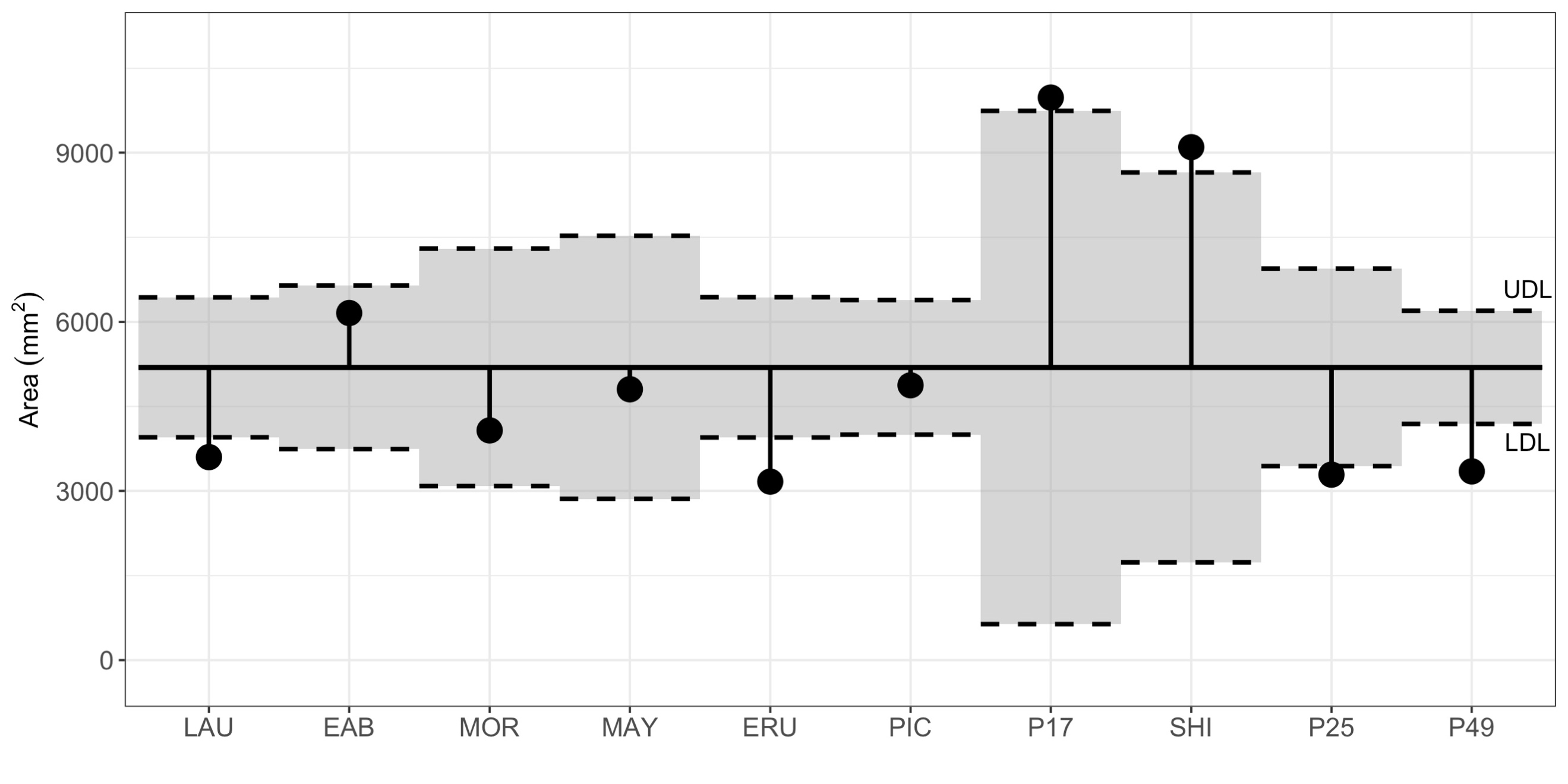


**Supplemental Figure S4C.**

(control)


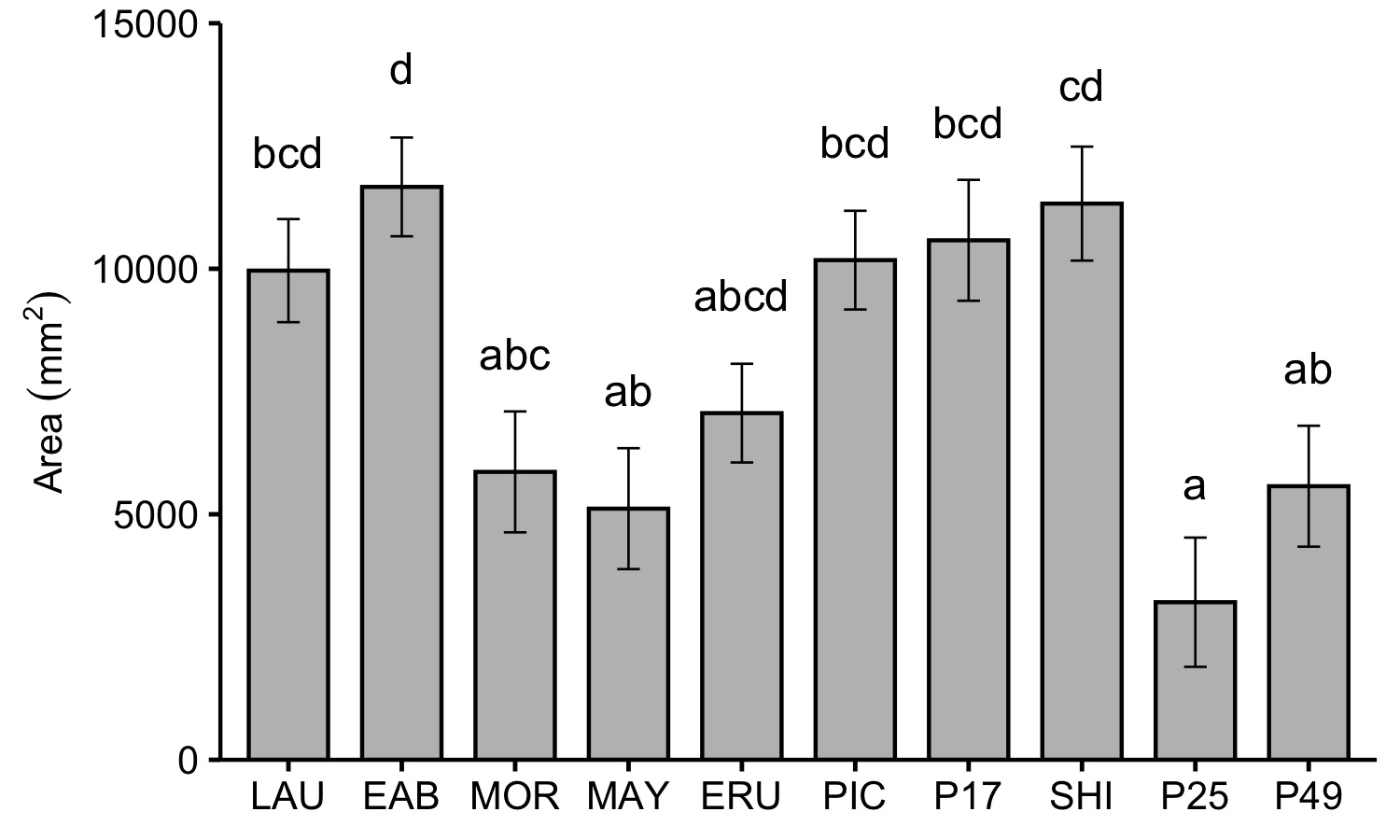


**Supplemental Figure S4D.**

(salt)


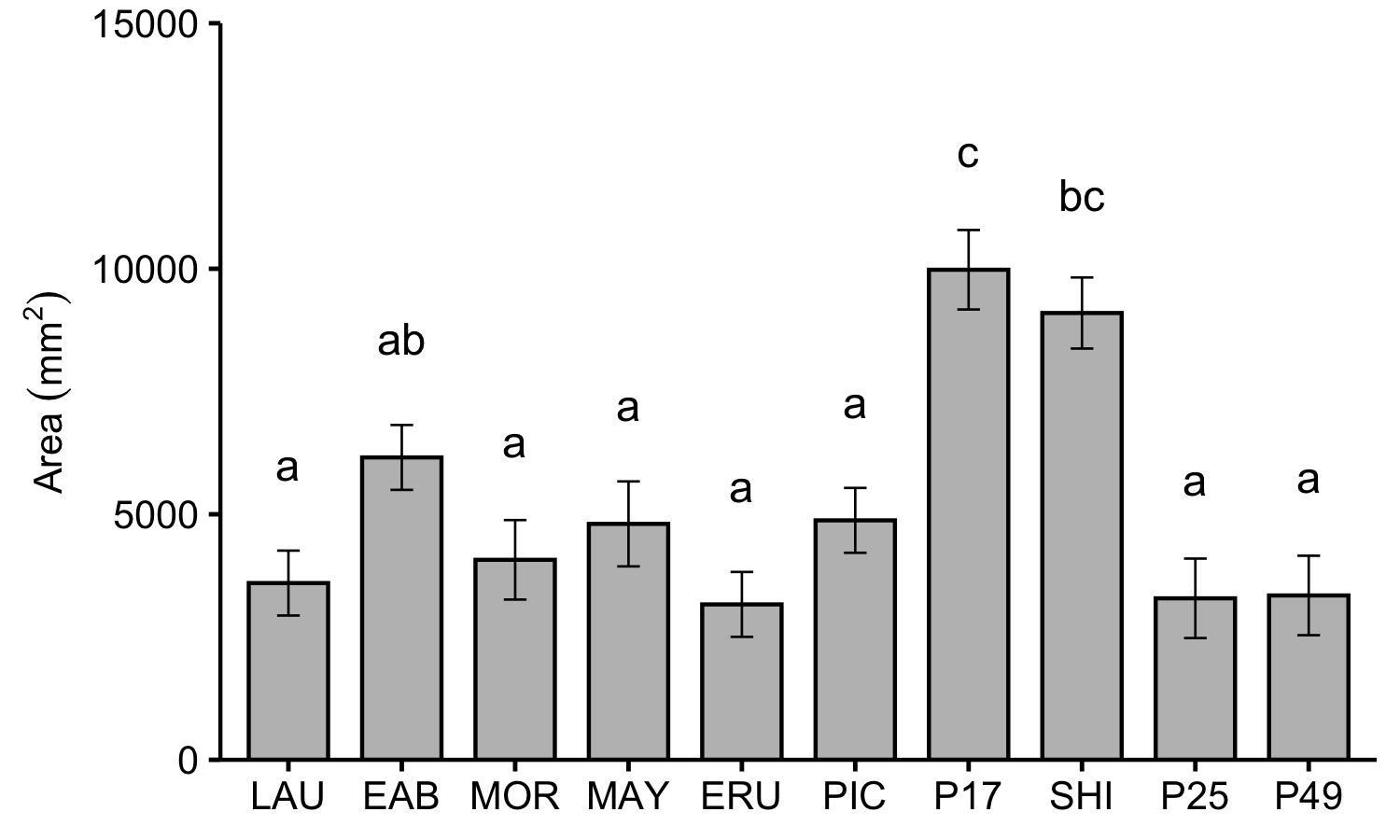


**Supplemental Figure S4**. Analysis of means of total leaf area (also referred to as “area” or LFA) of lettuce plants grown in control (S4A, S4C) or salinity (S4B, S4D) conditions.

ANOM (S4A, S4B): Center solid line indicates the overall mean or ‘generalized mean’. Knob lines represent means of the individual cultivars/accessions. Dashed lines and the gray area within indicate the “decision limit” for the individual means to be significantly higher or lower than the overall mean. UDL, Upper Decision Limit; LDL, Lower Decision Limit. Values are means of two independent experiments, each with at least 4 biological replicates per cultivar/accession per condition.

ANOVA (S4C, S4D): Columns not sharing a letter are significantly different. Values are means of two independent experiments, each with at least 4 biological replicates per cultivar/accession per condition.

**Supplemental Figure S5A.**

(control)


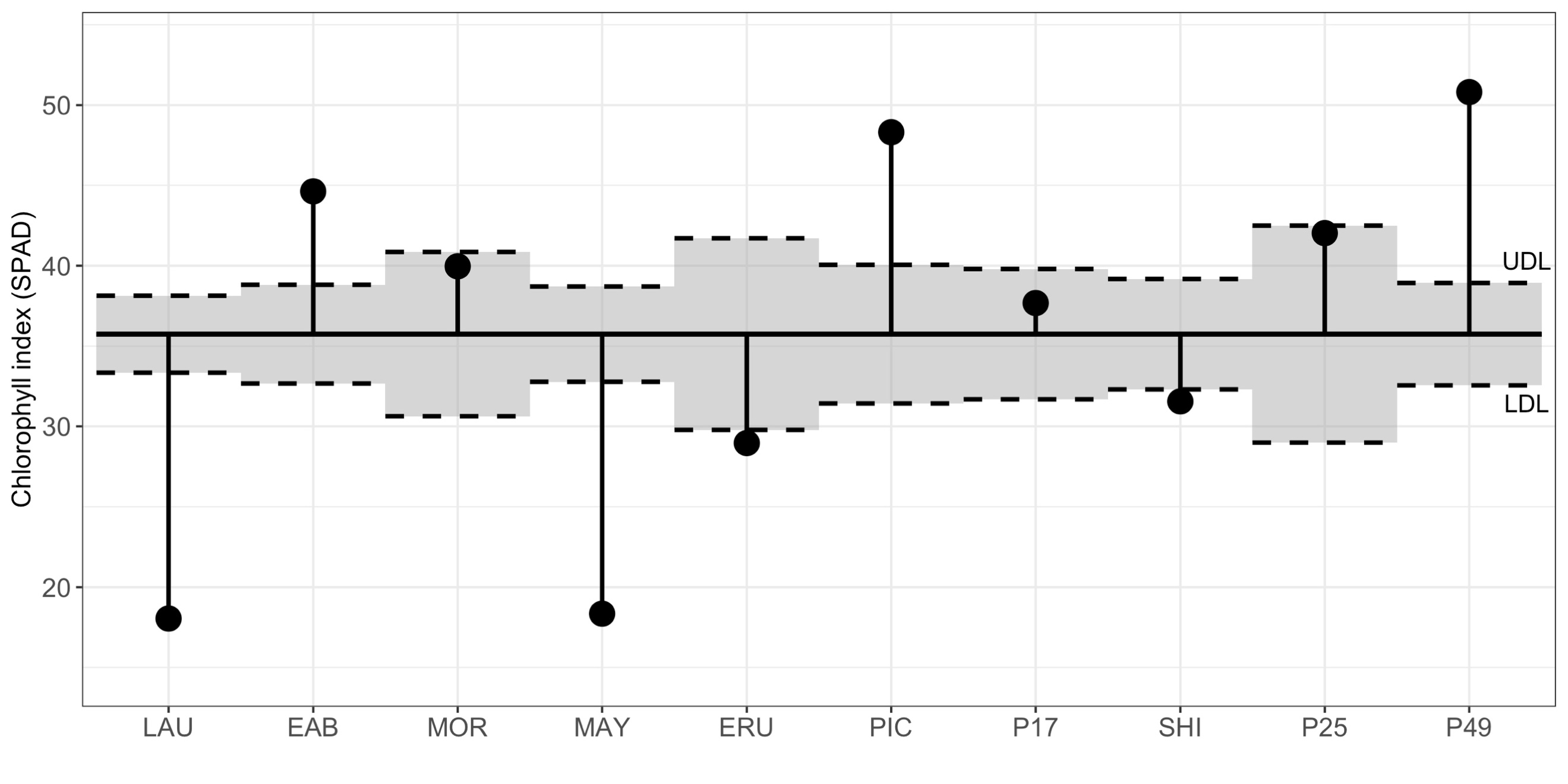


**Supplemental Figure S5B.**

(salt)

**
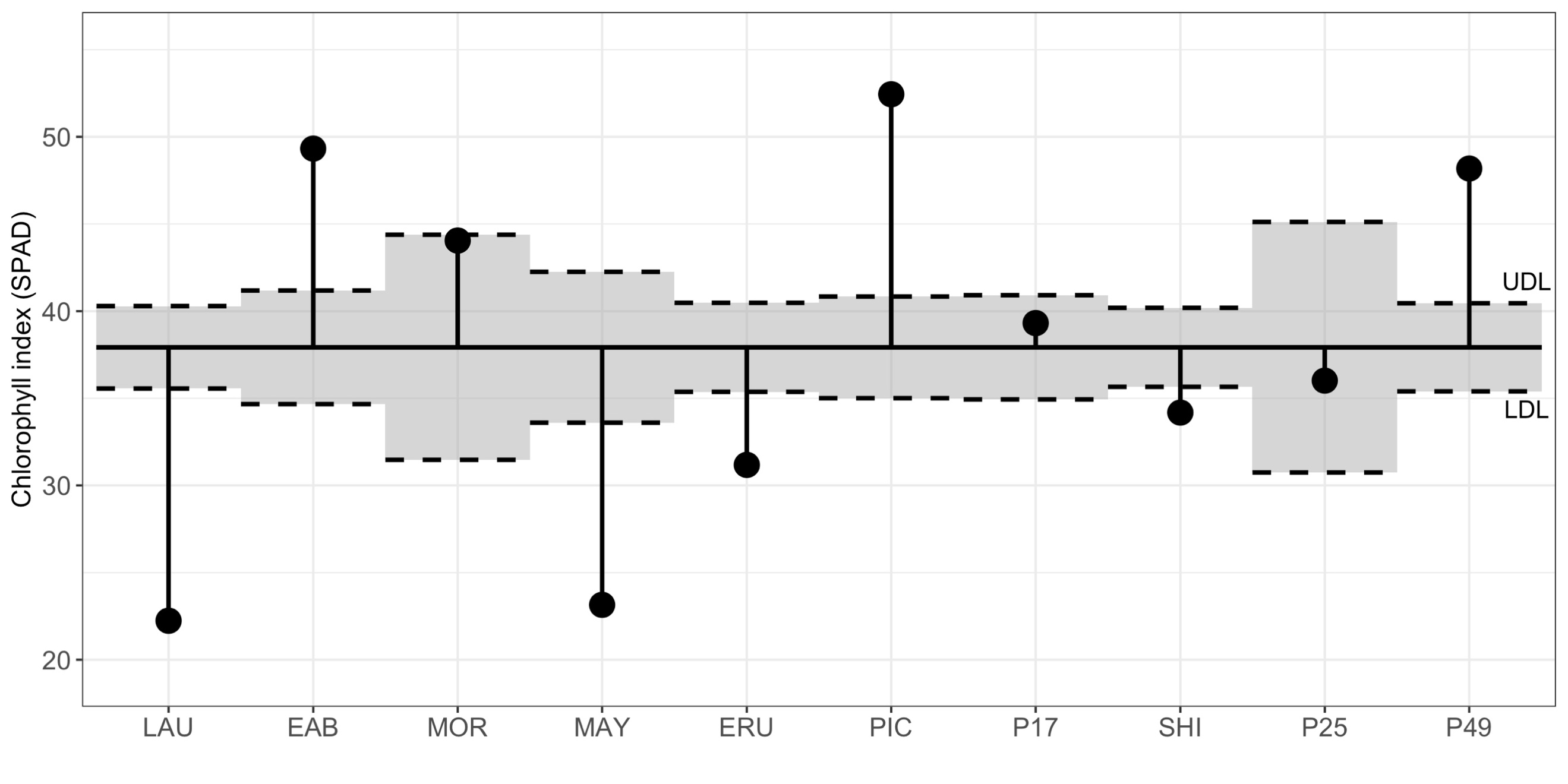
**

**Supplemental Figure S5C.**

(control)

**
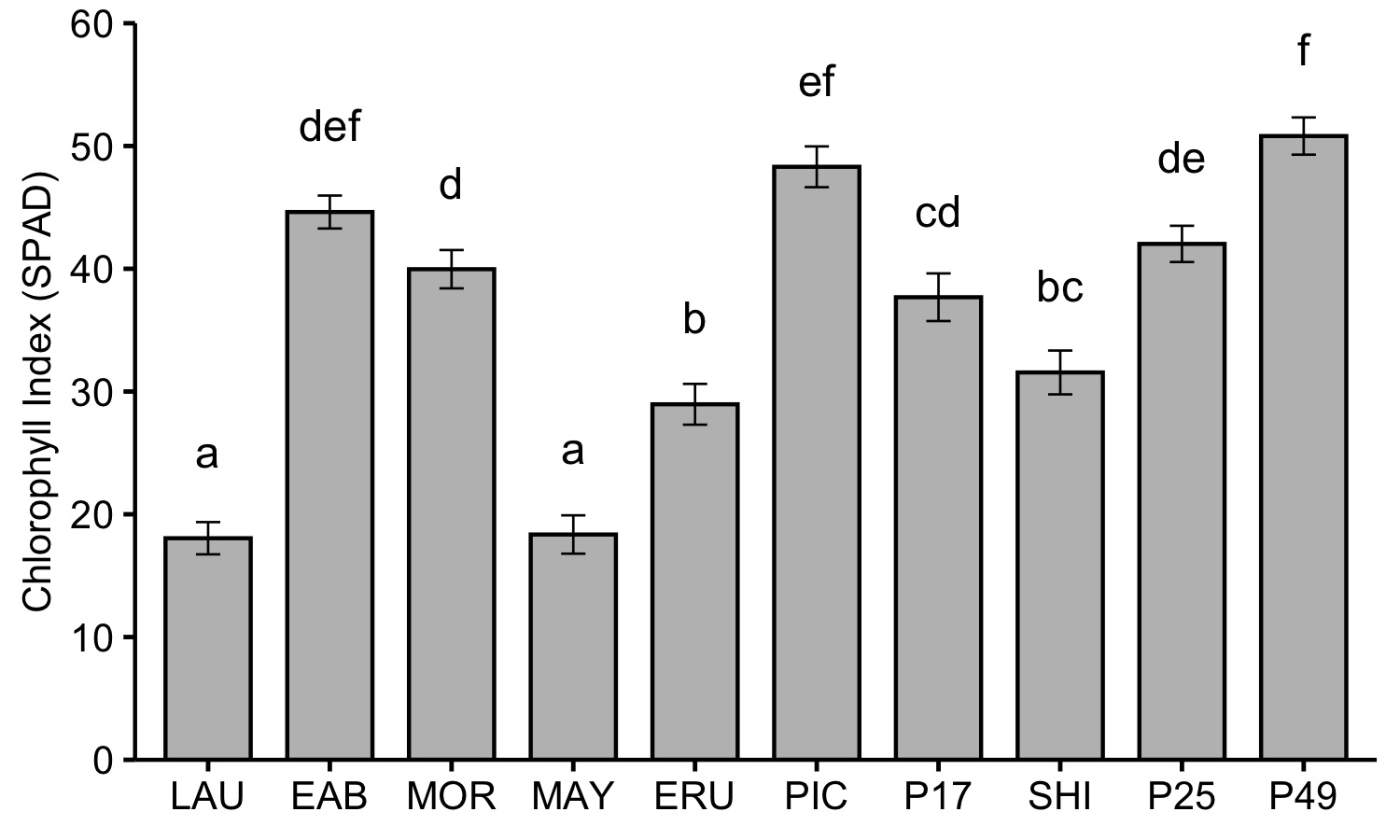
**

**Supplemental Figure S5D.**

(salt)


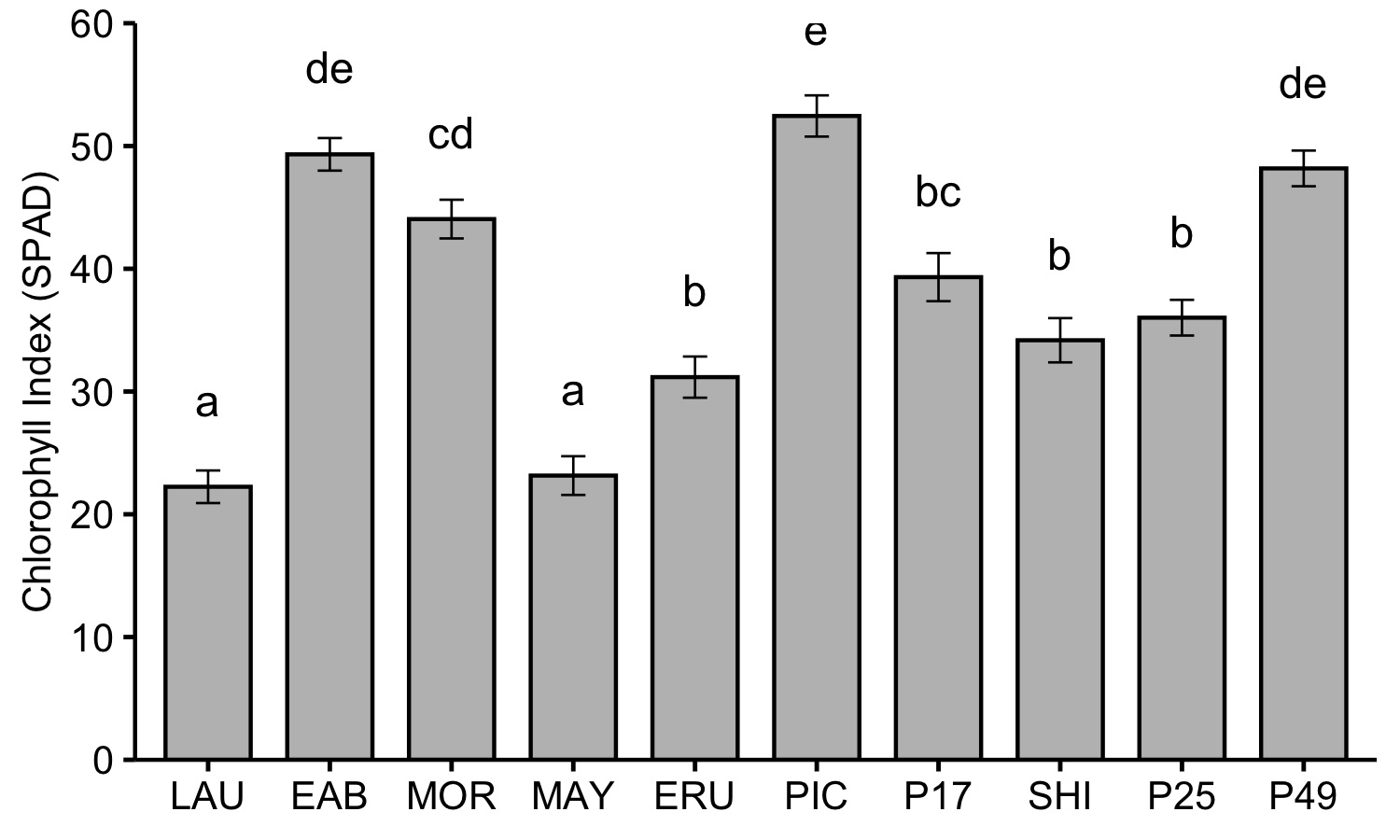


**Supplemental Figure S5**. Analysis of means of chlorophyll index (SPAD) of lettuce plants grown in control (S5A, S5C) or salinity (S5B, S5D) conditions.

ANOM (S5A, S5B): Center solid line indicates the overall mean or ‘generalized mean’. Knob lines represent means of the individual cultivars/accessions. Dashed lines and the gray area within indicate the “decision limit” for the individual means to be significantly higher or lower than the overall mean. UDL, Upper Decision Limit; LDL, Lower Decision Limit. Values are means of five independent experiments, each with at least 4 biological replicates per cultivar/accession per condition.

ANOVA (S5C, S5D): Columns not sharing a letter are significantly different. Values are means of five independent experiments, each with at least 4 biological replicates per cultivar/accession per condition.

**Supplemental Figure S6A.**

(control)


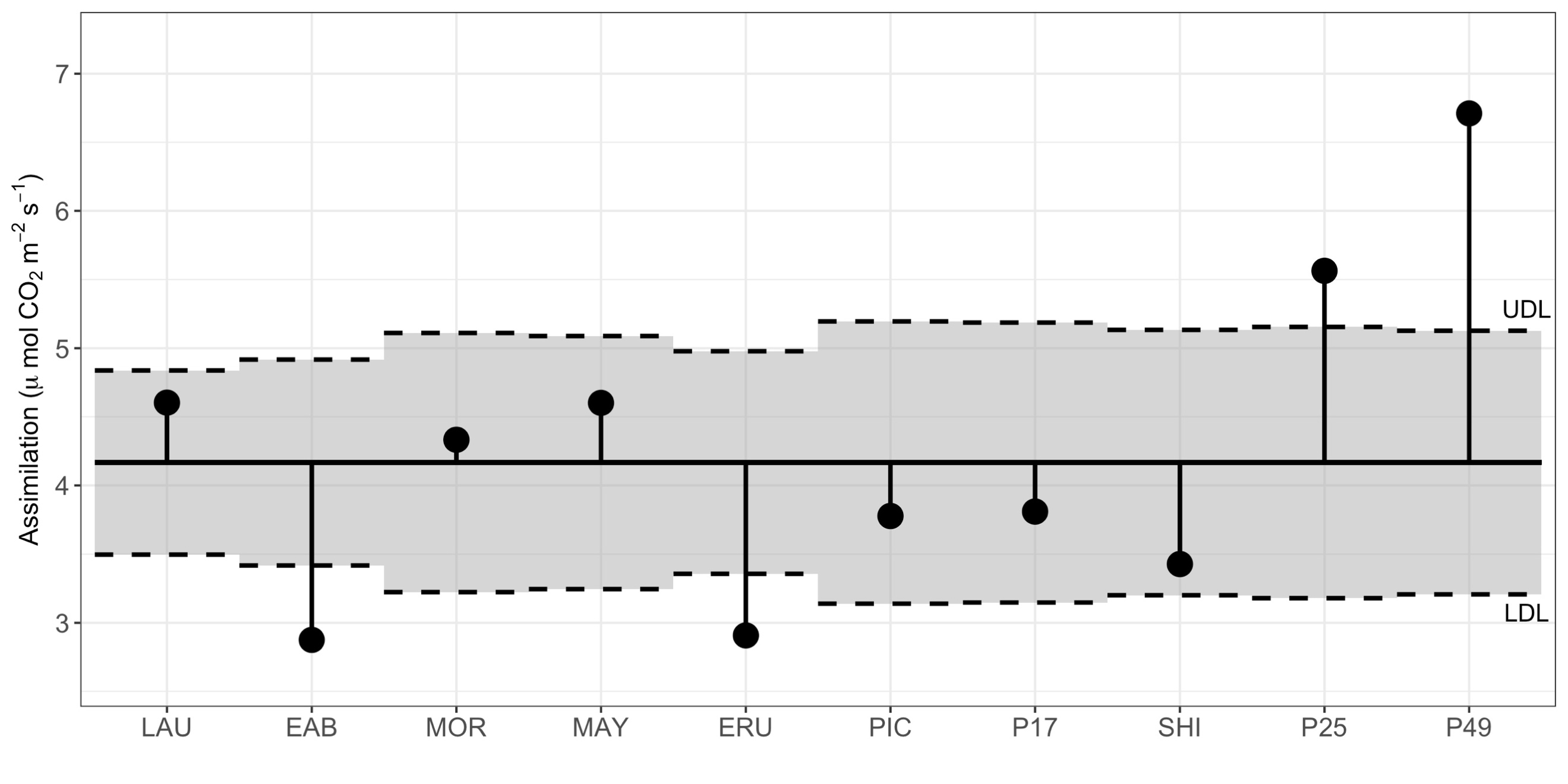


**Supplemental Figure S6B.**

(salt)


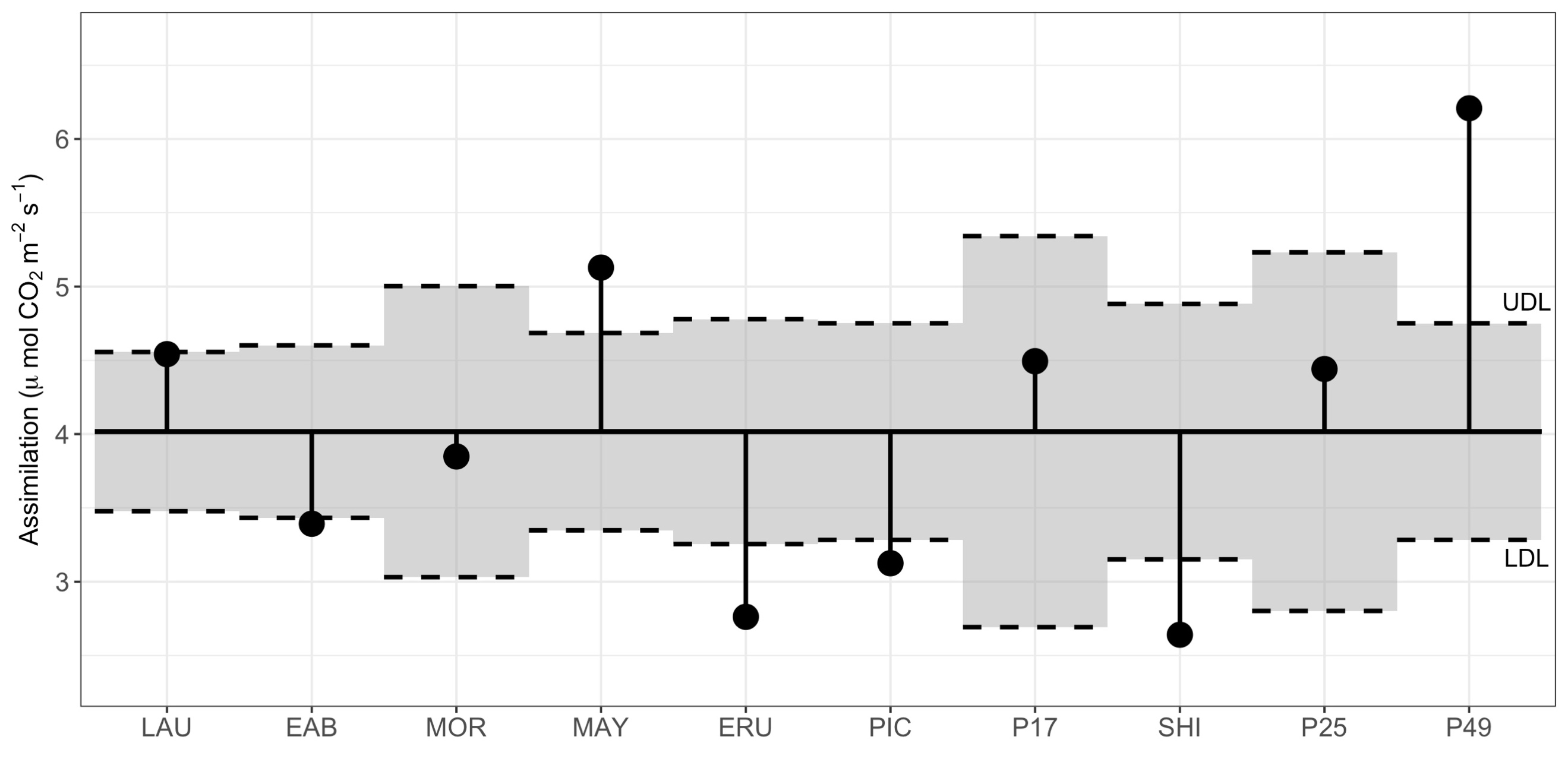


**Supplemental Figure S6C.**

(control)


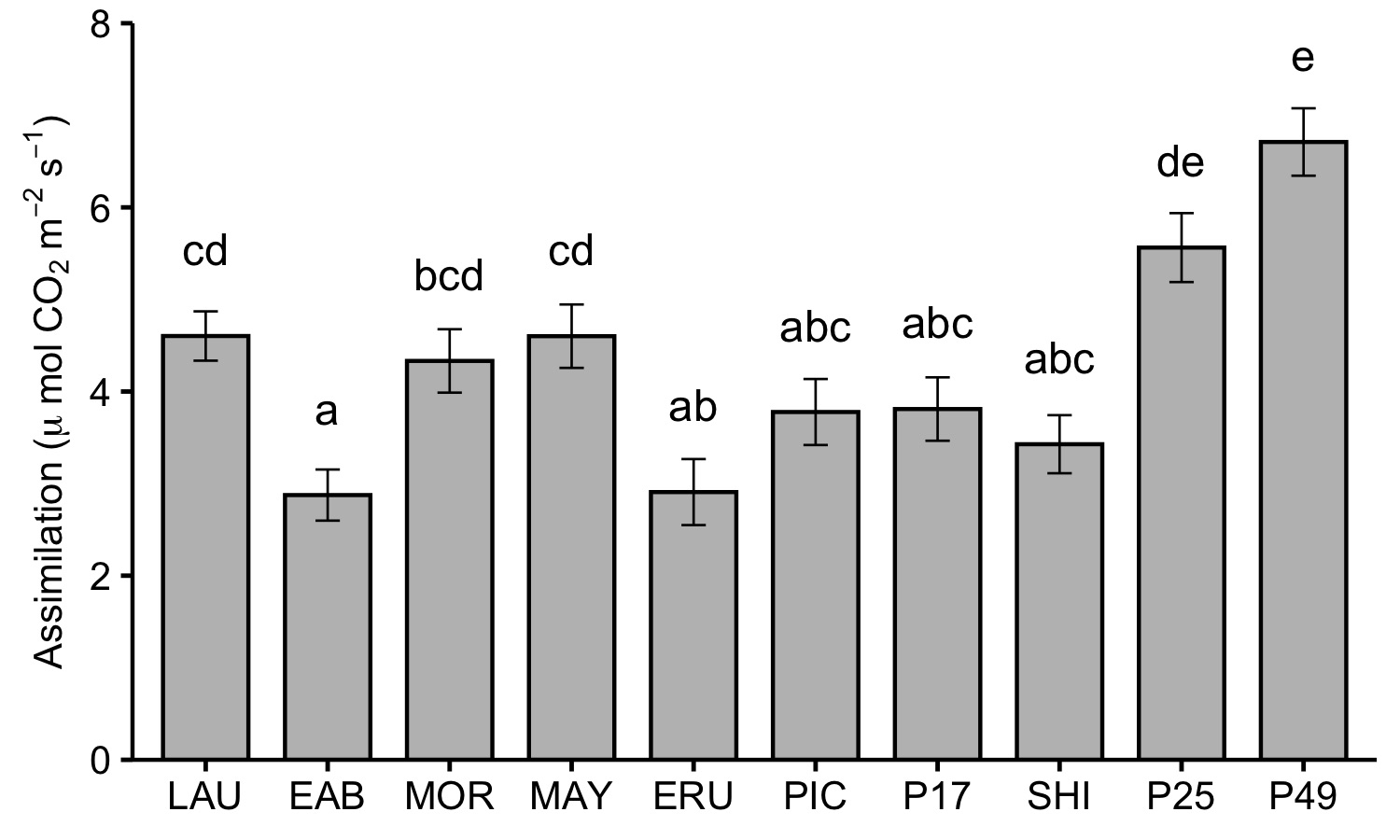


**Supplemental Figure S6D.**

(salt)


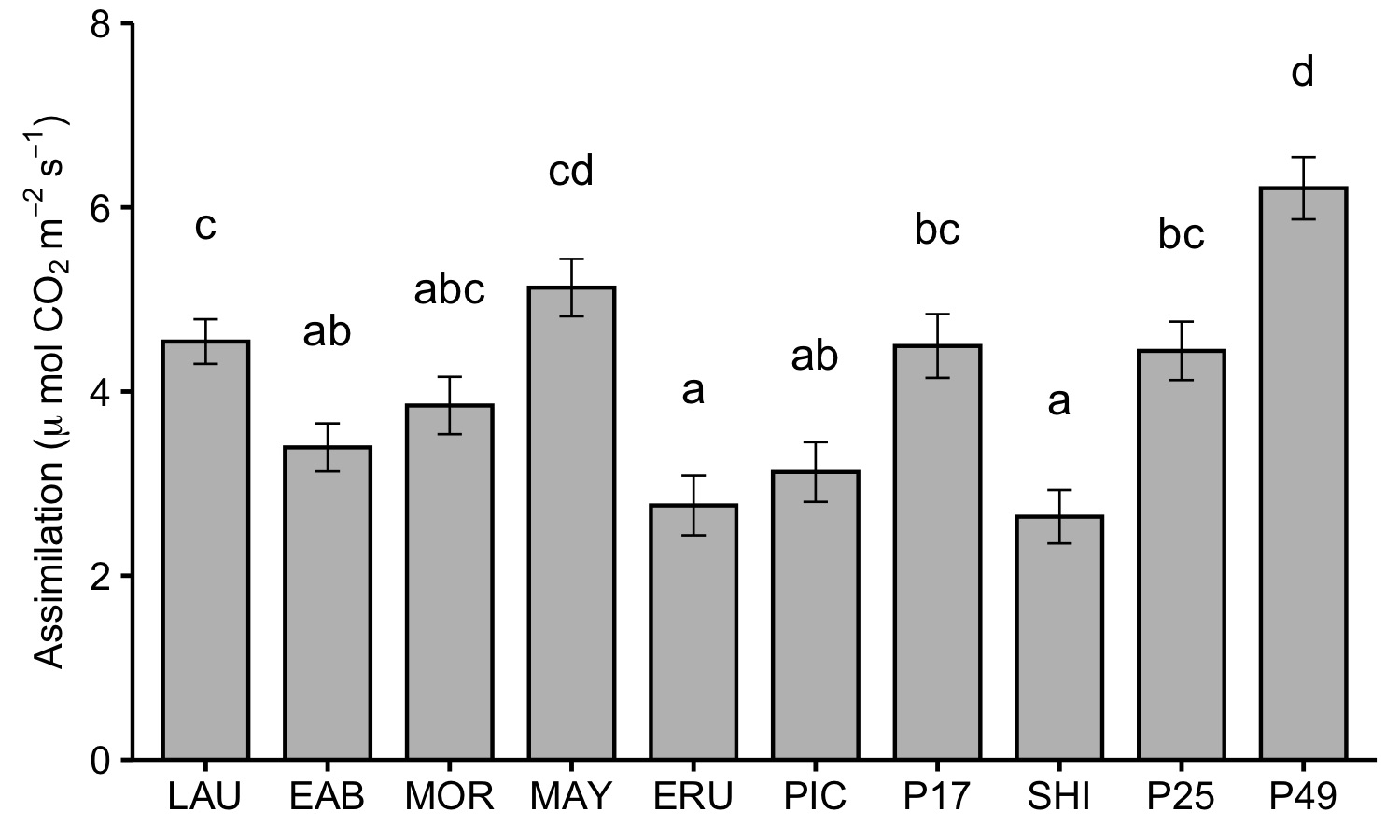


**Supplemental Figure S6**. Analysis of means of Photosynthetic CO_2_ Assimilation in lettuce plants grown in control (S6A, S6C) or salinity (S6B, S6D) conditions.

ANOM (S6A, S6B): Center solid line indicates the overall mean or ‘generalized mean’. Knob lines represent means of the individual cultivars/accessions. Dashed lines and the gray area within indicate the “decision limit” for the individual means to be significantly higher or lower than the overall mean. UDL, Upper Decision Limit; LDL, Lower Decision Limit. Values are means of four independent experiments, each with at least 4 biological replicates per cultivar/accession per condition.

ANOVA (S6C, S6D): Columns not sharing a letter are significantly different. Values are means of four independent experiments, each with at least 4 biological replicates per cultivar/accession per condition.

**Supplemental Table S1.** Intercellular CO_2_ concentration (CI) in control and salinity treatments.

| Cultivar | Treatment | CI (μmol CO_2_ mol^-1^ air) | % reduction | Significance |
| --- | --- | --- | --- | --- |
| LAU | control | 338 ± 43 | 7 | ns |
| LAU | salt | 314 ± 24 |  |  |
| EAB | control | 364 ± 18 | 18 | ns |
| EAB | salt | 297 ± 53 |  |  |
| MOR | control | 355 ± 42 | -85 | ns |
| MOR | salt | 656 ± 311 |  |  |
| MAY | control | 381 ± 18 | 91 | ns |
| MAY | salt | 36 ± 181 |  |  |
| ERU | control | 586 ± 219 | 22 | ns |
| ERU | salt | 456 ± 17 |  |  |
| PIC | control | 151 ± 236 | -90 | ns |
| PIC | salt | 325 ± 103 |  |  |
| P17 | control | 177 ± 152 | -84 | ns |
| P17 | salt | 325 ± 103 |  |  |
| SHI | control | 314 ± 73 | -32 | ns |
| SHI | salt | 415 ± 64 |  |  |
| P25 | control | 431 ± 78 | 29 | ns |
| P25 | salt | 304 ± 29 |  |  |
| P49 | control | 236 ± 49 | -81 | ns |
| P49 | salt | 427 ± 252 |  |  |

Values are means and standard errors of four independent experiments, each with at least four biological replicates per genotype per condition. Asterisks indicate statistical significance as calculated by the *t*-test. * (P ≤ 0.05), ** (P ≤ 0.01), *** (P ≤ 0.001), **** (P ≤ 0.0001), ns = not significant (P > 0.05).

**Supplemental Table S2.** Stomatal conductance to H_2_O (Cond) in control and salinity treatments.

| Cultivar | Treatment | Cond (mol H_2_O m^-2^ s^-1^) | % reduction | Significance |
| --- | --- | --- | --- | --- |
| LAU | control | 0.051 ± 0.006 | 3 | ns |
| LAU | salt | 0.050 ± 0.005 |  |  |
| EAB | control | 0.078 ± 0.017 | 27 | ns |
| EAB | salt | 0.057 ± 0.008 |  |  |
| MOR | control | 0.045 ± 0.007 | 61 | ** |
| MOR | salt | 0.018 ± 0.005 |  |  |
| MAY | control | 0.086 ± 0.010 | 77 | *** |
| MAY | salt | 0.020 ± 0.004 |  |  |
| ERU | control | 0.058 ± 0.014 | 83 | ns |
| ERU | salt | 0.010 |  |  |
| PIC | control | 0.017 ± 0.005 | -25 | ns |
| PIC | salt | 0.021 ± 0.004 |  |  |
| P17 | control | 0.049 ± 0.009 | 27 | ns |
| P17 | salt | 0.036 ± 0.007 |  |  |
| SHI | control | 0.051 ± 0.008 | -3 | ns |
| SHI | salt | 0.052 ± 0.013 |  |  |
| P25 | control | 0.137 ± 0.019 | 43 | * |
| P25 | salt | 0.079 ± 0.014 |  |  |
| P49 | control | 0.122 ± 0.023 | 44 | * |
| P49 | salt | 0.069 ± 0.011 |  |  |

Values are means and standard errors of four independent experiments, each with at least four biological replicates per genotype per condition except ERU salt, for which only one replicate was available. Asterisks indicate statistical significance as calculated by the *t*-test. * (P ≤ 0.05), ** (P ≤ 0.01), *** (P ≤ 0.001), **** (P ≤ 0.0001), ns = not significant (P > 0.05). Note: Only a single replicate was available for ERU salt, thus the statistical comparison between ERU control and ERU salt may be affected.

**Supplemental Table S3.** Transpiration rate (Trmmol) in control and salinity treatments.

| Cultivar | Treatment | Trmmol (mmol H_2_O m^-2^ s^-1^) | % reduction | Significance |
| --- | --- | --- | --- | --- |
| LAU | control | 0.38 ± 0.04 | -5 | ns |
| LAU | salt | 0.40 ± 0.04 |  |  |
| EAB | control | 0.58 ± 0.12 | -2 | ns |
| EAB | salt | 0.59 ± 0.08 |  |  |
| MOR | control | 0.27± 0.04 | 57 | * |
| MOR | salt | 0.12 ± 0.03 |  |  |
| MAY | control | 0.40 ± 0.04 | 72 | **** |
| MAY | salt | 0.25 ± 0.06 |  |  |
| ERU | control | 0.058 ± 0.014 | 80 | ns |
| ERU | salt | 0.05 |  |  |
| PIC | control | 0.07 ± 0.02 | -132 | * |
| PIC | salt | 0.15 ± 0.03 |  |  |
| P17 | control | 0.47 ± 0.09 | 44 | ns |
| P17 | salt | 0.26 ± 0.05 |  |  |
| SHI | control | 0.48 ± 0.09 | 4 | ns |
| SHI | salt | 0.46 ± 0.11 |  |  |
| P25 | control | 0.71 ± 0.08 | 31 | * |
| P25 | salt | 0.49 ± 0.08 |  |  |
| P49 | control | 0.66 ± 0.11 | 36 | ns |
| P49 | salt | 0.42 ± 0.06 |  |  |

Values are means and standard errors of four independent experiments, each with at least four biological replicates per genotype per condition except ERU salt, for which only one replicate was available. Asterisks indicate statistical significance as calculated by the *t*-test. * (P ≤ 0.05), ** (P ≤ 0.01), *** (P ≤ 0.001), **** (P ≤ 0.0001), ns = not significant (P > 0.05). Note: Only a single replicate was available for ERU salt, thus the statistical comparison between ERU control and ERU salt may be affected.

**Supplemental Table S4.** Calculated leaf thickness of aerial tissue in control and salinity treatments.

| Cultivar | Treatment | Calculated leaf thickness (mg/ cm^2^) | % reduction | Significance |
| --- | --- | --- | --- | --- |
| LAU | control | 110 ± 9 | 9 | ns |
| LAU | salt | 100 ± 16 |  |  |
| EAB | control | 124 ± 9 | 22 | * |
| EAB | salt | 97 ± 4 |  |  |
| MOR | control | 145 ± 6 | 21 | ** |
| MOR | salt | 115 ± 8 |  |  |
| MAY | control | 141 ± 8 | 26 | ** |
| MAY | salt | 105 ± 5 |  |  |
| ERU | control | 93 ± 5 | 28 | ** |
| ERU | salt | 67 ± 5 |  |  |
| PIC | control | 107 ± 3 | 19 | ** |
| PIC | salt | 86 ± 4 |  |  |
| P17 | control | 118 ± 7 | 22 | * |
| P17 | salt | 93 ± 6 |  |  |
| SHI | control | 129 ± 5 | 9 | ns |
| SHI | salt | 117 ± 31 |  |  |
| P25 | control | 77 ± 10 | 19 | ns |
| P25 | salt | 62 ± 7 |  |  |
| P49 | control | 68 ± 3 | 12 | ns |
| P49 | salt | 60 ± 3 |  |  |

Leaf thickness was calculated by the formula FW/LFA. Values are means and standard errors of two independent experiments, each with at least four biological replicates per genotype per condition. Asterisks indicate statistical significance as calculated by the *t*-test. * (P ≤ 0.05), ** (P ≤ 0.01), *** (P ≤ 0.001), **** (P ≤ 0.0001), ns = not significant (P > 0.05).
