# Supplementary material for: Phenomic and Physiological Analysis of Salinity Effects on Lettuce
Source: Sensors (Basel). 2019 Nov 5;19(21):4814. doi: 10.3390/s19214814 (PMC6864466; doi:10.3390/s19214814)
Supplement: Supplementary file 1 [file sensors-19-04814-s001.zip › Supplemental Data 5.pptx]

## Slide 1
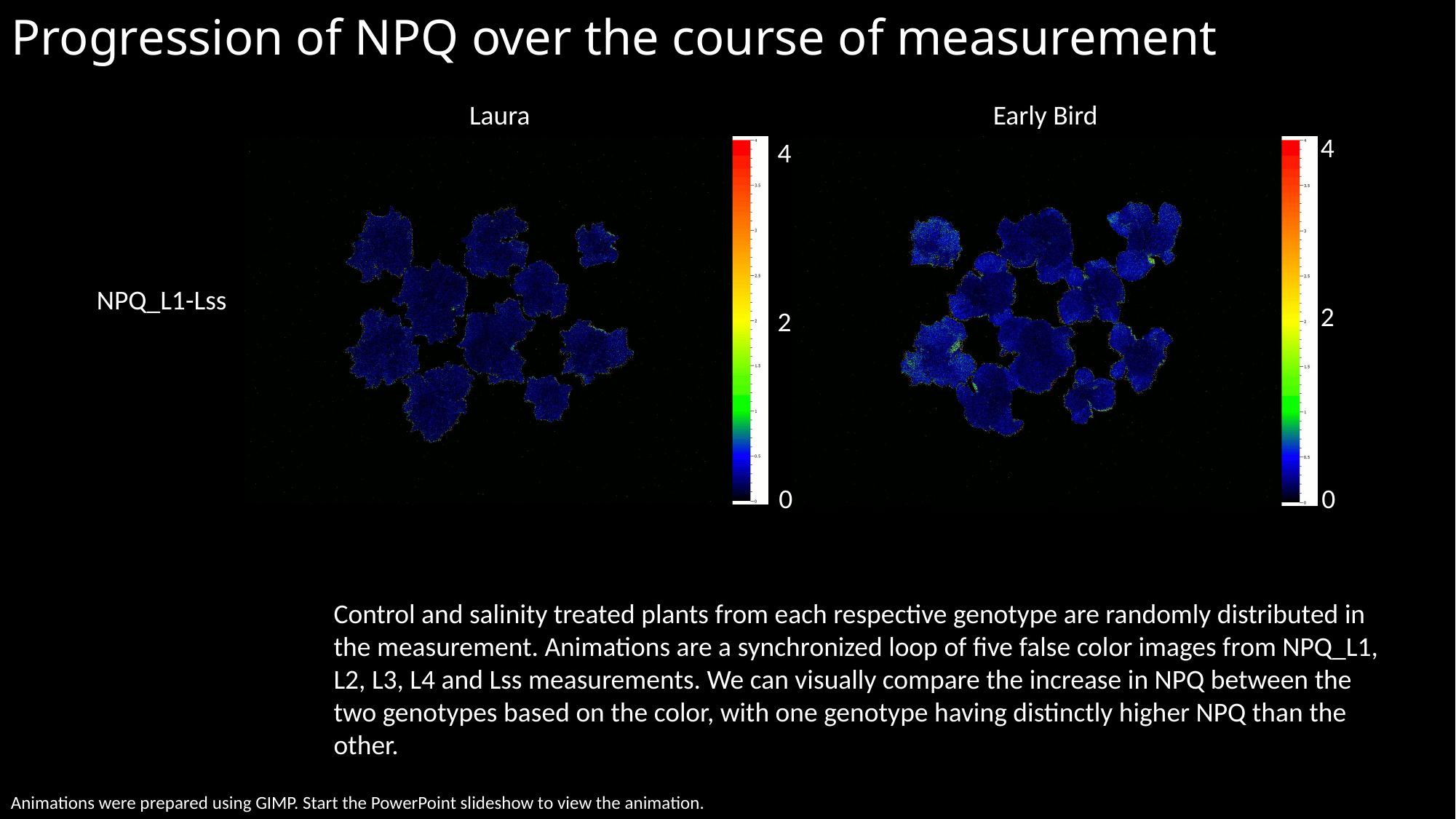

# Progression of NPQ over the course of measurement
Laura
Early Bird
4
4
NPQ_L1-Lss
2
2
0
0
Control and salinity treated plants from each respective genotype are randomly distributed in the measurement. Animations are a synchronized loop of five false color images from NPQ_L1, L2, L3, L4 and Lss measurements. We can visually compare the increase in NPQ between the two genotypes based on the color, with one genotype having distinctly higher NPQ than the other.
Animations were prepared using GIMP. Start the PowerPoint slideshow to view the animation.

## Slide 2
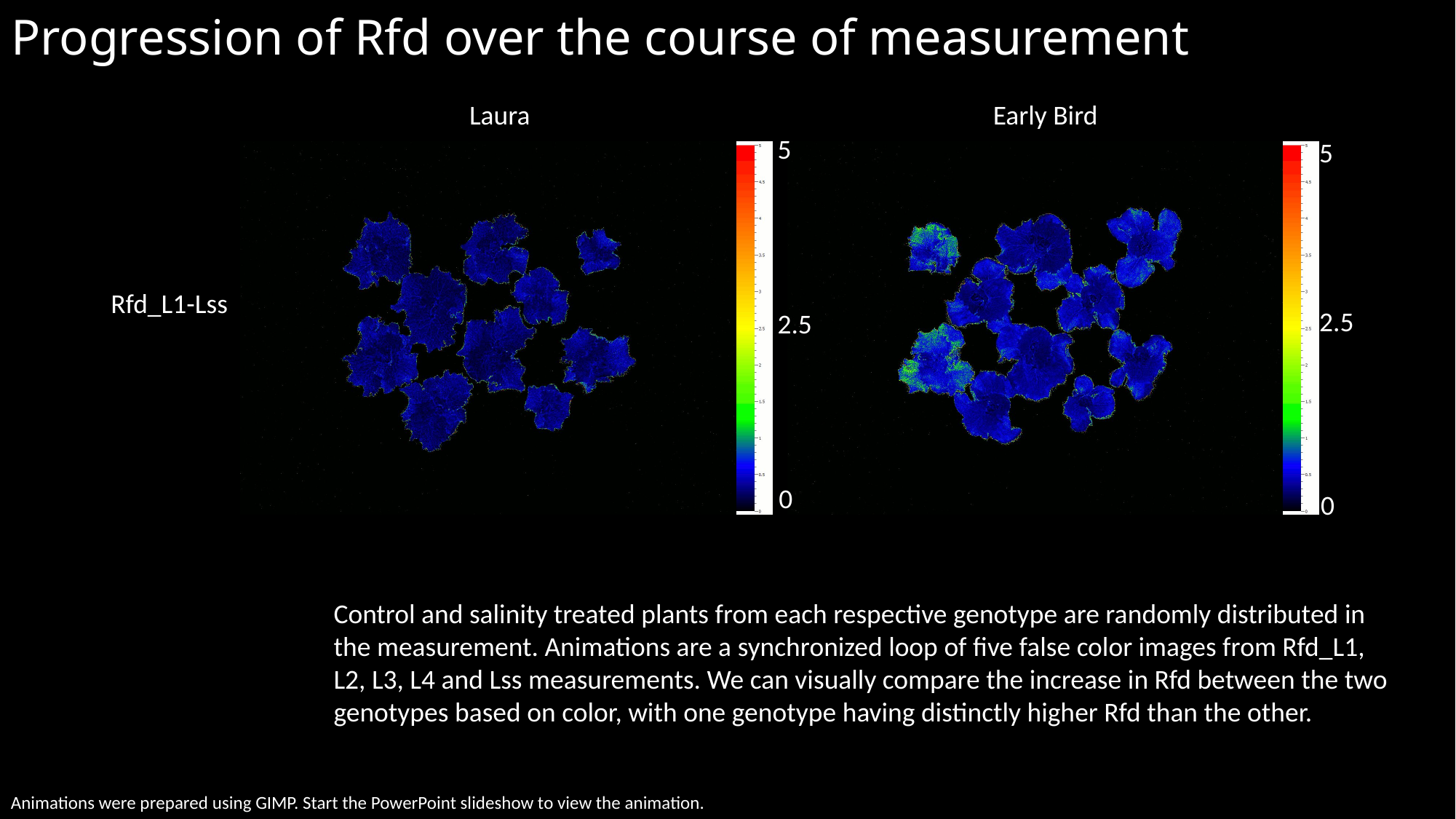

# Progression of Rfd over the course of measurement
Laura
Early Bird
5
5
Rfd_L1-Lss
2.5
2.5
0
0
Control and salinity treated plants from each respective genotype are randomly distributed in the measurement. Animations are a synchronized loop of five false color images from Rfd_L1, L2, L3, L4 and Lss measurements. We can visually compare the increase in Rfd between the two genotypes based on color, with one genotype having distinctly higher Rfd than the other.
Animations were prepared using GIMP. Start the PowerPoint slideshow to view the animation.

## Slide 3
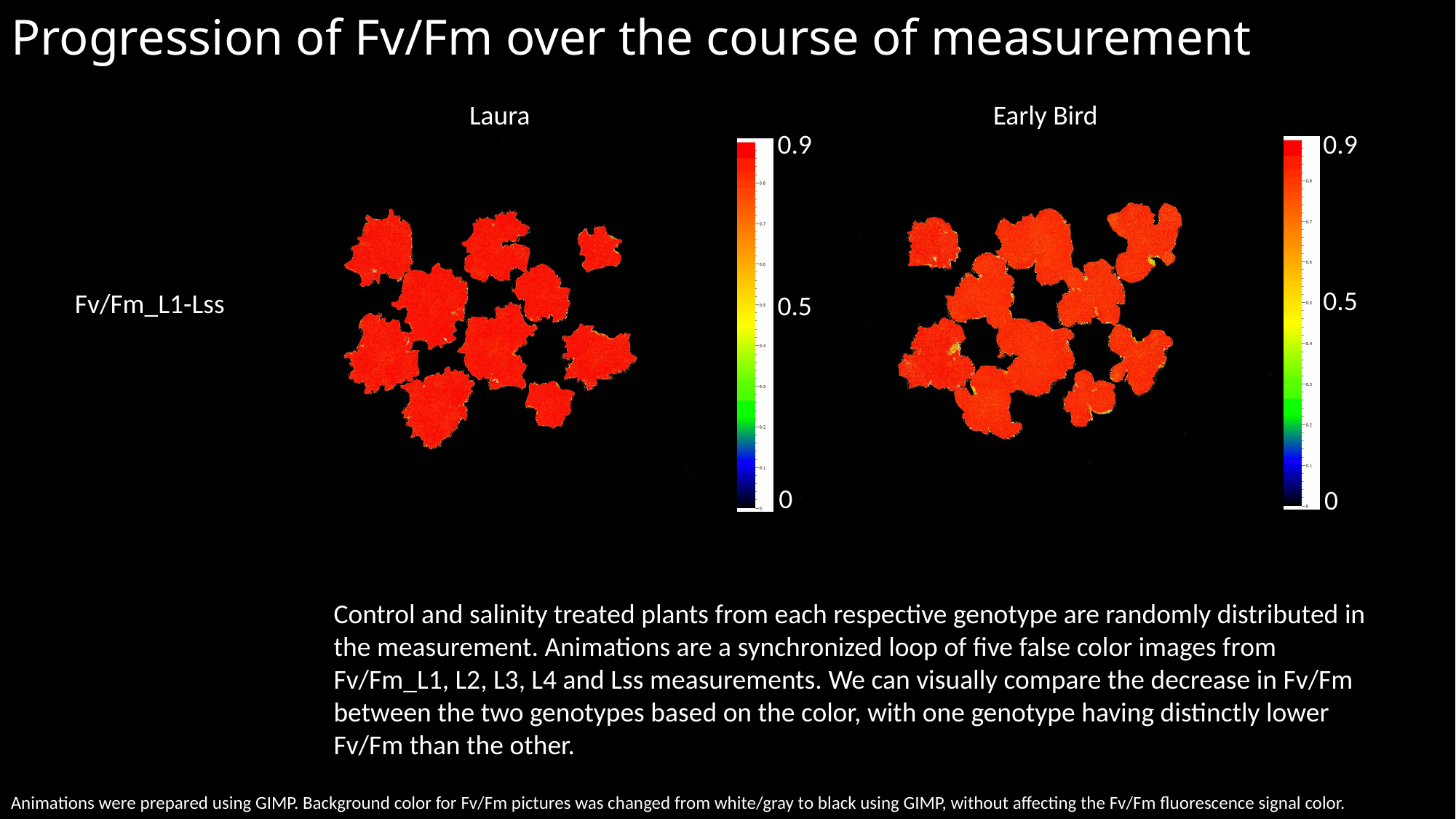

# Progression of Fv/Fm over the course of measurement
Laura
Early Bird
0.9
0.9
0.5
Fv/Fm_L1-Lss
0.5
0
0
Control and salinity treated plants from each respective genotype are randomly distributed in the measurement. Animations are a synchronized loop of five false color images from Fv/Fm_L1, L2, L3, L4 and Lss measurements. We can visually compare the decrease in Fv/Fm between the two genotypes based on the color, with one genotype having distinctly lower Fv/Fm than the other.
Animations were prepared using GIMP. Background color for Fv/Fm pictures was changed from white/gray to black using GIMP, without affecting the Fv/Fm fluorescence signal color.
